# What is well-being? A scoping review of the conceptual and operational definitions of occupational well-being

**DOI:** 10.1017/cts.2023.648

**Published:** 2023-10-16

**Authors:** Tara G. Bautista, Gretchen Roman, Munziba Khan, Michele Lee, Sumeyra Sahbaz, Lunthita M. Duthely, Alexa Knippenberg, Miracle A. Macias-Burgos, Alec Davidson, Carolina Scaramutti, Janice Gabrilove, Susan Pusek, Darshan Mehta, Miriam A. Bredella

**Affiliations:** 1 Northern Arizona University, Flagstaff, AZ, USA; 2 University of Rochester Medical Center, Rochester, NY, USA; 3 National Center for Advancing Translational Sciences, Bethesda, MD, USA; 4 University of Texas, Austin, OK, USA; 5 University of Miami School of Medicine, Miami, FL, USA; 6 Icahn School of Medicine at Mount Sinai, New York, NY, USA; 7 University of North Carolina School of Medicine, St. Chapel Hill, NC, USA; 8 Harvard Medical School, Boston, MA, USA; 9 New York University Langone Health, New York, NY, USA

**Keywords:** Well-being, occupational health, workforce, assessment, review

## Abstract

Well-being is a multifaceted construct that is used across disciplines to portray a state of wellness, health, and happiness. While aspects of well-being seem universal, how it is depicted in the literature has substantial variation. The aim of this scoping review was to identify conceptual and operational definitions of well-being within the field of occupational health. Broad search terms were used related to well-being and scale/assessment. Inclusion criteria were (1) peer-reviewed articles, (2) published in English, (3) included a measure of well-being in the methods and results section of the article, and (4) empirical paper. The searches resulted in 4394 articles, 3733 articles were excluded by reading the abstract, 661 articles received a full review, and 273 articles were excluded after a full review, leaving 388 articles that met our inclusion criteria and were used to extract well-being assessment information. Many studies did not define well-being or link their conceptual definition to the operational assessment tool being used. There were 158 assessments of well-being represented across studies. Results highlight the lack of a consistent definitions of well-being and standardized measurements.

## Introduction

Well-being is a multifaceted construct and while there is no consensus on a single definition, the Centers for Disease Control and Prevention (CDC) describes well-being as “the presence of positive emotions and moods, the absence of negative emotions, satisfaction with life, fulfillment, and positive functioning [1].” The interest in studying well-being within health research has drastically increased over the last 20 years. Using the PubMed database, there were 1,361 results using the term well-being in 2003, in 2022 there were 22,536 results for the term well-being. While use of the term has increased, we have not seen the same attention applied to defining the term comparably across fields of study. Colloquially, well-being is often defined or discussed as a synonym for wellness, health, happiness, and satisfaction. Within the academic community, we define well-being as a multifaceted construct with definitions that vary by domain. For example, the definition of emotional well-being will differ from the definition of physical well-being or economic well-being. Although aspects of well-being seem universal, how it is depicted in the literature has substantial variation in definition and even greater variation in how it is measured.

Specifically, within the field of occupational health and well-being, we have also seen an increase in the interest in measuring and improving workers’ well-being. In 2011, the National Institute of Occupational Safety and Health within the CDC expanded the traditional delivery of occupational safety and health by integrating well-being [2]. Total Worker Health® was introduced as a strategy that combines health protection with health promotion to prevent worker injury and advance well-being [3]. The recent coronavirus disease 2019 pandemic has brought even greater attention to the importance of worker well-being. Much concern has been specifically expressed about the mental health and well-being of healthcare professionals during and at the height of the pandemic [1]. However, psychological distress from the pandemic on the overall workforce has led to greater turnover intention [4], resignation [5], and ultimately, labor shortages. So much so that in 2022, the U.S. Surgeon General released a new framework for mental health and well-being in the workplace, stating that it is “a critical priority for public health [6].” Protection from harm, connection and community, work-life harmony, mattering at work, and opportunities for growth were the five essentials that were highlighted to guide leaders in developing an organizational culture that supports worker mental health and well-being [6]. Therefore, the purpose of this paper is to identify conceptual definitions and operational assessments of well-being within the field of occupational health.

## Methods

Studies were identified by searching PubMed September 2022 and April 2023 using the search terms “well-being,” “occupational OR workplace,” and “scale.” The inclusion criteria were (1) scholarly journal articles, (2) published in English, (3) measured well-being, and (4) empirical papers. The search was not limited by date of publication. From this search, 4394 articles were identified. After reviewing the abstracts, 3733 articles were removed for not having a well-being measure, leaving 661 articles for full review. Four reviewers conducted the screening using pre-established inclusion criteria. In the first screening, reviewers independently screened the abstracts for inclusion criteria. If one reviewer indicated an article as relevant at the initial screening phase, the article proceeded to the second screening to ensure inclusivity. In the second screening, reviewers independently screened the full text of articles to ensure the articles met the inclusion criteria. Following a full review of the articles, 273 papers were removed, leaving 388 articles included in the data extraction for the present study.

### Data Extraction

The following information was extracted from the 388 articles that met the inclusion criteria: (a) Well-being assessment citation, (b) name of the well-being assessment, (c) items and rating, (d) reliability and validity, (e) samples/populations from occupational health and well-being studies, (f) assessment limitations noted in the occupational health and well-being studies, and (g) other well-being assessments used in combination with this assessment. This information was condensed by assessment so that the same assessment was only listed once. This information is displayed in Table 1.

**Table 1.** Well-being assessments used in the occupational health and well-being literature

Well-being assessment citation	Name of assessment	Items and rating	Reliability/ validity	Samples/Population(s) from occupational health and well-being studies	Assessment limitations noted in the occupational health and well-being studies	Other well-being assessments used in combination
Tibblin *et al*., 1990 [8]	Goteborg Quality of Life Instrument (QoL): Well-Being Scale	19 items; rated 1–7	*α* = 0.72–0.89	Middle-aged woman. [9]; This study investigates 108 male workers in Sweden. [10]		
Dyrbye *et al*., 2013 [11]	Mayo Clinic Physician Well-being Index	Seven items; rated yes/no	*α* = 0.83	Physicians and APPs employed by the Health Texas Provider Network [12]	Cross-sectional, self-selection bias, limited external validity, unmeasured covariate bias [12]	Connor-Davidson Resilience Scale [13,14]; Interpersonal Reactivity Index [15]
Dyrbye *et al*., 2010 [16]	Medical Students Well-Being Index (MSWBI)	Seven “yes or no” questions; rated one or zero. MSWBI scores range from zero to seven, and seven points indicate the greatest level of distress.	*α* = 0.68; The majority of MSWBI items had *a* ≥74% sensitivity and specificity for detecting distress within the intended domain.	Medical students of all years (1st–6th) from all 22 Polish medical schools. [17]		
Butler & Kern, 2016 [18]	PERMA scale (multideimensional model of well-being)	Five dimensions: Positive Emotion (P), Engagement (E),Relationships (R), Meaning (M),Accomplishment (A). Twenty-three items, 11 point Likert scale (0–10) used Japanese version	*α* =0.75–0.96	310 workers completed questionnaire online. [19]		Brief Job Stress Questionnaire [20], Utrecht Work Engaement Scale [21]
Lau *et al*., 2005 [22]	Personal well-being index – Chinese version and English version	Seven items; 0 (completely dissatisfied)–10 (completely satisfied); normative values range between 60 and 70	*α* = 0.73–0.85	Community dwelling older persons with mild dementia in Hong Kong.[26]; Adults with chronic SCI aged 70 years or less [27]; The NZAVS is an ongoing 20-year national longitudinal panel study of social attitudes, personality, and health outcomes that beganin 2009. Thes ample analyzed in the current study involve participants who completed the survey during the nation wide Level 4 lockdown (March 25 through April27, 2020) as well as around the same time period the prior year in the tenth wave of the study. [28]; 1190 hospital midwives from 7 countries (2 Asian, 5 Europe). [29]	Small sample size, lack of community resources in dementia care, low minimum requirements for caregiver visits, low literacy level in spouse caregivers, and the noncompliance of participants with dementia, the program might also have inadvertently increased the burden to the family caregiver. [26] Small sample, participants were self-selected, lack of blinding in the study/ every participant was aware of their allocation [27]	The Depression, Anxiety and Stress Scale short version [30]; Spinal Cord Lesion Emotional Well-being Questionnaire version 1 Australia [31]
Lau *et al*., 2008 [23]						
Cummins *et al*., 2004 [24]						
Cummins *et al*., 2003 [25]						

Chassany *et al*., 2004 [32] Dagenais-Desmarais & Savoie, 2012 [33]	Psychological General Well-Being Index (PGWBI): Subscales: Interpersonal Fit at Work, Perceived Recognition at Work, Thriving at Work, Involvement at Work, and Feeling of Competency at Work	22 items; rated 0–5	*α* = 0.70–0.85	Online survey, in nine tertiary educational institutions, eight Universities in Australia and one in New Zealand. [34]; Four public institutions in Gabon (Africa). [35]	Small sample. [34]	Short Almost Perfect Scale [36]
Kaplan *et al* 1997 [37]; Kaplan & Anderson, 1998 [38]	Quality of Well-being, Self-Administered (QWB-SA)	It has 5 scales and 58 questions using a dichotomous scale. The Quality of Well-being scale is a preference-weighted measure combining three scales of functioning with a measure of symptoms and problems to produce a point-in-time expression of well-being that runs from 0 (for death) to 1.0 (for asymptomatic full function).		218 English speaking adults who attended primary care clinics. 86 participants with combat-related major lower-limb amputatio. [39]; 75 staff in ambulatory care environment in United States. [40]		
Ryff, 1989 [41]; Palma-Candia *et al*., 2019 [42]; Toledano-Gonzales *et al*., 2019 [43]; Jang *et al*., 2019 [44] Sirigatti *et al*., 2009 [45]	Ryff Psychological Well-Being Scale; Ryff PWB (Spanish Version); Ryff PWB (Korean Version) Ryff PWB (Italian Version)	42 items; rated 1–7 6 subscales: self-acceptance, positive relations with others, autonomy, environmental mastery, purpose in life, and personal growth. Spanish Version: 39 items; rated 1–7. Korean Version: 46 items; rated 1–7	*α* = 0.86–0.93 *α* = 0.78–0.81 (Spanish Version) *α* = 0.66-0.76 (Korean Version)	Older Adults in Magallanes, Chile. [42]; 74 older adults living in retirement homes. [43]; 399 Korean Nurses in a university hospital. 1142 people who report having multiple sclerosis. [46]; 2102 community residents in Tokyo aged 30.	Not possible to obtain two groups of similar sociodemographic characteristics. [42]; Small sample size. [43]; Only one hospital, and only for nurses; Self-reported, cross-sectional. [46]	Satisfaction with Life Scale [47]
Ware *et al*., 1996 [48]Al Sayah *et al*., 2013 [49] Pickard *et al*., 1999 [50]	Short Form 12 (SF-12; produced physical component score for physical well-being and mental component score for mental well-being) - Italian version or the Medical Outcomes Study Short Form (SF-12)	12 items; some rated yes/no, others rated excellent to poor, not at all to extremely	SF-12 achieved a R^2^ of 0.911 in the prediction of PCS-36 and 0.918 in the prediction of MCS-36	Nurses, physicians, residents, and medical and nursing students in the oncology-hematology units of 3 teaching hospitals in Rome. [51]; 1871 Australian veterans who served in the Gulf region during the period August 2, 1990 to September 4, 1991 [52]. University professors of all majors in Jordan [53]. 254 individuals who met criteria for mild stroke at Washington University	Cross-sectional and self-report. [51] Only included probable musculo skeletal conditions, these were not confirmed by a diagnostic process or a validity study. [52]; An online survey which could have limited generalizability. [53]	Depression Anxiety Stress Scale (DASS) [30,54,55]Neck Disability Index [56,57]; The International Physical Activity Questionnaire [58–60]
Migliorini *et al*., 2008 [31]	Spinal Cord Lesion Emotional Well-being Questionnaire version 1 Australia (three domains: helplessness, intrusion and personal growth)	12 items; rated 1 = strongly disagree, 2 = disagree, 3 = agree or 4 = strongly agree	*α* = 0.78	Adults with chronic SCI aged 70 years or less. [27]	Small sample, participants were self-selected, lack of blinding in the study/ every participant was aware of their allocation. [27]	The Depression, Anxiety and Stress Scale short version [30]; Personal Well-being Index–Adult [24]
Paloutzian & Ellison 1991 [61]	Spiritual Well-Being Scale	20-item Likert scale; two subscales: religious well-being and existential well-being. sum of two subscales is total spiritual well-being score, with higher total score indicating higher spiritual well-being.	*α* = .0.87	Relations of spiritual well-being, globall job satisfaction, and general self-efficacy to hope in 64 Continuing Care Assistants. [62]		
Malinakova *et al*., 2017 [63]	Spiritual Well-Being Scale-Shortened version	Seven items, six point Likert Scale ranging from strongly agree to strongly disagree	*α* = 0.814	A nationally representative sample (4217) of Czech adolescents [63]; 4182 Czech adolescents [64]	self-report, problems with some of the items on the scale which created a separate factor	
Petride et al., 2003 [65] Stamatopoulou *et al*., 2016 [66]	Trait Emotional Intelligence Questionnaire-Short Form (TEIQue-SF): Subscales 1) Well Being, 2) Self-Control, 3) Emotionality, and 4) Sociability	30 items; rated 1–7	*α* = 0.52–0.85	nurses working in both public (52.9%) and private (47.1%) health units in Greece. [67]		Boredom Proneness Scale Farmer & Sundberg, 1986 [68]
Watanabe *et al*., 2020 [69]	University of Tokyo Occupational Mental Health (TOMH) Well-being Scale	24 items; rated 1–4	*α* = 0.671–0.845	Japanese workers [69]	Selection bias, may have been errors inmeasuring assessment of the standards of convergent validity, not generalizabile to workers from other cultural backgrounds [69]	
Tennant *et al*., 2007 [70]	Warwick-Edinburgh Mental Well-being Scale (WEMWBS)	14 items; rated 1–5	*α* = 0.89 (student sample); *α* = 0.91 (population sample)	Students, working adults, and patients (one psychiatric population composed of patients with remitted schizophrenia). [71]; Sport coaching networks across Australia. [72]; Office employees at each of four Spanish universities in Galicia, the Basque Country and Catalonia. [73]; A large stratified random sample of veterinary surgeons practising in the UK. [74]; Sit less, move more intervention was assessed at 6 Spanish University campuses. The intevention had no effect on mental well-being. [73]; 174 Australian junior cricket players attending either the male U19 National Championships or the female U18 National Championship. [75]; 13 homeless in Canada. [76]; 424 mental health employees and manager. [77]	Ex-coaches who have burnout not represented (survival bias) and lack of personal and social variables measured known to effect mental well-being. [72]; Evidence to support a range of psychometric properties for the comparator scales (i.e., Short Warwick-Edinburgh Mental Well-being Scale) is restricted to samples of other populations. [74]	
Taggart *et al*., 2013 [78]; Bartram *et al* 2011 [79]; Tennant *et al*., 2007 [70]	Warwick-Edinburgh Mental Well-Being Scale (WEMWBS) -English version	14 items; rated 1–5	*α* = 0.88–0.94	Pakistani healthcare professionals. [80]; UK veterinarians. [81]; 126 patients in Hong Kong. [82]; 148 employees at 2 emergency departments in Queensland, Australia. [83]; 744 students studying veterinary medicine, medicine, dentistry, pharmacy and law in the UK [84]	Not translated into Urdu the official language of Pakistan, convenience sample, data only collected from Punjab, cannot be generalized to the whole Pakistani population, occupational stress was measured very subjectively by presence or absence, rather than with a cross-culturally validated scale, healthcare providers were not inquired about their psychiatric health using scales for common mental illnesses. [80]; Nursing staffin a hospital in the city Kaunas, Lithuania. [85]; 72 healthy elderly people in Canada. [86]	Hospital and Anxiety Depression Scale[87]; Health and Safety Executive Management Standards Indicator Tool. [88]; Questions on suicidal ideation. [89]; Alcohol Use Disorders Identification Test. [90]
Schlosser, 1990 [91]	Well Being Scale (WBS-36).	36 items on a 5-point scale.	0.94 when tested on 178 healthy individuals	39 critically injured patients in Canada.		
Myers *et al*., 2020 [92]; Myers *et al*., 2008 [93]	Well-Being Actions Self-Efficacy Ssale (interpersonal, community, occupational, physical, psychological, and economic)	18 items; rated 0– 4	Intraclass correlation coefficients ranged from 0.75 (interpersonal) to 0.84 (occupational)	Adults with obesity or overweight [92]	Self-report [92]	Expanded version of the I COPPE action scale [92,94]
Williams *et al*., 2017 [95]	Well-Being Process Questionnaire (work characteristics, individual differences, personalisty, outcomes)	25 items; 1–10, strongly disagree to agree strongly (except for stress, rated 1–5)	Average estimated reliability for the single-item measures in this study was above the 0.50 level and a range of items from demands to self-esteem and positive mood were above 0.70	120 university staff members aged 20-64. [96]; 3164 Irish physicans. [97]	A more representative sample of the general population, canonly note casual relations, the fact that DASS-21 measured emotional states rather than diagnostic categories may be observed as a limitation	Self-Rated health [98]; The General Health Questionnaire (GHQ-12) [99]; Depression, Anxiety, Stress Scale (DASS-21) [30], one item of self-stigma
Bech *et al*., 2003 [100]; Bonsignore *et al*., 2014 [101] Topp *et al*., 2015 [102]	WHO-5 Well-Being Index WHO-5 Well-Being Score (Danish version)	5 items; rated 0–100	*α* = 0.80–0.91; good construct validity and acceptable sensitivity (M = 0.86) and specificity (M = 0.81)	463 volunteers from Boston Medical Center, Boston University, and EMC and other employed adults.[103]; Health professionals (majority female nurses working at a university). [104]; Employees at a major tertiary healthcare institution (the Mayo Clinic) [105]; Occupational therapy practitioners and students [106]; Healthcare Workers in Saudi Arabia During the COVID-19 Pandemic [107]; German Emergency Medical Service Workers [108]; Residents in nursing homes [109]; 10 patients receiving spasticity treatment including botulinum toxin injection and physiotherapy and/or occupational therapy. [110]; Patients with epilepsy aged≥15 years from three outpatient clinics in Central Denmark Region. [111]; Persons with chronic suicidality as the primary presenting complaint. [112]; RCT 67 individuals with type two diabetes. [113]; Australian workplace adults. [114]; Stress-afflicted long-term sickness benefit beneficiaries in Denmark. [115]; 60 GPs and registrars working in either a full-time or part-time capacity in Emerald, Queensland. [116]; 231 physiotherapy patients with musculoskeletal disease, response rate to well-being questionnaire was 66. [117]; 93 leaders of different professions from a tertiary hospital in Germany. [118]; Arabic version of WHO-5 was used with 200 patients from six rural PHC settings in the Ismailia, Egypt governorate. [119]; 1,164 employees nested in 30 workplaces in Canada.[120]; 237 residents from 6 communities in Shanghai. [121]; 545 Danish child protection workers. [122]; 126 paitents in Hong Kong. [82]; 169 volunteers in United Kingdom [123]; 502 university employee in United Kingdom. [124]; 147 employees at a insurance company in Germany. [125]	Recruitment problems, which led to the study’s being under powered to detect behavior change in a randomized intervention trial.[103]; Single healthcare setting and small sample size [105]; Convenience sampling [106]; Healthcare workers who do not have internet access or were not familiar with online platforms were not represented [107]	6-item Gratitude Questionnaire [126]; Neff’s Self-Compassion scale (12 items) [127]; Confidence in providing Compassionate Care Scale (10 items) [128]; Depression Anxiety Stress Scale (DASS) [30]; Self-Compassion Scale [127]; Quality of Life in Dementia Scale (QUALID) [129]
Parker & Hyett, 2011 [130]	Work Well-Being Questionnaire	31 items; rated 0–5		1206 patients from an internet site called black dog insitutite. Majority female. [130]		

## Results

There was a total of 158 well-being assessments that resulted from our scoping review of the occupational health and well-being literature. The full table is available as an online supplemental file. A condensed version of the table with the most relevant well-being assessments (*N* = 21) can be found in Table 1. Of the information extracted, three sections are highlighted in this section (1) reliability and validity of the well-being assessments, (2) the samples/populations represented within the occupational health and well-being articles included in this review, and (3) limitations of the assessments noted in the included studies.

### Reliability and Validity

The most common psychometric information reported was internal consistency or reliability represented by Cronbach’s alpha (*α*). There were a few single-item measures and items rated as yes/no, for which reliability cannot be tested. The Cronbach’s alpha coefficient is meant to provide a measure of the internal consistency. The coefficient ranges between 0 and 1, with a value of 0.70 or higher indicating good consistency and reliability [7]. While reliability should be determined before validity, reliability alone does not tell us if the measure is also valid. We found that few assessments reported the validity and for those that did, the type of validity varied (i.e., predictive, discriminant, convergent, etc.). Therefore, we have little information about whether the assessments that claim to measure well-being are valid measures of the construct.

### Populations

There were a variety of samples and populations represented across assessments. While we limited our study to only articles published in English, there were a range of countries represented across studies. We also had a variety of employment types reported across studies, with the majority being from healthcare or healthcare-related fields.

### Limitations

We extracted the author-reported limitations of their study using the well-being assessment. The listed limitations were specific to the potential impact of the assessment of well-being. The most frequent limitations mentioned were the lack of generalizability of the well-being findings to other populations and small sample sizes. These are both subjective to the author’s perspective, but we believe these are worth considering when choosing a well-being assessment appropriate for each study.

## Discussion

The overall lack of attention to the measurement and assessment of well-being and use of inconsistent types of measures of well-being in published articles is concerning. Most studies resulting from our search did not properly report how they assessed well-being. A deficiency in the use of a standard definition may, in part, explain the heterogeneity of well-being measurements that were reported. Utilization of a standardized definition and shared conceptual framework may help researchers develop strong measurements that accurately depict and report well-being.

### Populations Represented in Occupational Health and Well-Being Literature

A unique feature of our scoping review was the extraction of information related to the samples and populations that have been included when measuring occupation/workplace well-being. We purposely allowed for a wide variation in populations to gather information regarding international assessments of well-being, but we were limited by only being able to review studies published in (or translated to) English. At first glance, we did not notice a difference in how well-being was assessed between cultures, but future studies may be able to use the data we extracted and presented in Table 1 to perform a more formal analysis to assess potential differences in well-being assessments between cultures.

### Recommendations for Assessing Well-Being

Based on the extensive review of over 300 articles, we have developed three recommendations for researchers who want to improve their well-being assessment. First, we were surprised at the number of articles that had to be excluded following a full review because they did not actually measure well-being despite discussing well-being in the introduction sections and having concluding remarks in their discussion sections. We recommend that authors do not mention well-being unless they have measured it and if they use a composite of measures, we recommend they explain how the composite operationalizes well-being. Second, be as precise as possible in your conceptual definition of well-being. We saw multiple articles that used a broad conceptual definition of well-being, but then a specific and narrow operational definition or assessment tool. We recommended researchers introduce a specific definition of well-being (e.g., economic, emotional, physical, spiritual) in their introduction section that will help the readers understand which domain of well-being is being assessed. We recommend using subscales or focused scales when measuring specific domains of well-being, such as emotional well-being. We do not recommend concluding emotional well-being based on an overall well-being assessment. We suggest that the term well-being only be used when multiple constructs are used together to assess an overall composition of well-being, beyond what can be captured through a single aspect of well-being. It is clear across conceptual definitions that well-being is a multifaceted construct that cannot be captured through a single dimension. When using a single construct to assess well-being, we suggest defining the individual construct rather than using the single construct to define well-being. For example, if a researcher is measuring quality of life, happiness, and health, those combined measures could be used to infer well-being, or they may be using a multidimensional well-being scale. But, if the study is only measuring quality of life, then the researcher should only infer quality of life, not well-being. Across fields, it is imperative to procure validated instruments that accurately measure well-being and reflect participants’ data accurately.

### Limitations and Future Research

The results of the present study should be interpreted with the following limitations in mind. Due to the overwhelming large scope of research that could be included with the simple term “well-being,” we are not able to present the full body of research in a single scoping review, therefore we decided to focus specifically on occupational well-being. The present study was limited to empirical studies indexed in PubMed and published in the English language only.

We would also like to acknowledge the potential issue of false positive and false negative when searching for articles that measure well-being. There may have been false positive results by including articles that do not directly measure well-being, but conclude well-being based on proxy measures of mental and physical health. These articles are falsely included because the authors use the term “well-being” and therefore the article was found during our searches. Additionally, there may have been false negative results by missing articles that did not use the term “well-being” to describe their results, but based on their measure, we would have defined their construct as measure of well-being. Because the authors did not use the term well-being, their article was not a result of our searches.

There were many articles that discussed well-being in the introduction and discussion but did not measure well-being in the methods and results. For the purposes of this review, these papers were excluded as they did not provide adequate explanation of the measurement of the well-being construct. Future research may be interested in looking at this issue more specifically and what it means for the field to conclude well-being or make implications for well-being without measuring the construct directly.

Selecting the appropriate assessment of well-being for each study is a challenge and there is currently no standard process for selecting the best assessment tool. This is a promising future avenue of work for researchers interested in creating a flow chart to assist researchers in finding an assessment that fits their study aims and methods. There are currently online repositories hosted by groups such as The University of Connecticut (UConn) M3EWB (Mechanisms Underlying Mind-Body Interventions and Measurement of Emotional Well-Being) Network that allows researchers to find assessments for specific types of well-being. For example, researchers can search these repositories for an emotional well-being assessment for children. These repositories, if maintained, can be an excellent tool for managing the most reliable and valid assessments in the field. We believe the table available as a supplement file and the condensed table presented in this paper are also useful tools for researchers to use to identify a well-being assessment tool that fits the needs of their study. These tables may also be used for future analyses to search for patterns and gaps in current measurement. For example, someone may use these tables to see if there are common limitations across assessments or the most common combination of well-being assessments or look for missing populations and use existing assessments within those populations.

There is a need to clearly define and differentiate the term *well-being* from other constructs to create measures that adequately capture the importance of the term and its antecedents. Assessing if and how well-being differs by cultures and sample characteristics, such as age, education, race and ethnicity, and clinical profile (e.g., disease/disorder, problem severity, comorbidity), could provide valuable insights to improve translational science.

## Conclusion

The current review highlighted the inconsistency of research examining the measurement of well-being. Additional research is needed to develop rigorous measurements of well-being that can be used across study populations and adequately capture the multiple dimensions of well-being. There is a need to provide consistent definitions and precise language when inferring well-being from results.

## Supporting information

Bautista et al. supplementary material Bautista et al. supplementary material

## Supplementary material

The supplementary material for this article can be found at https://doi.org/10.1017/cts.2023.648.

## References

[1] Chutiyami M , Cheong AM , Salihu D , et al. COVID-19 pandemic and overall mental health of healthcare professionals globally: a meta-review of systematic reviews. Front Psychiatry. 2022;12:2600. 10.3389/fpsyt.2021.804525 PMC8801501 35111089

[2] What is Total Worker Health?. 2020. http://www.cdc.gov/niosh/twh/totalhealth.html (Accessed April 1, 2023).

[3] Chari R , Chang C-C , Sauter SL , et al. Expanding the paradigm of occupational safety and health a new framework for worker well-being. J Occup Environ Med. 2018;60(7):589–593. 29608542 10.1097/JOM.0000000000001330 PMC6553458

[4] Poon Y-SR , Lin YP , Griffiths P , Yong KK , Seah B , Liaw SY. A global overview of healthcare workers’ turnover intention amid COVID-19 pandemic: a systematic review with future directions. Hum Resour Health. 2022;20(1):1–18. 36153534 10.1186/s12960-022-00764-7 PMC9509627

[5] Jiskrova GK. Impact of COVID-19 pandemic on the workforce: from psychological distress to the great resignation. J Epidemiol Community Health. 2022;76(6):525–526. 35296522 10.1136/jech-2022-218826

[6] General OotS. The US Surgeon General’s Framework for Workplace Mental Health & Well-Being . Washington, DC: US Public Health Service, 2022.

[7] Tavakol M , Dennick R. Making sense of cronbach’s alpha. Int J Med Educ. 2011;2:53–55. 28029643 10.5116/ijme.4dfb.8dfd PMC4205511

[8] Tibblin G , Tibblin B , Peciva S , Kullman S , Svärdsudd K. The Göteborg quality of life instrument"--an assessment of well-being and symptoms among men born 1913 and 1923. Methods and validity. Scand J Prim Health Care. 1990;1:33–38. 2100362

[9] Håkansson C , Eklund M , Lidfeldt J , Nerbrand C , Samsioe G , Nilsson PM. Well-being and occupational roles among middle-aged women. Work. 2005;24(4):341–351. 15920309

[10] Cederlund R , Iwarsson S , Lundborg G. Quality of life in Swedish workers exposed to hand-arm vibration. Occup Ther Int. 2007;14(3):156–169. 17624874 10.1002/oti.231

[11] Dyrbye LN , Satele D , Sloan J , Shanafelt TD. Utility of a brief screening tool to identify physicians in distress. J Gen Intern Med. 2013;28(3):421–427. 23129161 10.1007/s11606-012-2252-9 PMC3579983

[12] Waddimba AC , Bennett MM , Fresnedo M , Ledbetter TG , Warren AM. Resilience, well-being, and empathy among private practice physicians and advanced practice providers in Texas: a structural equation model study. Mayo Clin Proc Innov Qual Outcomes. 2021;5(5):928–945. 34585086 10.1016/j.mayocpiqo.2021.08.009 PMC8456060

[13] Campbell-Sills L , Stein MB. Psychometric analysis and refinement of the connor-davidson resilience scale (CD-RISC): validation of a 10-item measure of resilience. J Trauma Stress. 2007;20(6):1019–1028. 18157881 10.1002/jts.20271

[14] Connor KM , Davidson JR. Development of a new resilience scale: the connor-davidson resilience scale (CD-RISC). Depression Anxiety. 2003;18(2):76–82. 12964174 10.1002/da.10113

[15] Brock C , Salinsky J. Empathy: an essential skill for understanding the physician-patient relationship in clinical practice. Fam Med. 1993;25(4):245–248. 8319851

[16] Dyrbye LN , Szydlo DW , Downing SM , Sloan JA , Shanafelt TD. Development and preliminary psychometric properties of a well-being index for medical students. BMC Medil Educ. 2010;10(1):1–9. 10.1186/1472-6920-10-8 PMC2823603 20105312

[17] Forycka J , Pawłowicz-Szlarska E , Burczyńska A , Cegielska N , Harendarz K , Nowicki M. Polish medical students facing the pandemic—Assessment of resilience, well-being and burnout in the COVID-19 era. PLoS One. 2022;17(1):e0261652. 35073318 10.1371/journal.pone.0261652 PMC8786167

[18] Butler J , Kern ML. The PERMA-profiler: a brief multidimensional measure of flourishing. Int J Wellbeing. 2016;6(3):1–48. doi: 10.5502/ijw.v6i3.526.

[19] Yang C-C , Watanabe K , Kawakami N. The associations between job strain, workplace PERMA profiler, and work engagement. J Occup Environ Med. 2022;64(5):409–415. 34873133 10.1097/JOM.0000000000002455

[20] Shimomitsu T. The Final Development of the Brief Job Stress Questionnaire Mainly Used for Assessment of the Individuals. Ministry of Labour Sponsored Grant for the Prevention of Work-related Illness: The 1999 Report. Tokyo, Japan: Tokyo Medical College, 2000:126–164.

[21] Shimazu A , Schaufeli W , Kosugi S , et al. Work engagement in Japan: validation of the Japanese version of the Utrecht work engagement scale. Appl Psychol. 2008;57(3):510–523.

[22] Lau AL , Cummins RA , Mcpherson W. An investigation into the cross-cultural equivalence of the personal wellbeing index. Soc Indic Res. 2005;72(3):403–430.

[23] Lau AL , Chi I , Cummins RA , Lee TM , Chou K-L , Chung LW. The SARS (Severe acute respiratory syndrome) pandemic in Hong Kong: effects on the subjective wellbeing of elderly and younger people. Aging Ment Health. 2008;12(6):746–760. 19023726 10.1080/13607860802380607

[24] Cummins R. International Well Being Group: Personal Wellbeing Index. Melbourne: Australian Centre on Quality of Life, Deakin University; 2004.

[25] Cummins RA , Eckersley R , Pallant J , Van Vugt J , Misajon R. Developing a national index of subjective wellbeing: the Australian unity wellbeing index. Soc Indic Res. 2003;64(2):159–190.

[26] Lam LC , Lee JS , Chung JC , Lau A , Woo J , Kwok TC. A randomized controlled trial to examine the effectiveness of case management model for community dwelling older persons with mild dementia in Hong Kong. Int J Geriatr Psychiatry. 2010;25(4):395–402. 19606455 10.1002/gps.2352

[27] Migliorini C , Sinclair A , Brown D , Tonge B , New P. A randomised control trial of an internet-based cognitive behaviour treatment for mood disorder in adults with chronic spinal cord injury. Spinal Cord. 2016;54(9):695–701. 26690861 10.1038/sc.2015.221

[28] Low RS , Overall NC , Chang VT , Henderson AM , Sibley CG. Emotion regulation and psychological and physical health during a nationwide COVID-19 lockdown. Emotion. 2021;21(8):1671–1690. 34843308 10.1037/emo0001046

[29] Jarosova D , Gurkova E , Ziakova K , et al. Job satisfaction and subjective well-being among midwives: analysis of a multinational cross-sectional survey. J Midwifery Womens Health. 2017;62(2):180–189. 28419709 10.1111/jmwh.12516

[30] Lovibond PF , Lovibond SH. The structure of negative emotional states: comparison of the depression anxiety stress scales (DASS) with the beck depression and anxiety inventories. Behav Res Ther. 1995;33(3):335–343. 7726811 10.1016/0005-7967(94)00075-u

[31] Migliorini CE , Elfström M , Tonge BJ. Translation and Australian validation of the spinal cord lesion-related coping strategies and emotional wellbeing questionnaires. Spinal Cord. 2008;46(10):690–695. 18332886 10.1038/sc.2008.22

[32] Chassany O , Dimenäs E , Dubois D , Wu A , Dupuy H. The Psychological General Well-being Index (pgwbi) User Manual. Lyon, France: MAPI Research Institute; 2004.

[33] Dagenais-Desmarais V , Savoie A. What is psychological well-being, really? A grassroots approach from the organizational sciences. J Happiness Stud. 2012;13(4):659–684.

[34] Teixeira H , Lalloo R , Evans JL , et al. An exploratory study of perfectionism, professional factors and psychological well-being of dentistry academics. Aust Dent J. 2021;66(2):175–181. 33403695 10.1111/adj.12816

[35] Medzo-M’Engone J , Ntsame Sima M. Psychometric properties of the psychological well-being at work scale in Gabonese public administration. J Evid -Based Soc Work. 2021;18(1):101–115. 10.1080/26408066.2020.1808551 32865128

[36] Rice KG , Richardson CM , Tueller S. The short form of the revised almost perfect scale. J Pers Assess. 2014;96(3):368–379. 24090303 10.1080/00223891.2013.838172

[37] Kaplan RM , Sieber WJ , Ganiats TG. The quality of well-being scale: comparison of the interviewer-administered version with a self-administered questionnaire. Psychol Health. 1997;12(6):783–791.

[38] Kaplan RM , Anderson JP. A general health policy model: update and applications. Health Serv Res. 1988;23(2):203–35. 3384669 PMC1065501

[39] Eskridge SL , Dougherty AL , Watrous JR , et al. Prosthesis satisfaction and quality of life in US service members with combat-related major lower-limb amputation. Prosthet Orthot Int. 2022;46(1):68–74. 34789707 10.1097/PXR.0000000000000054

[40] Wingler D , Hector R. Demonstrating the effect of the built environment on staff health-related quality of life in ambulatory care environments. HERD. 2015;8(4):25–40. 26123967 10.1177/1937586715573745

[41] Ryff CD. Happiness is everything, or is it? Explorations on the meaning of psychological well-being. J Pers Soc Psychol. 1989;57(6):1069–1081.

[42] Palma-Candia O , Hueso Montoro C , Martí-García C , Fernández-Alcántara M , Campos-Calderón CP , Montoya Juarez R. Understanding the occupational adaptation process and well-being of older adults in magallanes (Chile): a qualitative study. Int J Env Res Pub He. 2019;16(19):3640. 10.3390/ijerph16193640 PMC6801773 31569804

[43] Toledano-González A , Labajos-Manzanares T , Romero-Ayuso D. Well-being, self-efficacy and independence in older adults: a randomized trial of occupational therapy. Arch Gerontol Geriatr. 2019;83:277–284. 31132547 10.1016/j.archger.2019.05.002

[44] Jang MH , Gu SY , Jeong YM. Role of coping styles in the relationship between nurses’ work stress and well-being across career. J Nurs Scholarsh. 2019;51(6):699–707. 31621194 10.1111/jnu.12523

[45] Sirigatti S , Stefanile C , Giannetti E , Iani L , Penzo I , Mazzeschi A. Assessment of factor structure of Ryff’s psychological well-being scales in italian adolescents. Bollettino Di Psicologia Applicata. 2009;259(56):30–50.

[46] Schwartz CE , Snook E , Quaranto B , Benedict RH , Vollmer T. Cognitive reserve and patient-reported outcomes in multiple sclerosis. Mult Scler J. 2013;19(1):87–105. 10.1177/1352458512444914 22546847

[47] Diener E , Emmons RA , Larsen RJ , Griffin S. The satisfaction with life scale. J Pers Assess. 1985;49(1):71–75. 16367493 10.1207/s15327752jpa4901_13

[48] Ware JE Jr . A 12-item short-form health survey: construction of scales and preliminary tests of reliability and validity. Med Care. 1996;34(3):220–233. 8628042 10.1097/00005650-199603000-00003

[49] Al Sayah F , Ishaque S , Lau D , Johnson JA. Health related quality of life measures in arabic speaking populations: a systematic review on cross-cultural adaptation and measurement properties. Qual Life Res. 2013;22(1):213–229. 22350531 10.1007/s11136-012-0129-3

[50] Pickard AS , Johnson JA , Penn A , Lau F , Noseworthy T. Replicability of SF-36 summary scores by the SF-12 in stroke patients. Stroke. 1999;30(6):1213–1217. 10356102 10.1161/01.str.30.6.1213

[51] Magnavita N , Sestili C , Mannocci A , et al. Mental and physical well-being in oncology-hematology-unit personnel. Arch Environ Occup Health. 2018;73(6):375–380. 28777706 10.1080/19338244.2017.1361901

[52] Kelsall HL , McKenzie DP , Forbes AB , Roberts MH , Urquhart DM , Sim MR. Pain-related musculoskeletal disorders, psychological comorbidity, and the relationship with physical and mental well-being in gulf war veterans. PAIN®. 2014;155(4):685–692. 24361580 10.1016/j.pain.2013.12.025

[53] Almhdawi KA , Obeidat D , Kanaan SF , et al. University professors’ mental and physical well-being during the COVID-19 pandemic and distance teaching. Work. 2021;69(4):1153–1161. 34420997 10.3233/WOR-205276

[54] Patrick J , Dyck M , Bramston P. Depression anxiety stress scale: is it valid for children and adolescents? J Clin Psychol. 2010;66(9):996–1007. 20694962 10.1002/jclp.20696

[55] Almhdawi KA , Kanaan SF , Khader Y , Al-Hourani Z , Almomani F , Nazzal M. Study-related mental health symptoms and their correlates among allied health professions students. Work. 2018;61(3):391–401. 30373992 10.3233/WOR-182815

[56] Vernon H , Mior S. The neck disability index: a study of reliability and validity. J Manip Physiol Ther. 1991;14(7):409–415. 1834753

[57] Shaheen AAM , Omar MTA , Vernon H. Cross-cultural adaptation, reliability, and validity of the arabic version of neck disability index in patients with neck pain. Spine. 2013;38(10):E609–E615. 23429690 10.1097/BRS.0b013e31828b2d09

[58] Citko A , Górski S , Marcinowicz L , Górska A. Sedentary lifestyle and nonspecific low back pain in medical personnel in north-east Poland. Biomed Res Int. 2018;2018:1–8. 10.1155/2018/1965807 PMC6151221 30271778

[59] Powell KE , Paluch AE , Blair SN. Physical activity for health: what kind? How much? How intense? On top of what? Annu Rev Public Health. 2011;32(1):349–365. 21128761 10.1146/annurev-publhealth-031210-101151

[60] Helou K , El Helou N , Mahfouz M , Mahfouz Y , Salameh P , Harmouche-Karaki M. Validity and reliability of an adapted arabic version of the long international physical activity questionnaire. BMC Public Health. 2018;18(1):1–8. 10.1186/s12889-017-4599-7 PMC5525276 28738790

[61] Paloutzian RF , Ellison CW. Manual for the Spiritual Well-being Scale. Nyack, NY: Life Advance; 1991.

[62] Duggleby W , Cooper D , Penz K. Hope, self-efficacy, spiritual well-being and job satisfaction. J Adv Nurs. 2009;65(11):2376–2385. 19737323 10.1111/j.1365-2648.2009.05094.x

[63] Malinakova K , Kopcakova J , Kolarcik P , et al. The spiritual well-being scale: psychometric evaluation of the shortened version in Czech adolescents. J Relig Health. 2017;56(2):697–705. 27787695 10.1007/s10943-016-0318-4 PMC5320003

[64] Zidkova R , Glogar P , Polackova Solcova I , et al. Spirituality, religious attendance and health complaints in czech adolescents. Int J Env Res Pub He. 2020;17(7):2339. 10.3390/ijerph17072339 PMC7177996 32235661

[65] Petrides KV , Furnham A. Trait emotional intelligence: behavioural validation in two studies of emotion recognition and reactivity to mood induction. Eur J Pers. 2003;17(1):39–57.

[66] Stamatopoulou M , Galanis P , Prezerakos P. Psychometric properties of the Greek translation of the trait emotional intelligence questionnaire-short form (TEIQue-SF). Pers Individ Differ. 2016;95:80–84.

[67] Papathanasiou Fradelos IV , Nikolaou EC , Tsaras E , Kontopoulou K , Malli L , F. Emotional intelligence and professional boredom among nursing personnel in greece. J Pers Med. 2021;11(8):750. 34442394 10.3390/jpm11080750 PMC8400954

[68] Farmer R , Sundberg ND. Boredom proneness--the development and correlates of a new scale. J Pers Assess. 1986;50(1):4–17. 3723312 10.1207/s15327752jpa5001_2

[69] Watanabe K , Imamura K , Inoue A , et al. Measuring eudemonic well-being at work: a validation study for the 24-item the university of Tokyo occupational mental health (TOMH) well-being scale among Japanese workers. Ind Health. 2020;58(2):107–131. 31366851 10.2486/indhealth.2019-0074 PMC7118063

[70] Tennant R , Weich S , Joseph S , et al. The Warwick-Edinburgh mental well-being scale (WEMWBS): Development and UK validation. Health Qual Life Outcomes. 2007;5(63):1–13. 10.1186/1477-7525-5-63. 18042300 PMC2222612

[71] Trousselard M , Steiler D , Dutheil F , et al. Validation of the Warwick-Edinburgh mental well-being scale (WEMWBS) in French psychiatric and general populations. Psychiatry Res. 2016;245:282–290. 27565700 10.1016/j.psychres.2016.08.050

[72] Carson F , Malakellis M , Walsh J , Main LC , Kremer P. Examining the mental well-being of Australian sport coaches. Int J Env Res Pub He. 2019;16(23):4601. 10.3390/ijerph16234601 PMC6926512 31756968

[73] Puig-Ribera A , Bort-Roig J , Giné-Garriga M , et al. Impact of a workplace sit less, move moreprogram on efficiency-related outcomes of office employees. BMC Public Health. 2017;17(1):1–11. 28511642 10.1186/s12889-017-4367-8 PMC5434625

[74] Bartram DJ , Sinclair JM , Baldwin DS. Further validation of the Warwick-Edinburgh mental well-being scale (WEMWBS) in the UK veterinary profession: Rasch analysis. Qual Life Res. 2013;22(2):379–391. 22383106 10.1007/s11136-012-0144-4

[75] Rice SM , Treeby MS , Olive L , et al. Athlete experiences of shame and guilt: initial psychometric properties of the athletic perceptions of performance scale within junior elite cricketers. Front Psychol. 2021;12:581914. 33995169 10.3389/fpsyg.2021.581914 PMC8116891

[76] Marshall CA , McKinley C , Costantini J , Murphy S , Lysaght R , Hart BP. The big island model: resident experiences of a novel permanent supportive housing model for responding to rural homelessness. Health Soc Care Comm. 2022;30(6):e5047–e5061. 10.1111/hsc.13920 35880677

[77] Stansfeld SA , Shipley MJ , Head J , Fuhrer R , Kivimaki M. Work characteristics and personal social support as determinants of subjective well-being. PloS One. 2013;8(11):e81115. 24260545 10.1371/journal.pone.0081115 PMC3834222

[78] Taggart F , Friede T , Weich S , Clarke A , Johnson M , Stewart-Brown S. Cross cultural evaluation of the Warwick-Edinburgh mental well-being scale (WEMWBS)-a mixed methods study. Health Qual Life Outcomes. 2013;11(1):1–12. 23445544 10.1186/1477-7525-11-27 PMC3610169

[79] Bartram DJ , Yadegarfar G , Sinclair JM , Baldwin DS. Validation of the Warwick-Edinburgh mental well-being scale (WEMWBS) as an overall indicator of population mental health and well-being in the UK veterinary profession. Vet J. 2011;187(3):397–398. 20303305 10.1016/j.tvjl.2010.02.010

[80] Ahmad W , Waqas A , Saleem HA , Naveed S. Exploring diet, exercise, chronic illnesses, occupational stressors and mental well-being of healthcare professionals in Punjab, Pakistan. BMC Res Notes. 2017;10(1):1–3. 29258616 10.1186/s13104-017-3096-5 PMC5735512

[81] Summers EM , Morris RC , Bhutani GE , Rao AS , Clarke JC. A survey of psychological practitioner workplace well-being. Clin Psychol Psychother. 2021;28(2):438–451. 32978790 10.1002/cpp.2509

[82] Ng SS , Lo AW , Leung TK , et al. Translation and validation of the Chinese version of the short Warwick-Edinburgh mental well-being scale for patients with mental illness in Hong Kong. East Asian Arch Psychiatry. 2014;24(1):3–9. 24676481

[83] Xu H , Eley R , Kynoch K , Tuckett A. Effects of mobile mindfulness on emergency department work stress: a randomised controlled trial. Emerg Med Australas. 2022;34(2):176–185. 34378320 10.1111/1742-6723.13836

[84] Lewis EG , Cardwell JM. The big five personality traits, perfectionism and their association with mental health among UK students on professional degree programmes. BMC Psychol. 2020;8(1):1–10. 32487181 10.1186/s40359-020-00423-3 PMC7265221

[85] Karpavičiūtė S , Macijauskienė J. The impact of arts activity on nursing staff well-being: an intervention in the workplace. Int J Env Res Pub He. 2016;13(4):435. 10.3390/ijerph13040435 PMC4847097 27104550

[86] Colucci E , Nadeau S , Higgins J , et al. COVID-19 lockdowns’ effects on the quality of life, perceived health and well-being of healthy elderly individuals: a longitudinal comparison of pre-lockdown and lockdown states of well-being. Arch Gerontol Geriat. 2022;99:104606. 10.1016/j.archger.2021.104606 PMC8645291 34896795

[87] Zigmond AS , Snaith RP. The hospital anxiety and depression scale. Acta Psychiatr Scand. 1983;67(6):361–370. 6880820 10.1111/j.1600-0447.1983.tb09716.x

[88] Cousins R , Mackay CJ , Clarke SD , Kelly C , Kelly PJ , McCaig RH. Management standards work-related stress in the UK: practical development. Work Stress. 2004;18(2):113–136.

[89] Paykel ES , Myers JK , Lindenthal JJ , Tanner J. Suicidal feelings in the general population: a prevalence study. Br J Psychiatry. 1974;124(582):460–469. 4836376 10.1192/bjp.124.5.460

[90] Bush K , Kivlahan DR , McDonell MB , Fihn SD , Bradley KA , Project ACQI. The AUDIT alcohol consumption questions (AUDIT-C): an effective brief screening test for problem drinking. Arch Intern Med. 1998;158(16):1789–1795. 9738608 10.1001/archinte.158.16.1789

[91] Schlosser B. The assessment of subjective well-being and its relationship to the stress process. J Pers Assess. 1990;54(1-2):128–140. 2313534 10.1080/00223891.1990.9673980

[92] Myers ND , McMahon A , Prilleltensky I , et al. Effectiveness of the fun for wellness web-based behavioral intervention to promote physical activity in adults with obesity (or overweight): randomized controlled trial. JMIR Form Res. 2020;4(2):e15919. 32130110 10.2196/15919 PMC7075548

[93] Myers ND , Feltz DL , Wolfe EW. A confirmatory study of rating scale category effectiveness for the coaching efficacy scale. Res Q Exerc Sport. 2008;79(3):300–311. 18816941 10.1080/02701367.2008.10599493

[94] Myers ND , Prilleltensky I , Prilleltensky O , McMahon A , Dietz S , Rubenstein CL. Efficacy of the fun for wellness online intervention to promote multidimensional well-being: a randomized controlled trial. Prev Sci. 2017;18(8):984–994. 28303422 10.1007/s11121-017-0779-z

[95] Williams G , Thomas K , Smith A. Stress and well-being of university staff: an investigation using the demands-resources-individual effects (DRIVE) model and well-being process questionnaire (WPQ). Psychology. 2017;8(12):1919–1940.

[96] Williams G , Smith AP. Diagnostic validity of the anxiety and depression questions from the well-being process questionnaire. J Clin Translat Res. 2019;4(2):101–104. PMC6412610 30873498

[97] Hayes B , Prihodova L , Walsh G , Doyle F , Doherty S. What’s up doc? a national cross-sectional study of psychological wellbeing of hospital doctors in Ireland. BMJ Open. 2017;7(10):e018023. 10.1136/bmjopen-2017-018023 PMC5652523 29042389

[98] Ward M , McGee H , Morgan K , et al. One Island–One Lifestyle?. Health and Lifestyles in the Republic of Ireland and Northern Ireland; 2009.

[99] Goldberg D , Bridges K , Duncan-Jones P , Grayson D. Detecting anxiety and depression in general medical settings. Br Med J. 1988;297(6653):897–899. 3140969 10.1136/bmj.297.6653.897 PMC1834427

[100] Bech P , Olsen LR , Kjoller M , Rasmussen NK. Measuring well-being rather than the absence of distress symptoms: a comparison of the SF-36 mental health subscale and the WHO-five well-being scale. Int J Methods Psychiatr Res. 2003;12(2):85–91. 12830302 10.1002/mpr.145 PMC6878541

[101] Krieger T , Zimmermann J , Huffziger S , et al. Measuring depression with a well-being index: further evidence for the validity of the WHO well-being index (WHO-5) as a measure of the severity of depression. J Affect Disord. 2014;156:240–244. 24412323 10.1016/j.jad.2013.12.015

[102] Topp CW , Østergaard SD , Søndergaard S , Bech P. The WHO-5 well-being index: a systematic review of the literature. Psychotherapy Psychosom. 2015;84(3):167–176. 10.1159/000376585 25831962

[103] Farzanfar R , Locke SE , Heeren TC , et al. Workplace telecommunications technology to identify mental health disorders and facilitate self-help or professional referrals. Am J Health Promot. 2011;25(3):207–216. 21192751 10.4278/ajhp.100118-QUAN-14

[104] Rao N , Kemper KJ. Online training in specific meditation practices improves gratitude, well-being, self-compassion, and confidence in providing compassionate care among health professionals. J Evid Based Complementary Altern Med. 2017;22(2):237–241. 27055823 10.1177/2156587216642102 PMC5871184

[105] Mistretta EG , Davis MC , Mh Temkit , Lorenz C , Darby B , Stonnington CM. Resilience training for work-related stress among health care workers: results of a randomized clinical trial comparing in-person and smartphone-delivered interventions. J Occup Environ Med. 2018;60(6):559–568. 29370014 10.1097/JOM.0000000000001285

[106] Popova ES , Hahn J , Morris B , etal H. Exploring well-being: resilience, stress, and self-care in occupational therapy practitioners and students. OTJR (Thorofare N J). 2023;43(2):159–169. 35481402 10.1177/15394492221091271

[107] Abo-Ali EA , Al-Rubaki S , Lubbad S , et al. Mental well-being and self-efficacy of healthcare workers in Saudi Arabia during the COVID-19 pandemic. Risk Manag Healthcare Policy. 2021;14:3167–3177. 10.2147/RMHP.S320421 PMC8328379 34349577

[108] Eiche C , Birkholz T , Jobst E , Gall C , Prottengeier J. Well-being and PTSD in German emergency medical services-a nationwide cross-sectional survey. PLoS One. 2019;14(7):e0220154. 31335903 10.1371/journal.pone.0220154 PMC6650072

[109] Sjölund BM , Mamhidir AG , Engström M. Pain prevalence among residents living in nursing homes and its association with quality of life and well-being. Scand J Caring Sci. 2021;35(4):1332–1341. 33410189 10.1111/scs.12955

[110] Biering-Sørensen B , Iversen HK , Frederiksen IM , Vilhelmsen JR , Biering-Sørensen F. Treatment diary for botulinum toxin spasticity treatment: a pilot study. Int J Rehabil Res. 2017;40(2):175–184. 28225535 10.1097/MRR.0000000000000221 PMC5414540

[111] Schougaard LMV , de Thurah A , Bech P , Hjollund NH , Christiansen DH. Test-retest reliability and measurement error of the Danish WHO-5 well-being index in outpatients with epilepsy. Health Qual Life Outcomes. 2018;16(1):1–6. 30189867 10.1186/s12955-018-1001-0 PMC6127948

[112] Beaudequin D , Can AT , Jones M , et al. Relationships between reduction in symptoms and restoration of function and wellbeing: outcomes of the oral Ketamine trial on suicidality (OKTOS). Psychiat Res. 2021;305:114212. 10.1016/j.psychres.2021.114212 34563973

[113] Bahadır Ağce Z , Ekici G. Person-centred, occupation-based intervention program supported with problem-solving therapy for type 2 diabetes: a randomized controlled trial. Health Qual Life Outcomes. 2020;18(1):1–14. 32746841 10.1186/s12955-020-01521-x PMC7398232

[114] Freak-Poli RL , Wolfe R , Wong E , Peeters A. Change in well-being amongst participants in a four-month pedometer-based workplace health program. BMC Public Health. 2014;14(1):1–10. 25224301 10.1186/1471-2458-14-953 PMC4180736

[115] Kolind MI , Vinkler S , Kristensen T , Hansen SV , Christensen JR. Daily life coping—Helping stress-afflicted people manage everyday activities. Scand J Occup. 2023;30(2):170–181. 10.1080/11038128.2022.2072948 35575491

[116] Rees C , Craigie M , Slatyer S , et al. Mindful self-care and resiliency (MSCR): protocol for a pilot trial of a brief mindfulness intervention to promote occupational resilience in rural general practitioners. BMJ Open. 2018;8(6):e021027. 10.1136/bmjopen-2017-021027 PMC6042610 29961022

[117] Addley K , Burke C , McQuillan P. Impact of a direct access occupational physiotherapy treatment service. Occup Med. 2010;60(8):651–653. 10.1093/occmed/kqq160 20952558

[118] Stuber F , Seifried-Dübon T , Tsarouha E , et al. Feasibility, psychological outcomes and practical use of a stress-preventive leadership intervention in the workplace hospital: the results of a mixed-method phase-II study. BMJ Open. 2022;12(2):e049951. 10.1136/bmjopen-2021-049951 PMC8867373 35197332

[119] Sayed Ahmed HA , Mohamed SF , Elotla SF , Mostafa M , Shah J , Fouad AM. Psychometric properties of the Arabic version of the problem areas in diabetes scale in primary care. Front Public Health. 2022;10:843164. 35284366 10.3389/fpubh.2022.843164 PMC8913881

[120] Marchand A , Haines VY , Dextras-Gauthier J. Quantitative analysis of organizational culture in occupational health research: a theory-based validation in 30 workplaces of the organizational culture profile instrument. BMC Public Health. 2013;13(1):1–11. 23642223 10.1186/1471-2458-13-443 PMC3653730

[121] Wang C , Hua Y , Fu H , et al. Effects of a mutual recovery intervention on mental health in depressed elderly community-dwelling adults: a pilot study. BMC Public Health. 2017;17(1):1–10. 28049503 10.1186/s12889-016-3930-z PMC5209883

[122] Vang ML , Pihl-Thingvad J , Shevlin M. Identifying child protection workers at risk for secondary traumatization: a latent class analysis of the professional quality of life scale-5. J Trauma Stress. 2022;35(6):1608–1619. 35899686 10.1002/jts.22863 PMC10087244

[123] De Kock JH , Latham HA , Cowden RG , et al. Brief digital interventions to support the psychological well-being of NHS staff during the COVID-19 pandemic: 3-arm pilot randomized controlled trial. JMIR Mental Health. 2022;9(4):e34002. 35044927 10.2196/34002 PMC8982650

[124] Cooper K , Barton GC. An exploration of physical activity and wellbeing in university employees. Perspect Public Health. 2016;136(3):152–160. 26194136 10.1177/1757913915593103

[125] Feicht T , Wittmann M , Jose G , Mock A , Von Hirschhausen E , Esch T. Evaluation of a seven-week web-based happiness training to improve psychological well-being, reduce stress, and enhance mindfulness and flourishing: a randomized controlled occupational health study. Evid-BASED Compl Alt. 2013;2013:1–14. 10.1155/2013/676953 PMC3893772 24489588

[126] McCullough ME , Emmons RA , Tsang J-A. The grateful disposition: a conceptual and empirical topography. J Pers Soc Psychol. 2002;82(1):112–127. 11811629 10.1037//0022-3514.82.1.112

[127] Neff KD. The development and validation of a scale to measure self-compassion. Self Identity. 2003;2(3):223–250.

[128] Kemper KJ , Gascon G , Mahan JD. Two new scales for integrative medical education and research: confidence in providing calm, compassionate care scale (CCCS) and self-efficacy in providing non-drug therapies (SEND) to relieve common symptoms. Eur J Integr Med. 2015;7(4):389–395.

[129] Weiner MF , Martin-Cook K , Svetlik DA , Saine K , Foster B , Fontaine C. The quality of life in late-stage dementia (QUALID) scale. J Am Med Dir Assoc. 2000;1(3):114–116. 12818023

[130] Parker GB , Hyett MP. Measurement of well-being in the workplace: the development of the work well-being questionnaire. J Nerv Ment Dis. 2011;199(6):394–397. 21629018 10.1097/NMD.0b013e31821cd3b9

