# Supplementary material for: What is well-being? A scoping review of the conceptual and operational definitions of occupational well-being
Source: J Clin Transl Sci. 2023 Oct 16;7(1):e227. doi: 10.1017/cts.2023.648 (PMC10643923; doi:10.1017/cts.2023.648)
Supplement: Bautista et al. supplementary material [file S2059866123006489sup001.docx]

| **Table 1:** Well-being assessments used in the occupational health and well-being literature | | | | | | |
| --- | --- | --- | --- | --- | --- | --- |
| **Well-being Assessment Citation** | **Name of Assessment** | **Items and Rating** | **Reliability/ Validity** | **Samples/Population(s) from occupational health and well-being studies** | **Assessment limitations noted in the occupational health and well-being studies** | **Other well-being assessments used in combination** |
| Di Fabio & Kenny, 2016 [1] | Acceptance of Change Scale (ACS): subscales 1) Predisposition to change, 2) Support for change, 3) Change seeking, 4) Positive reaction to change, and 5) Cognitive flexibility | 20 items; rated 1-5 | α = 0.73 to 0.88 | 261 Italian workers of different public and private organizations in central Italy participated in the study. [2] | The research was conducted with a group of Italian workers who were not representative of the Italian population. [2] | Satisfaction with Life Scale [3]; PERMA The 10 Item Personality Inventory (TIPI) [4]; Workplace Relational Civility Scale (WRCS) [5] |
| Robinson et al., 1991 [6] | Afectometer 2 |  | Reported adequate test-retest reliability, internal consistency | 670 students in Jordan. [7] |  |  |
| Bradburn, 1969 [8] | Affect Balance Scale (ABS) | 10 items (5 for positive and 5 for negative) |  | 10308 civil servants (6895 men, 3413 women) aged 35-55 in London [9] | Primarily white, middle-aged men, [9] |  |
| Beck et al., 1993 [10] | Beck Anxiety Inventory (BAI) | 12 items; rated 0-3 | α = 0.93 | Participants were drawn from a larger multisite project examining psychosocial outcomes among youth with SCI and their primary caregivers.[11] |  | BD-II [12]; Brief COPE [13]; CDI [14]; PedsQL 4.0 Generic Core Scales [15] |
| Beck et al. 1979 [16];  Beck et al., 1961 [17] | Beck Depression Inventory (BDI) | 21 items; rated 0-3 | α = 0.85 | 211 individuals living in Poland, ages 66-80, who are Jewish and non-Jewish survivors of World War II (WWII). [18]; Early elementary school teachers [19] | Reliance on self-report, small, racially homogenous sample. [19] | State-Trait Anxiety Inventory [20] |
| Beck, Steer, & Brown, 1996 [12] | Beck Depression Inventory-II (BD-II) | 12 items; rated 0-3 | α = 0.92 | Participants were drawn from a larger multisite project examining psychosocial outcomes among youth with SCI and their primary caregivers. [11] |  | BAI [21]; Brief COPE [13]; CDI [14]; PedsQL 4.0 Generic Core Scales [15] |
| Reynolds & Kamphaus, 2004 [22] | Behavior Assessment System for Children, Parent Rating Scales, 2nd Ed (BASC-2 PRS) | The Parent Rating Scales (PRS) have between 134to 160 items using the same 4-point rating system. | α generally in the 0.90s for the composite scales, and reliabilities generally in the 0.80s for individual scales across all forms (TRS, PRS, SRP) in both the general sample and the clinical sample. | Thirty-one children with diagnosis of BPBI participated in this institutional review board–approved study. [23] | Inability to compare the child’s and parent’s assessment of self-concept and emotional-behavioral functioning utilizing their responses to the child and parent versions of the same standardized test. | Piers Harris Children's Self-Concept Scale, 2nd Ed (PHCSCS) [24] |
| Farmer & Sundberg, 1986 [25] | Boredom Proneness Scale (BPS) | 38 items; rated 1-7 | α = 0.81 | nurses working in both public (52.9%) and private (47.1%) health units in Greece. [26] | Cross-sectional design with self-report. [26] | Trait Emotional Intelligence Questionnaire-Short Form (TEIQue-SF) [27, 28] |
| Smith et al., 2008 [29] | Brief Resilience Scale | 6 items; 1=strongly disagree to 5=strongly agree | α = 0.88 | Australian ambulance officers (n=740). [30] | Cross-sectional, self-report, social desirability bias. [30] | Kessler 10 [31] |
| Derogatis, 1993 [32] | Brief Symptom Inventory (BSI) - Dutch version | Anxiety (6-items; 5-point Likert scale); Depression (6 items; 5-point Likert scale); Psychological distress (sum of two) (12 items; 5-point Likert scale) | Anxiety: α = 0.87; Depression: α = 0.87; Psychological distress (α = 0.93) | The study population consisted of Belgian emergency physicians (N=346), recruited at 2 national emergency medicine conferences. [33] | Under estimation of adverse consequence [33] | Dutch version of the Checklist Individual Strength [34]; Leiden Quality of Work Questionnaire for physicians (LQWQ-MD) [35]; Utrecht Work Engagement Scale (UWES) [36] |
| Glatzer & Gulyas, 2014 [37]  Levin & Currie, 2014 [38]  Cantril, 1965 [39]  Andrews & Robinson, 1991 [40] | Cantril ladder scale | 1 item; rated 0-10 | Not applicable to single-item scale; test-retest of binary measure Kappa = 0.44 - 0.54 | KOWEPS data. A total of 4,423 wage workers were divided into permanently employed workers, temporarily employed workers, and daily employed workers [41]; young people aged 15 to 17 years [42]; people with epilepsy in Norway. | Self-reports, cross-sectional, use of combined indexes—while aimed at simplifying the analyses—may have given rise to some interpretative difficulties [42] | Depression Scale for Children [43]; Perceived Stress Scale [44]; Cantril’s Ladder [38]; Scale of Perceived Social Self-Efficacy [45] |
| Radloff, 1977 [43]  Shrout & Yager, 1989 [46] | Center for Epidemiologic Studies Depression Scale (CES-D Scale) - Brief Version | 5 items; rated 0-3 | α = 0.65 | This study involves analysis of data from Phase II (begun in 1993) of the Massachusetts Elder Health Project, with older persons. The focus of Phase II was on the process of caregiving, from the perspectives of both caregiver and care recipient, with the objective of defining successful caregiving. [47] | The stressors were kept separate in the analyses. The study did not have sufficient sample sizes to look at the process separately for spouses versus children. [47] | University of Southern California Longitudinal Study of Three-Generation Families measures of positive affect [48] |
| Yang, et al.,  [49] | Center for Epidemiologic Studies Depression Scale (CES-D) - Chinese version | 20 items; 4-point Likert scale from 0 (rarely) to 3 (most or all of the time) | α = 0.872 | Doctors or nurses at two general hospitals in s in Liaoning, China. [50] | cross-sectional design, self-report questionnaires. [50] | Workplace Violence Scale [51]  Psychological Capital Questionnaire [52, 53] |
| Vercoulen et al., 1999 [34] | Checklist Individual Strength (CIS-20R); Perceived fatigue - Dutch version | 8 items; 7-point Likert scale | α = 0.93 | The study population consisted of Belgian emergency physicians (N=346), recruited at 2 national emergency medicine conferences. [33] | Cross-sectional; under estimation of adverse consequence, since the study recruited at 2 Belgian emergency medicine conferences, where the emergency physicians were really engaged in sharing their workplace experiences. [33] | Brief Symptom Inventory [32]; Leiden Quality of Work Questionnaire for physicians (LQWQ-MD) [35]; Utrecht Work Engagement Scale (UWES) [36] |
| Nyenhuis & Luchetta, 1998. [54] | Chicago Multiscale Depression Inventory (three subscales of depression: Mood, Evaluative (self-criticism), Vegetative (physical amlfunctioning) (CMDI) | 50 items; rated 1-5 Likert scale | α = 0.89 | 57 individuals aged 35-65 with clinically defined MS [55] | Limited age range, cross-sectional | MS Quality of Life [56] |
| Kovacs, 2003 [14] | Children’s Depression Inventory (CDI) | 27 items; rated 1-2 | α = 0.87 | Participants were drawn from a larger multisite project examining psychosocial outcomes among youth with SCI and their primary caregivers. [11] |  | BD-II [12]; Brief COPE [13, 57] |
| Ravens-Sieberer & Bullinger, 2000 [58] | Children’s Quality of Life Scale (KINDL) Revised | 24 items divided into six dimensions: physical well-being, emotional well-being, self-esteem, family, friends, and everyday functioning at school | α = 0.85 | Chronically ill children suffering from diabetes or asthma. [59] | Parents served as a proxy on behalf of the children to fill out the measure. [59] |  |
| Evans, C. 2002 [60] | Clinical Outcomes in Routine Evaluation – Outcome Measure (CORE-OM) | 34-item self-report instrument, domains of subjective well-being, symptoms, function, and risk. Five-point scale ranging from ‘not at all’ to ‘most or all the time’ | Internal and test–retest reliability (0.75–0.95), | Condition Management Program (CMP) to help Incapacity Benefit recipients manage their health conditions. 2064 participants with physical and mental health conditions participated. 50% had improvement in psychological well-being. [61] |  | Work and Social Adjustment Scale [62], Self-Efficacy Scale [63], Intrinsic Motivation Scale [64] |
| Feldman et al., 2007 [65] | Cognitive Affective Mindfulness Scale-Revised (attention, present-focus, awareness, acceptance/non-judgement domains) | 12 items; rated 1-4 (1=rarely/not at all to 4=almost always; items 2,6,7 are reverse scored) | α = 0.74 - 0.77 | First-year surgical and mixed-specialty resident trainees [66] | Small sample size, limited generalizability, did not account for informal practice [66] | Screen for high emotional exhaustion and high depersonalization [67] |
| Campbell-Sills & Stein, 2007 [68]; Connor & Davidson, 2003 [69] | Connor-Davidson Resilience Scale (CDRISC) | 10 items; rated 0-4 | α = 0.92 | Physicians and APPs employed by the Health Texas Provider Network (HTPN) [70] | Cross-sectional, self-selection bias, limited external validity, unmeasured covariate bias [70] | Interpersonal Reactivity Index Brock & Salinsky, 1993 [71]  Mayo Clinic Physician Well-being Index (MCPWBI) [72];  Psychological Empowerment Instrument [73, 74] |
| Connor & Davidson, 2003 [69]; Campbell-Sills & Stein, 2007 [68] | Connor–Davidson Resilience Scale (CD-RISC10) - French Canadian | 10-items; rated 0-4 | α = 0.86 | College Women [75] | The sample was restricted to young adult women attending college. Further validation of the scale is warranted with a sample involving all genders. [75] |  |
| Carver, 1997 [13];  Cooper et al. 2008 [57] | COPE (Brief) | 28 items; rated 1-4 | α = 0.72 for the emotion-focused scale, 0.84 for problem-focused scale, and 0.75 for the dysfunctional coping scale. | Participants were drawn from a larger multisite project examining psychosocial outcomes among youth with SCI and their primary caregivers. [11] |  | BD-II [12]; BAI [10]; Brief COPE [13, 57]; CDI [14]; PedsQL 4.0 Generic Core Scales [15] |
| Arsalani et al., 2011 [76] | Copenhagen Psychosocial Questionnaire – Farsi version | 95 items; with the exception of four 2-option items, the remaining items were in a 5-option format | α ≥ .61 | office and operational employees of one of the largest electricity distribution companies in Iran [77] | one electricity distribution company in Iran, thus generalization of results to other companies may not be appropriate [77] |  |
| Myers et al., 2020 [78]  Myers et al., 2017 [79]  Prilleltensky et al., 2015 [80] | COPPE action scale - Expanded version (interpersonal, community, occupational, physical, psychological, and economic) | 18 items total with each domain of well-being actions has 3 items; 7-point Likert scale that ranges from 0 (never) to 6 (always) | α = 0.87 (economic) to 0.93 (community) | Adults with obesity or overweight [78];  479 adult employees at a major university in the southeast of the USA were enrolled. [79] | Narrow population, modest sample size, data were not analyzed in a longitudinal framework, uncertainty regarding full participation of the subjects [79] |  |
| Lovibond & Lovibond, 1995 [81]  Henry & Crawford, 2005 [82]  Dreyer et al., 2019 [83]  Tran et al., 2013 [84]  Chan et al., 2012 [85]  Patrick et al., 2010 [86]  Almhdawi et al., 2018 [87] | Depression Anxiety Stress Scale (DASS); Dutch version of DASS; Serbian translation; English version; Chinese version | 21 items; rated 0-3 | α = 0.72 to 0.87 | In the Netherlands, invited 8000 people of insurance company A, and 2370 people of insurance company B to take part in the survey. [88]; Employees at a major tertiary healthcare institution (the Mayo Clinic). [89]; Surgeons and medical trainees in Malaysia (n=100). [90]; Allied healthcare professionals- social workers, psychologists, occupational therapists, physiotherapists, speech therapists in Australia (n=139). [91]; Hospital clinicans (doctors, nurses, and midwives) in Australia. [92]; Thirty inpatient psychiatric patients in Serbia participating in nature-based therapy program. [93]; Thirty-seven individuals with central visual field loss attending a vision rehabilitation clinic randomized to receive a visual magnifier or not. [94]; Adults with chronic SCI aged 70 years or less [95]; Chinese primary and middle school teachers teaching online during the COVID-19 outbreak [96]; University professors of all majors in Jordan [97] | Small sample, participants were self-selected, lack of blinding in the study/ every participant was aware of their allocation [95]; An online survey which could have limited generalizability [97] | Multidimensional Scale of Perceived Social Support (MSPSS) [98]; Overall Job Satisfaction Zimet et al., 1990 [99]; Self-Compassion Scale (SCS) [100]; WHO (Five) Well-Being Index (WHO-5) [101, 102]; Satisfaction with Life Scale[103]; Impact of Vision Impairment questionnaire [104]; Personal Well-being Index–Adult [105]; Spinal Cord Lesion Emotional Well-being Questionnaire version 1 Australia [95]; The International Physical Activity Questionnaire (IPAQ) [106-108] |
| Radloff, 1977 [43] | Depression Scale for Children | 10 items; 4-point Likert scale (rarely or never to all the time) | α = 0.84 - 0.85 | young people aged 15 to 17 years [42] | Self-reports, cross-sectional, use of combined indexes—while aimed at simplifying the analyses-may have given rise to some interpretative difficulties [42] | Perceived Stress Scale [44]; Cantril’s Ladder [38]; Scale of Perceived Social Self-Efficacy [45] |
| Schaufeli et al., 2009 [109]  Schaufeli et al., 2008 [110] | Dutch Work Addiction Scale -Brazilian version | 10 items; four-point Likert scale ranging from 1 (almost never) to 4 (almost always) | α = 0.80 | 1092 Brazilian employees [111] | Non-representative convenience sample [111] | Utrecht Working Engagement Scale [112] |
| Cox et al. 1987 [113] | Edinburgh Postnatal Depression Scale (EPDS) | 10 items; rated 0-3 | mothers: α = 0.87, fathers: α = 0.76 | Parents of preterm infants [114]; A sociodemographically diverse consecutive cohort of employed nulliparous women was recruited in late pregnancy [115] | The sample is more educated and likely to be employed in a professional occupation than the general Australian parturient population and therefore the findings cannot be generalized to women in lower waged, unskilled occupations [115] | EPDS [113]; STAI-STATE [20];  Profile of Mood States [116] |
| Li et al., 2005 [117] | Effort-reward imbalance questionnaire - Chinese version | Extrinsic effort 6 items; 5-point scale  Reward 11 items; 5-point scale  Over-commitment 6 items; rated 1-4 | Extrinsic effort α = 0.911  Reward α = 0.936  Over-commitment α = 0.771 | Northern region of China: 26 factories (1500 workers) were randomly selected by cluster sampling in an industry which made component parts of airplanes. [118] |  | Chinese version of the Flourishing Scale (FS) [119]; Chinese version of the Maslach Burnout Inventory-General Survey (MBI-GS)  [120-122];  Chinese version of the Psychological Capital Questionnaire (PCQ) [123];  Chinese version of the Rosenberg Self-Esteem Scale [124] |
| Maslach, et al., 1986 [125] | Emotional exhaustion scale | 9 items; rated 0-6 | None | US Army special operations soldiers [126] | Not generalizable to women and conventional soldiers. [126] | Role Overload Scale from the Michigan Organizational Assessment Questionnaire  [127]; Walter-Reed Army Institute of Research Soldier-Specific Functional Impairment Scale [128]; PSS [129] |
| Rabin & Frank, 2001 [130, 131] | EuroQOL | 16 states describing 216 possibilities | Spearman’s rho close to 1 | EuroQoL-5D (EQ-5D) was used to measure the following dimensions of well-being in Finish metal workers: 1) mobility, 2) self-care, 3) usual activities, 4) pain/discomfort, and 5) anxiety/depression. [132] |  |  |
| Bakker et al., 2004 [133] | Exhaustion domain of Oldenburg Burnout Inventory (OLBI-exhaustion) | 4 items; rated 1-4 | composite reliability (CR) = 0.743 | The study used secondary data retrieved from a one-time survey at multi-worksites that was conducted from November survey at multi-worksites that was conducted from November 2012 to May 2013 [134] |  | Three-Eating Factor Questionnaire (TEFQ-R21) [135] |
| Diener et al., 2010 [119];  Sumi, 2014 [136] | Flourishing Scale (FS) - Chinese version  Flourishing scale - Japanese version  Flourishing Scale - German version | 8 items; rated 1-7 | α = 0.950;  Adequate construct validity, and test-retest reliability of the Japanese version | Northern region of China: 26 factories (1500 workers) which made component parts of airplanes. [118]; Adults (>20 years old) workers at companies that often engage in desk work; specifically, a person who sits in front of a computer for at least half their work hours [137] |  | Chinese version of the effort-reward imbalance questionnaire [117]; Chinese version of the Maslach Burnout Inventory-General Survey (MBI-GS)  [120-122]; Chinese version of the Psychological Capital Questionnaire (PCQ) [123]; Positive and Negative Affect Schedule (Japanese version) [138]; Satisfaction with Life Scale [139] |
| Cox, 2011 [140] | Forgiveness Climate | 3 items; rated 1-5 | α = 0.83 | Nurses and doctors in the healthcare sector at two different times. [141] |  | Satisfaction with Life Scale  [139]; Workplace Inactivity [142] |
| Cella D, 1993 [143] | Functional Assessment of Cancer Therapy–General (FACT–G) | 7 Likert-scale questions over 4subscales address physical, emotional, social, and functional well-being | High test–retest reliability, r=0.92 for total scale r=.82 to .88 for subscales | 1:1 fatigue management course in 49 people with chronic conditions improved quality of life. [144] |  | Functional Assessment of Chronic Illness Therapy–Fatigue Scale (FACIT FS), Self-Efficacy for Performing Energy Conservation Strategies Assessment (SEPECSA) |
| Peterman et al, 2002 [145];  Lazenby et al, 2013 [146] | Functional Assessment of Chronic Illness Therapy-Spiritual Well-Being (meaning of life, peace, and the force linked to faith) - Arabic version | 12 items; rated 0-4 | α = 0.745 | 115 Lebanese cancer patients admitted to Hôtel-Dieu de France Hospital (HDF), Beirut—Lebano [147] | Convenience sampling, one university hospital, cross-sectional, spirituality and coping and their effect on quality of life are difficult concepts to study [147] |  |
| Chirico et al., 2020 [148] | General Health Questionnaire -30 item Italian version | 30 items; rated 0-3 | α = 0.92 | 50 teachers at a Catholic school in Italy. [148] |  |  |
| Goldberg and Williams, 1988 [149] | General Health Questionnaire-12 (GHQ-12) | 12 items; rated 0-3 | α = 0.87 | 196 employees in two different office layouts: one large open-plan office and several smaller office buildings with a more independent layout.[150]  Baseline data were collected from two regions in central Sweden from October 2019 to January 2020, and the longitudinal data 1 year later, from October 2020 to January 2021. [151] | Low response rate [152]; Small sample size and the RTW Beliefs Questionnaire is newly developed and some of its psychometric properties therefore remain unknown [151] | Need for Recovery [153]; Risk Factor Questionnaire [154] |
| Schwarzer & Jerusalem, 1995. [63] | General Self Efficacy Scale | 10 questions; 4 responses (not at all true, hardly true, moderately true, and exactly true) | α = 0.75 - 0.90 | 770 employees at a hospital in the south of Sweden. [155] | Cross-sectional, data from only one hospital [155] | Work Experience Measurement Scale [156]; Utrecht Work Engagement Scale [112]; Salutogenic Health Indicator Scale [157] |
| Yesavage et al., 1983 [158] | Geriatric Depression Scale (GDS) | 30 items; rated yes/no | α = 0.94 | 28 older adults (60 years or older) in Long Term Care facility [159]; 120 patients approximately 58 years old (SD = 8.6) after their first stroke [160] | Attrition (28% of participants dropped out) [159] | Life Satisfaction Index-A [161] |
| Goldberg et al., 1988 [149] | Goldberg Depression and Anxiety Scale | 18 items; rated 0-9 | The anxiety scale had a positive predictive value of 0.56; the depression scale had a positive predictive value of 0.86 | Child abuse investigators (police officers and staff) [162]; Employees at companies with option to telecommute. [163] |  | Impact of Events Scale-E [164]; Professional Quality of Life Scale [165]; Off-work hours technology-assisted job demand [166]; Toxic Leadership Scale [167]  Bergen Work Addiction Scale [168]; Siegrist Effort/Reward Imbalance model [169] |
| Tibblin et al., 1990 [170] | Goteborg Quality of Life Instrument (QoL): Well-Being Scale | 19 items; rated 1-7 | α = 0.72-0.89 | Middle-aged woman. [171]; This study investigates 108 male workers in Sweden. [172] |  |  |
| Schalock et al., 2008 [173] | Health-related quality-intellectual and developmental disabilities (HRQoL-IDD) - Social Well-Being, Healthy Decision-Making, Emotional Well-Being and Functional Well-Being subscales | 16 items; we adopted a 5-option format (e.g., 1 = Never to 5 = Always) with visual images of fluid-filled cups representing the range of responses | The HRQoL-IDD readability statistics indicated the measure is suitable for adults with low-literacy | Persons with intellectual and developmental disabilities. [174] |  |  |
| Hesbacher et al., 1980 [175]  Winokur et al, 1984 [176] | Hopkins Symptom Check List (SCL-8) | 8 items measuring psychological distress | correlation with the original 25 item version was 0.95 and reliability for the short version was estimated at 0.84 |  |  | Cantril Ladder [39, 40] |
| Zigmond & Snaith 1983 [177]  Bocerean & Dupret, 2014 [178] | Hospital Anxiety and Depression Scale | 14 items (7 for anxiety and 7 for depression); rated 0-3 | α = 0.71-0.91 | Members of the Pediatric Association of The Netherlands. French anesthesia and intensive care residents in years 1-5 of training. [179] | Low response rate, participation bias. [179] | Trauma Screening Questionnaire [180]; Pennebaker Inventory of Limbic Languidness (PILL) [181];  Copenhagen Burnout Inventory [182]; Perceived Stress Scale [44, 129] |
| Tehrani et al., 2002 [164] | Impact of Events Scale-E | 23 items; rated 1-5 | Intrusion: α = 0.86; Avoidance: α = 0.82 | Child abuse investigators (police officers and staff) [162] |  | Goldberg Depression and Anxiety Scale [149]; Professional Quality of Life Scale [165] |
| Lamoureux et al 2006 [104] | Impact of Vision Questionnaire | 32 items, subset for emotional well-being, 6 response categories | Able to discriminate differences in items between individuals with mild, moderate, severe visual impairment | 314 individuals referred to a low vision clinic |  | DASS-21 [81] |
| Morin et al., 2011 [183]  Sagherian et al., 2023 [184] | Insomnia Severity Index (ISI) | 3 items; rated 5-point Likert- type scale | α = 0.86-0.87 | Hospital nurses who provided bedside care in the United States. [185] | Convenience sampling, response bias, cross-sectional design. [185] | Occupational Fatigue and Exhaustion Recovery [186, 187]; Maslach Burnout Inventory-Human Services Survey [120]; Short Post-Traumatic Stress Disorder Rating Interview [188, 189]; Patient Health Questionnaire-4 [190, 191] |
| Citko et al, 2018 [106]  Powell et al., 2011 [107]  Helou et al., 2018 [108] | International Physical Activity Questionnaire (IPAQ) | Short version 9 items; long version 27 items; various rating scales; physical  activity level categorizing it into high, moderate, and low | Test-retest reliability = 0.46-0.96 | university professors of all majors in Jordan [97] | an online survey which could have limited generalizability [97] | Medical Outcomes Study Short Form (SF-12) [192, 193]  Depression Anxiety Stress Scale (DASS) [81, 86, 87]; Neck Disability Index (NDI) [194, 195]; The International Physical Activity Questionnaire (IPAQ) [106-108] |
| Brock & Salinsky, 1993 [71] | Interpersonal Reactivity Index (IRI): subscales 1) empathetic, and 2) perspective | 14 items; rated 1-5 | α = 0.81 to 0.84; | Physicians and APPs employed by the Health Texas Provider Network (HTPN) [70] | Cross-sectional, self-selection bias, limited external validity, unmeasured covariate bias [70] | Connor-Davidson Resilience Scale (CDRISC) [68, 69]  Mayo Clinic Physician Well-being Index (MCPWBI) [72]  Psychological Empowerment Instrument [73, 74] |
| Mohr et al., 2006 [196] | Irritation scale | 8 items; 7-point rating scale from 1 = ‘strongly disagree’  – 7 = ‘strongly agree | α = 0.80 - 0.89 | hospital staff, including nurses, physicians and other professions of  two German and three Latvian hospitals [197] | Selection bias by different cultural response styles, conclusions about causality is limited by cross-sectional.  data collection [197] |  |
| Hojat et al., 1999 [198] | Jefferson Scale of Attitudes toward Physician-Nurse Collaboration (JSAPNC) | 20 items; rated 1-4 | α = 0.81 | 522 physicians and nurses working in institutions of Mexico, Colombia, Ecuador, and Argentina. [199] | An unbalanced geographical distribution of the study sample (65% of the entire sample was composed by the Argentinean group, while the other 35% included data collected in ten Latin American countries). [199] | Jefferson Scale of Physicians Lifelong Learning (JeffSPLL) [200] |
| Alcorta-Garza et al., 2016 [201]  Hojat et al., 2002 [202]  Glaser et al., 2007 [203] | Jefferson Scale of Empathy  Jefferson Scale of Physician Empathy (Dutch version) | 20 items; rated 1-7 | α = 0.83 | Healthcare workers in a Spanish clinical context in the province of Lleida, Spain; multicenter questionnaire study and invited 271 surgery and gynecology residents, in 21 residency training programs, from two academic and 14 non-academic medical centers in the Netherlands. [204] | Self-selection participation bias. [204] | Social and Emotional Loneliness Scale for Adults [205-207]; Utrecht Work Engagement Scale [36, 208]; Physician Work life Study (Dutch version) [209, 210] |
| Muliira et al., 2012 [200] | Jefferson Scale of Physicians Lifelong Learning (JeffSPLL) | 14 items; rated 1-4 | α = 0.83 | 522 physicians and nurses working in institutions of Mexico, Colombia, Ecuador, and Argentina [199] | An unbalanced geographical distribution of the study sample (65% of the entire sample was composed by the Argentinean group, while the other 35% included data collected in ten Latin American countries). [199] | Jefferson Scale of Attitudes toward Physician-Nurse Collaboration (JSAPNC) [198] |
| Jenkins et al, 1988 [211] | Jenkin's Sleep Problem Scale | 4 items; rated 0-5 | α = 0.79 | Nationally representative sample of Finnish physicians [212] | Not generalizable to younger physicians or other countries [212] | Suicidal Ideation [59][60];  Work Ability Index, WAI [76] |
| Kessler & Mroczek, 1994 [31] | Kessler 10 (K10) | 10 items; (not at all) to 5 (very much) | α = 0.91 | Australian ambulance officers (n=740). [30] | Cross-sectional, self-report, social desirability bias. [30] | Brief Resilience Scale (Smith et al., 2008) |
| Ravens-Sieberer et al., 2008 [213] | KIDSSCREEN Survey for generic related health quality of life for children and adolescents | 52 items, 5-point likert-type scales to assess frequency or intensity of attitude | α = 0.77 - 0.89. | A representative sample of 22,827 children (8 to 18 yeas) in 13 European countries. 3,061 children in Denmark [214] | Limited by cross sectional data, limits in the identification of those with physical and mental status. [214] |  |
| Van der Doef & Maes, 1999 [35] | Leiden Quality of Work Questionnaire for physicians (LQWQ-MD) work–home interference Subscale | 4-items; 4-point Likert scale | α=0.84 | The study population consisted of Belgian emergency physicians (N=346), who were recruited at 2 national emergency medicine conferences. [33] | Cross-sectional; under estimation of adverse consequence, in view of the fact that the study recruited at 2 Belgian emergency medicine conferences, where the emergency physicians were really engaged in sharing their workplace experiences. [33] | Dutch version of the Checklist Individual Strength [34]; Brief Symptom Inventory [32]; Utrecht Work Engagement Scale (UWES) [36] |
| Fugl-Meyer et al., 2002[215]; Maujean & Devis, 2013[216] | Life Satisfaction Questionnaire (LiSat- 11) | 11 items; rated 1-6 | α = 0.86 | Women on sick leave due to long-term pain in the neck/shoulders and/or back. [217] | High missing data. [217] |  |
| Diener et al, 1985[103]  Di Fabio & Gori, 2016 [2] | Life Satisfaction Scale | 5 items; 1 (completely disagree) to 7 (completely agree) | α = 0.79 and 0.89 | Heterogeneous samples of workers from different organizations in Italy. [218]; Repeated cross-sectional study from three nationally representative surveys of 1997, 2005 and 2011 (n = 4485) in Colombia. [219]; 3,522 Korean University Students. [220] | Self-report measures, social desirability bias, cross-sectional design, convenience sample of works is not generalizable. [218] | Scale of Positive and Negative Experience [119] |
| Maslach et al., 1997 [221]  Pisanti et al, 2013 [222]  Carstensen et al., 2021  [223] | Maslach Burnout Inventory; Maslach Burnout Inventory (Italian version)  Maslach Burnout Inventory -German version | 22 items across 3 subscales; 7- point Likert type scale from 0 “never” to 6 “every day”; 9 items on emotional exhaustion subscale, 5 items on the depersonalization subscale, and 8 items on the personal accomplishment subscale  German version: 4 items only to assess emotional exhaustion. | α = 0.90 for emotional exhaustion; α = 0.79 for depersonalization; α = 0.71 for personal accomplishments | Arthroplasty surgeons [224]; 1749 doctors in Ireland [225] ; nurses at a public university hospital in the Terni—Umbria region of Italy. [226] hospital nurses who provided bedside care in the United States. [185]  German version of the MBI was used with 1758 beginning teachers and 831 prospective teachers and the reliability coefficient was .80.  About 150 caregivers in healthcare centers in United States. Reliability coefficient for emotional exhaustion (5-item version of the Maslach Burnout Inventory) was .88. This study used two other assessments of well-being: emotional thriving and emotional recovery. [227] | Potential that some participants reported a pre-existing musculoskeletal pain, underreporting related to the culture of physician resilience [224]; limited generalizability because all nurses working for the same organization and the majority were female. [226]; Convenience sampling, response bias, cross-sectional design. [185] | Insomnia Severity Index [183, 185]; Occupational Fatigue and Exhaustion Recovery [186, 187]; Short Post-Traumatic Stress Disorder Rating Interview [188] [189]; Patient Health Questionnaire-4 [190, 191] |
| Maslach & Jackson, 1981 [120];  Schaufeli, 1996 [121];  Li, 2002 [122] | Maslach Burnout Inventory-General Survey (MBI-GS) - Chinese version Subscales 1) emotional exhaustion, 2) cynicism, and 3) professional efficacy | 16 items; rated 0– 6. | α = 0.928 to 0.940 | Northern region of China: 26 factories (1500 workers) were randomly selected by cluster sampling in an industry which made component parts of airplanes. [118] |  | Chinese version of the Flourishing Scale (FS)  [119]; Chinese version of the Psychological Capital Questionnaire (PCQ) [123] |
| McManus et al., 2002  [228] | Maslach Burnout Inventory-Human Service Survey -Abbreviated (emotional exhaustion, depersonalization, and personal accomplishment) | 9 items; rates 0 -6 | Excellent fit (p=0·579), with a high goodness of fit index (0·996) and adjusted goodness of fit index (0·980) | First-year surgical and mixed-specialty resident trainees. [66] | Small sample size, limited generalizability, did not account for informal practice. [66] | Cognitive Affective Mindfulness Scale-Revised [65]; Abbreviated (9-item) 2-item screen for high emotional exhaustion and high depersonalization [67] |
| Dyrbye et al., 2013 [72] | Mayo Clinic Physician Well-being Index (MCPWBI) | 7 items; rated yes/no | α = 0.83 | Physicians and APPs employed by the Health Texas Provider Network (HTPN) [70] | Cross-sectional, self-selection bias, limited external validity, unmeasured covariate bias [70] | Connor-Davidson Resilience Scale (CDRISC) [68, 69];  Interpersonal Reactivity Index [71] |
| Dyrbye et al., 2010 [229] | Medical Students Well-Being Index (MSWBI) | 7 “yes or no” questions; rated 1 or 0. MSWBI scores range from 0 to 7, and 7 points indicate the greatest level of distress. | α = 0.68; The majority of MSWBI items had a ≥74% sensitivity and specificity for detecting distress within the intended domain. | Medical students of all years (1st–6th) from all 22 Polish medical schools. [230] |  |  |
| Keyes, 2005 [231]  Hone et al., 2014 [232] | Mental Health Continuum-Short Form (MHC-SF) | 14 items: emotional well-being (3 items), social functioning (5 items), and psychological functioning (6 items), 6-point scale, never to almost everyday | excellent internal consistency and discriminant validity | Population-based data from the 2012 Canadian Community Health Survey. [233] | Lack of data on personal or family history of mental health conditions. [233] |  |
| Clarke et al., 2014 | Mental Health Self-Efficacy Scale (MHSES) | 6 items; rated 1-10 | α = 0.89 | Participants in activity-based group therapy in an acute psychiatric ward in a large tertiary hospital in Singapore. [234] | Small sample size |  |
| Walach et al., 2006 [235] | Mindfulness questionnaire (FMI) | 14 items; rated 1-4 | α = 0.86 to 0.93 | Employees of two information and communication technology (ICT) companies [236] |  | Satisfaction with Life Scale [237]; Stress questionnaire (Stress) [238]; Utrecht Work Engagement Scale (UWES-9) [36, 112]; Work-Related Acceptance and Action Questionnaire (WAAQ) [239] |
| Baele & Fontaine, 2021 [240] | Moral-Distress Appraisal Scale | 8 items; rated 1-6 | convergent (r=0.09-0.43), predictive (r=0.41-0.63), and concurrent validity (r=0.22-0.52) | 406 Dutch-speaking healthcare professionals. [240] | Small sample, low scale reliability. [240] |  |
| Zimet, 1988 [98] | Multidimensional Scale of Perceived Social Support (MSPSS) | 12 items; rated 1-7 | α = 0.88 | In the Netherlands, invited 8000 people of insurance company A, and 2370 people of insurance company B to take part in the survey. [88] |  | Depression Anxiety Stress Scale (DASS); Dutch version of DASS [81, 82]; Overall Job Satisfaction [99] |
| Zweber et al., 2016 | Multi-faceted Organizational Health Climate Assessment (MOHCA) | 9 items, rated 1 to 7 | α =.91 | 531 full-time working adults (majority, White women) | Climate often conceptualized at work group level, but this scale is focused on individual level. Use of self- reported health measures rather than objective measures |  |
| Vickrey et al., 1995[56]  Nyenhuis & Luchetta 1998 [54] | Multiple Sclerosis Quality of Life (MSQOL) | 54-items | α = 0.70 | 231 definite MS patients  57 clinically diagnosed MS individuals. [55] | Severity of MS impacting physical functions |  |
| Vernon & Mior, 1991 [194]  Shaheen et al., 2013 [195] | Neck Disability Index – Arabic version | 10 items; 0 (no disability) to 5 (total disability) | α = 0.80 | university professors of all majors in Jordan [97] | An online survey which could have limited generalizability [97] | Medical Outcomes Study Short Form (SF-12) [192, 193]; Depression Anxiety Stress Scale (DASS) [81, 86, 87]; The International Physical Activity Questionnaire (IPAQ) [106-108] |
| van Veldhoven & Broersen, 2003 [241]  Sluiter et al., 2003 [153] | Need for recovery (NFR) Scale | 11 items; rated yes or no | α = 0.79 - 0.88 | Physicians of any grade registered with the UK General Medical Council or Irish Medical Council, and employed within a participating site, were eligible.[242]; 196 employees in two different office layouts: one large open-plan office and several smaller office buildings with a more independent layout. [152] | Low response rate. [152] | General Health Questionnaire-12 [149]; Risk Factor Questionnaire [154] |
| Winwood et al., 2005 [186]  Winwood et al., 2006 [187] | Occupational Fatigue and Exhaustion Recovery (OFER-15) | 15 items; 7-point Likert type scale from strongly disagree (0) to strongly agree (6) | α = 0.83-0.86 | hospital nurses who provided bedside care in the United States. [185] | Convenience sampling, response bias, cross-sectional design. [185] | Insomia Severity Index [183, 185]; Maslach Burnout Inventory-Human Services Survey [120];  Short Post-Traumatic Stress Disorder Rating Interview [188, 189];  Patient Health Questionnaire-4 [190, 191] |
| Demerouti et al., 2003 [243];  Demerouti et al., 2010 [244] | Oldenburg Burnout Inventory – Polish version (two aspects of burnout – exhaustion and disengagement) | 16 items; four-point scale ranging from 1 (Strongly agree) to 4 (Strongly disagree). | α = 0.79 | Polish police officers. [245] | Cross-sectional, may be an unrepresentative sample, more males than females. [245] | Utrecht Work Engagement Scale – Polish version [246] |
| Zimet et al., 1990 [99] | Overall Job Satisfaction | 1 item; rated 1-7 |  | In the Netherlands, invited 8000 people of insurance company A, and 2370 people of insurance company B to take part in the survey. [88] |  | Depression Anxiety Stress Scale (DASS); Dutch version of DASS [81, 82];  Multidimensional Scale of Perceived Social Support (MSPSS) [98] |
| Peto et al., 1995 [247] | Parkinson’s Disease Questionnaire (PDQ)-39 (eight discrete subscales: mobility, activities of daily living, emotional well-being, stigma, social support, cognition, communication, bodily discomfort) | 39 items; rated never to always | α = 0.69 - 0.94 | 394 active workers exposed to welding fumes. [248] | None of the PD specific QoL or health status questionnaires have been adequately validated for their specificity to PD. [248] |  |
| Kroenke et al., 2009 [190]  Löwe et al., 2010 [191] | Patient Health Questionnaire-4 | 2 items for depression and 2 items for anxiety; 4- point Likert type scale from not at all (0) to nearly every day (3) | α = 0.87-0.88 | hospital nurses who provided bedside care in the United States. [185] |  | Insomnia Severity Index [183, 185]; Occupational Fatigue and Exhaustion Recovery [186, 187]; Maslach Burnout Inventory-Human Services Survey [120]; Short Post-Traumatic Stress Disorder Rating Interview [188, 189] |
| Kansiewicz et al., 2022 [249] | Patient Health Questionnaire-9 | 10 items, scores 0-27 | α =0.89, excellent test-retest reliability | Assemblies of God ministers in the US.  1161 clergy in UMC in the US who were already in randomized trial measuring effects of lifestyle interventions on physical, emotional and mental health. [250] | Specific population weighted toward White, married males | Hospital Anxiety and Depression Scale [177]; Maslach Burnout Inventory [120] |
| Smith & Betz, 2000 [45] | Perceived Self-Efficacy | Eight positively oriented questions; Five categories of answers from not at all to very well | α = 0.841 | Young people aged 15 to 17 years. [42] | Self-reports, cross-sectional, use of combined indexes—while aimed at simplifying the analyses—may have given rise to some interpretative difficulties. [42] | Depression Scale for Children [43]; Perceived Stress Scale [44]; Cantril’s Ladder [38]; Scale of Perceived Social Self-Efficacy [45] |
| Cohen, 1983 [33] | Perceived Stress Scale; Perceived Stress Scale - 10 | 14 items, rate 0-4  10 items; 5-point scale | α = 0.84-0.90  test-retest reliability= 0.85 | Final year medical students from University College Dublin (UCD). [251]; Young people aged 15 to 17 years. [42]  52 Female staff that work at least 20 hours at York University. [252]; Data were collected through the “Moms As People” Survey, an online questionnaire developed to examine how mothers feel about various aspects of their lives, with the intention to oversample for relatively well-educated women. A total of 2,247 American women completed the survey between 2005 and 2010. [253] | The study is limited by the absence of a control group. [251]; Self-reports, cross-sectional, use of combined indexes—while aimed at simplifying the analyses—may have given rise to some interpretative difficulties. [42] | Depression Scale for Children [43]; Perceived Stress Scale [44]; Cantril’s Ladder [38]; Scale of Perceived Social Self-Efficacy [45] |
| Butler & Kern, 2016 [254] | PERMA scale (multidimensional model of well-being) | 5 dimensions: Positive Emotion (P), Engagement (E), Relationships (R), Meaning (M),Accomplishment (A). 23 items, 11-point Likert scale (0-10) used Japanese version | α =.75-0.96 | 310 workers completed questionnaire online. [255] |  | Brief Job Stress Questionnaire (BJSQ) [256], Utrecht Work Engagement Scale [257] |
| Trockel et al., 2018 [258]  Hamidi et al., 2018 [259]  Brady et al., 2022 [260] | Personal fulfillment Index | 4-item professional fulfillment and 2 dimensions of burnout: 4-item work exhaustion; 6-item interpersonal disengagement; 5-point Likert scale “not at all true” to “completely true” for professional fulfillment items and “not at all” to “extremely” for work exhaustion and interpersonal disengagement items | Test-retest reliability estimates were 0.82 for professional fulfillment (α = 0.91), 0.80 for work exhaustion (α = 0.86), 0.71 for interpersonal disengagement (α = 0.92), and 0.80 for overall burnout (α = 0.92). | Clinical faculty at the Stanford University School of Medicine.[261] | Used a binary gender classification, small number of non-White respondents precluded analysis of ethnicity, limiting the generalizability. [261] |  |
| Cummins, 2004 [105] | Personal Well-being Index | 8 items; rated 0-10. | α = 0.70- 0.85. | 1190 hospital midwives from 7 countries (2 Asian, 5 Europe). [262] |  |  |
| Lau et al., 2005 [263]  Lau et al., 2008 [264]  Cummins et al., 2004 [105]  Cummins et al., 2003 [265] | Personal well-being index – Chinese version and English version | 7 items; 0 (completely dissatisfied)–10 (completely satisfied); normative values range between 60 and 70 | α = 0.73 – 0.85 | Community dwelling older persons with mild dementia in Hong Kong.[266]; Adults with chronic SCI aged 70 years or less [95]; The NZAVS is an ongoing 20-year national longitudinal panel study of social attitudes, personality, and health outcomes that began in 2009. Thes ample analyzed in the current study involve participants who completed the survey during the nationwide Level 4 lockdown (March 25 through April27, 2020) as well as around the same time period the prior year in the tenth wave of the study. [267] | Small sample size, lack of community resources in dementia care, low minimum requirements for caregiver visits, low literacy level in spouse caregivers, and the non- compliance of participants with dementia, the program might also have inadvertently increased the burden to the family caregiver. [266]  Small sample, participants were self-selected, lack of blinding in the study/ every participant was aware of their allocation [95] | The Depression, Anxiety and Stress Scale short version [81]; Spinal Cord Lesion Emotional Well-being Questionnaire version 1 Australia [268] |
| Lawton, 1975 [269] | Philadelphia Geriatric Center Morale Scale – Japanese version (agitation, attitude toward own aging, lonely dissatisfaction) | 17 items; each high morale response=1, each low morale response=0 (the highest possible score is 17 points) | agitation α = 0,85  attitude toward own aging α = 0.81  lonely dissatisfaction α = 0.85 | aged home-based rehabilitation users with different levels of independence in activities of daily living. [270] | Small sample, limited generalizability, subjective well-being only represented one aspect of quality of life. [270] |  |
| Williams et al., 1999 [209]  Konrad et al., 1999 [210] | Physician Worklife Scale (job and specialty satisfaction measures; Dutch version) | 5 items in job satisfaction;3 items in the specialty satisfaction; 5-point Likert scale from 1 (strongly disagree) to 5 (strongly agree) | Job satisfaction α = 0.82; specialty satisfaction α = 0.80 | multicenter questionnaire study and invited 271 surgery and gynecology residents, in 21 residency training programs, from two academic and 14 non-academic medical centers in the Netherlands [199] |  | Utrecht Work Engagement Scale [36, 208];  Jefferson Scale of Physician Empathy (Dutch version) [202] |
| Sato, 2001 [138] | Positive and Negative Affect Schedule (Japanese version) | 20 items; rated 1-5 | α >0.80 and test-retest correlations >0.50 | Adults (>20 years old) workers at companies that often engage in desk work; specifically, a person who sits in front of a computer for at least half their work hours. [137] | Self-selection participation bias, low adherence. [137] | Flourishing scale (Japanese version) [136]; Satisfaction with Life Scale [139] |
| Watson, 1988 [28] | Positive and Negative Affect Schedule (PANAS) - Japanese version | 36 items – 18 (positive) and 18 (negative); rated 1-5 | α = 0.88 - 0.95 (positive), α = 0.87 - 0.92 (negative) | Japanese workers. [271]  Episcopal priests. [272] | Majority women | Satisfaction with life scale (SWLS) [139]; Personal Well-being Index [105] |
| Eid & Larsen RJ, 2008 [273] | Positive and Negative Affect Scale | 20 items; rated 1-5  10 items; rated 1-5 | High internal consistency and acceptable test–retest reliability | mothers of children with autism spectrum disorder in Taiwan. [274]; Family caregivers from Toronto and London Ontario and Montreal Quebec of individuals surviving their first stroke. [275] | Small sample. [274]; Survivors had mild-to-moderate stroke severity. [275] | Satisfaction with Life Scale [139] |
| Conner & Davidson, 2001 [188]  Davidson & Colket, 1997 [189] | Post-Traumatic Stress Disorder Rating Interview - Short | 8 items; 5-point rating scale from not at all (0) to very much (4) | α = 0.89-0.91 | Hospital nurses who provided bedside care in the United States [185] | Convenience sampling, response bias, cross-sectional design [185] | Insomnia Severity Index [183, 185]; Occupational Fatigue and Exhaustion Recovery [186, 187]; Maslach Burnout Inventory-Human Services Survey [120]; Patient Health Questionnaire-4 [190, 191] |
| Stamm, 2010 [165] | Professional Quality of Life Scale | 30 items; rated 1-5 | α = 0.75 – 0.81 | child abuse investigators (police officers and staff). [162]; 20 oncology nurses at a hematology-oncology unit in a large urban teaching hospital in Pennsylvania. [276] | Homogenous sample with no male nurses. [276] | Goldberg Depression and Anxiety Scale [149]; Impact of Events Scale-E [164] |
| Grove & Prapavessis, 1992 [277]  Berger & Motl, 2000 [278]  McNair et al., 1971 [116] | Profile of Mood States (POMS) with 7 subscales, tension, depression, fatigue, vigor, confusion, anger, and esteem-related affect | 40 items, 0 to 4, not at all to extremely | α = 0.664 to 0.954 | 24 participants with chronic low back pain and desk jobs. [279]  A sociodemographically diverse consecutive cohort of employed nulliparous women was recruited in late pregnancy. [115] | Small sample size, lack of diversity within the sample, excluded participants with low back pain but did not have a compatible desk. [279]; The sample is more highly educated and likely to be employed in a professional occupation than the general Australian parturient population and therefore the findings cannot be generalized to women in lower waged, unskilled occupations. [115] | Edinburgh Depression Scale [113] |
| Luthans et al., 2007 [123] | Psychological Capital Questionnaire (PCQ) - Chinese version | 24 items; rated 1-6 | α = 0.953 | Northern region of China: 26 factories (1500 workers) were randomly selected by cluster sampling in an industry which made component parts of airplanes. [118] |  | Chinese version of the Flourishing Scale (FS) [119]; Chinese version of the Maslach Burnout Inventory-General Survey (MBI-GS)  [120-122] |
| Li et al., 2015 [52]  Peng et al., 2013 [53] | Psychological Capital Questionnaire-Chinese version | 24-items (four core elements, including resilience optimism self-efficacy and hope); 6-point Likert scale from 1 (strongly disagree) to 6 (strongly agree) | α = 0.943 | Doctors or nurses at two general hospitals in s in Liaoning, China [50] | Cross-sectional design, self-report questionnaires [50] | The center for Epidemiologic Studies Depression Scale [43, 49]; Workplace Violence Scale [51] |
| Spreitzer, 1995 [73] Spreitzer, 2008: 54-76 [74] | Psychological Empowerment Instrument (PEI): Subscales 1) meaning, 2) Work meaningfulness, 3) Self-determination, and 4) Impacts | 3 items; rated 1-7 | α = 0.85 to 0.94 | Physicians and APPs employed by the Health Texas Provider Network (HTPN) [70] | Cross-sectional, self-selection bias, limited external validity, unmeasured covariate bias [70] | Connor-Davidson Resilience Scale (CDRISC) [68, 69]; Interpersonal Reactivity [71];  Mayo Clinic Physician Well-being Index (MCPWBI) [72] |
| Chassany et al., 2004 [280] Dagenais-Desmarais & Savoie, 2012 [281] | Psychological General Well-Being Index (PGWBI): Subscales: Interpersonal Fit at Work, Perceived Recognition at Work, Thriving at Work, Involvement at Work, and Feeling of Competency at Work | 22 items; rated 0-5 | α = 0.70 to 0.85 | Online survey, in nine tertiary educational institutions, eight Universities in Australia and one in New Zealand. [282]; Four public institutions in Gabon (Africa). [283] | Small sample. [282] | Short Almost Perfect Scale (SAPS) [284] |
| Yi et al., 2021 [285] | Psychological need thwarting scale of online teaching (autonomy, competence, and relatedness thwarting subscales) - Chinese version | 9 items; 7-point Likert-type scale (ranging from 1 to 7) | α = autonomy (0.78), competence (0.84), and relatedness (0.88) | Chinese primary and middle school teachers teaching online during the COVID-19 outbreak. [96] |  |  |
| Widerszal-Bazyl & Cieslak, 2000 [286] | Psychosocial Working Conditions Questionnaire, measures 5 theoretical scales: demands, controls, social support, well-being, and desired changes | Demands has 25 questions, controls has 20, social support 16, well-being 22, and desired changes 20; rated 1-5 | α = 0.82 - 0.94 | Professionally active registered nurses from an eastern voivodeship in Poland. [287] | Findings may not apply to other regions in Poland. [287] |  |
| Weiner et al., 2000 [288] | Quality of Life in Dementia Scale (QUALID) | 11 items; rated 1-5 | α = 0.74 | Residents in nursing homes. [289] |  | WHO (Five) Well-Being Index (WHO-5) [101] |
| Burckhardt et al., 1992 [290] | Quality of Life Scale – Swedish version | 16 items; 7-point satisfaction scale | α = 0.89 - 0.92 | Women with fibromyalgia. [291] |  |  |
| Kaplan et al 1997 [292];  Kaplan & Anderson, 1998 [293] | Quality of Well-being, Self-Administered (QWB-SA) | It has 5 scales and 58 questions using a dichotomous scale.  The Quality of Well-being scale is a preference-weighted measure combining three scales of functioning with a measure of symptoms and problems to produce a point-in-time expression of well-being that runs from 0 (for death) to 1.0 (for asymptomatic full function). |  | 218 English speaking adults who attended primary care clinics. 86 participants with combat-related major lower-limb amputation. [294]; 75 staff in ambulatory care environment in United States. [295] |  |  |
| Hays et al., 1993 [296] | RAND 36-Item Health Survey | 36 items, measured from 0 (poor) to 100 (good health) depending on subscale | α = 0.78- 0.93 | 288 independent living adults aged 65 years or over with normal cognition from UK. [297] |  | Physical health dimensions of the SF-36, extent of depression (PHQ-9) [298]; Quality of life (EQ-5D) scale [299]; Loneliness Scale [300] |
| Hays & Morales, 2001 [301] | RAND Emotional Well-being scale | 5 items, sores 0-100 |  | 40–60-year-old employees in Finland part of Helsinki Health Study. [302] |  |  |
| Hays & Morales, 2001 [301] | RAND Health Related Quality of Life (SF-36) | 36 item scale; ratings vary | α = 0.90 | Office workers. [303] |  | Sedentary Behavior Questionnaire [304] |
| Cheng & Hamid, 1995 [124] | Rosenberg Self-Esteem Scale -Chinese version | 10 items; 4-point Likert scale (strongly agree to strongly disagree) | α = 0.879 | Northern region of China: 26 factories (1500 workers) were randomly selected by cluster sampling in an industry which made component parts of airplanes. [118] |  | Chinese version of the Flourishing Scale (FS) [119];  Chinese version of the Maslach Burnout Inventory-General Survey (MBI-GS)  [120-122]; Chinese version of the Psychological Capital Questionnaire (PCQ) [123] |
| Eltayeb et al., 2007 [154] | Risk Factor Questionnaire | 12 items; 10 different body regions plus eyestrain and headaches rated yes-no, then summed | α = 0.90 | 196 employees in two different office layouts: one large open-plan office and several smaller office buildings with a more independent layout. [152] | Low response rate. [152] | General Health Questionnaire-12 [149]; Need for Recovery [153] |
| Cammann et al., 1979 [127] | Role Overload Scale from the Michigan Organizational Assessment Questionnaire | 4 items; rated 1-7 |  | US Army special operations soldiers. [126] | findings may not be generalizable to women and conventional soldiers. [126] | Emotional exhaustion scale [125]; Walter-Reed Army Institute of Research Soldier-Specific Functional Impairment Scale [128] |
| Ryff, 1989 [305];  Palma-Candia et al., 2019 [306]; Toledano-Gonzales et al., 2019 [307]; Jang et al., 2019 [308]; Sirigatti et al., 2009 [309] | Ryff Psychological Well-Being Scale; Ryff PWB (Spanish Version); Ryff PWB (Korean Version)  Ryff PWB (Italian Version) | 42 items; rated 1-7  6 subscales: self-acceptance, positive relations with others, autonomy, environmental mastery, purpose in life, and personal growth.  Spanish Version: 39 items; rated 1-7. Korean Version: 46 items; rated 1-7 | α = 0.86-0.93  α = .78-.81 (Spanish Version)  α = .66-.76 (Korean Version) | Older Adults in Magallanes, Chile. [306]; 74 older adults living in retirement homes. [307]; 399 Korean Nurses in a university hospital.  1142 people who report having multiple sclerosis. [310]; 2102 community residents in Tokyo aged 30. | Not possible to obtain two groups of similar sociodemographic characteristics. [306]; Small sample size. [307]; Only one hospital, and only for nurses; Self-reported, cross-sectional. [310] | Satisfaction with Life Scale [139] |
| Bringsen et al., 2009 [157] | Salutogenic Health Indicator Scale | 12 aspects of health; rated using a six-step semantic differential (positive to negative) | α > 0.70 | 770 employees at a hospital in the south of Sweden [155] | Cross-sectional, data from only one hospital [155] | Work Experience Measurement Scale [156];  Utrecht Work Engagement Scale [133] General Self Efficacy Scale [63] |
| Diener et al., 1985 [139]; Di Fabio & Gori, 2016 [3]; Pavot et al., 2008 [237]; Piotrkowska et al., 2019 [311]; Diener et al. 1985[139] | Satisfaction with Life Scale | 5 items; rated 1-7 | α = 0.61-0.91 | Mothers of children with autism spectrum disorder in Taiwan. [274]; adult (>20 years old) workers at companies that often engage in desk work; specifically, a person who sits in front of a computer for at least half their work hours. [137]; Two hundred and sixty-one Italian workers of different public and private organizations in central Italy participated in the study [2]; Nurses and doctors in the healthcare sector at two different times [141]; employees of two information and communication technology (ICT) companies [236]; Pharmacy students from five South Korean universities. [312]; Polish oncology nurses Australian Actors and performing artists. [313]; Older adults living with chronic conditions (living in Vancouver). [314]; 1142 people who report having multiple sclerosis. [310]; Data were collected through the “Moms As People” Survey, an online questionnaire developed to examine how mothers feel about various aspects of their lives, with the intention to oversample for relatively well-educated women. A total of 2,247 American women completed the survey between 2005 and 2010 [253]; 41 participants with non-traumatic spinal cord dysfunction and traumatic spinal cord injury in Australia. [315] | Small sample [274]; Self-selection participation, low adherence. [137]; The research was conducted with a group of Italian workers who were not representative of the Italian population [2]; Generalizability limited to second and third-year PharmD students from pharmacy schools in large cities. [312]; Group attending conference may not be representative of all oncology nurses. Self-reported, cross-sectional. [310] | Positive and Negative Affect Scale [273]; Mindfulness questionnaire (FMI) [235]; Stress questionnaire [238]; Utrecht Work Engagement Scale (UWES-9) [36, 112]; Work-Related Acceptance and Action Questionnaire (WAAQ) [239]  Acceptance of Change Scale (ACS) [1]; The 10 Item Personality Inventory (TIPI) [4]; Workplace Relational Civility Scale (WRCS) [5];  Forgiveness Climate Cox, 2011 [140]; Workplace Inactivity [142]; Flourishing scale (Japanese version) [136]  Positive and Negative Affect Schedule (Japanese version) [138]; Depression Anxiety Stress Scales (DASS-21) [81];  Ryff Psychological Well-Being Measure [305]; PSS-10 [44] |
| Diener et al., 2010 [119] | Scale of positive and negative experience | 12 items (6 devoted to positive and 6 devoted to negative experiences); 1 (very rarely or never) to 5 (very often or always) | Positive α = 0.87; negative α = 0.81 | Heterogeneous samples of workers from different organizations in Italy. [218] | Self-report measures, social desirability bias, cross-sectional design, convenience sample of works is not generalizable. [218] | Life Satisfaction Scale [3, 103] |
| West et al, 2012 [67] | Screen for high emotional exhaustion and high depersonalization | 2 items; rated 0 – 6 | Single-item measures of emotional exhaustion and  depersonalization exhibited strong associations with the full Maslach.  Burnout Inventory (all p≤0.008) | First-year surgical and mixed-specialty resident trainees. [66] | Small sample size, limited generalizability, did not account for informal practice. [66] | Cognitive Affective Mindfulness Scale-Revised [65]; abbreviated (9-item) Maslach Burnout Inventory-Human Service Survey [228] |
| Rosenberg et al., 2010 [304] | Sedentary Behavior Questionnaire: subscales 1) weekday, and 2) weekend | 9 items; rated by time spent in activities | ICC = 0.85 weekday and 0. 77 for weekend total scores; ICC range = 0.64 to 0.90 weekdays; 0.48-0.93 for weekend days | Office workers. [303] |  | RAND Health Related Quality of Life Hays & Morales, 2001 [301] |
| Neff, 2003 [100] | Self-Compassion Scale (SCS) | 26 items; rated 1-5 | α = 0.95 | Employees at a major tertiary healthcare institution (the Mayo Clinic). [89] |  | Depression Anxiety Stress Scale (DASS) [81]; WHO (Five) Well-Being Index (WHO-5) [101, 102] |
| Rosenberg, 2015. [316] | Self-Esteem Scale | 10 items; 4-point Likert scale ranging from strongly agree to strongly disagree | α = 0.88 | Pharmacy students from five South Korean universities. [312] | Generalizability limited to second and third year PharmD students from pharmacy schools in large cities. [312] | Satisfaction with Life Scale [139] |
| Linn & Linn, 1984 [317] | Self-Evaluation of Life Function Scale (physical health, symptoms of aging, self-esteem, social satisfaction, depression, personal control) | 52 items; 1-4 | Test-retest reliability = 0.36-0.99 | African American adults with sickle cell disease. [318] | Small sample, length of the survey and types of questions may have discouraged respondents from completing the survey, respondents’ tendency to rate themselves more favorability, scale was standardized on the older population and mean age in this study was 43 years, not generalizable. [318] |  |
| Ware and Gandek, 1998 | SF-36 Health Survey, a 36-item generic measure of health status | 36 items that fit into relevant health domains scale is not consistent throughout | α = 0.68 - 0.93 | U.S. General Population Data 41 participants with non-traumatic spinal cord dysfunction and traumatic spinal cord injury in Australia. [315] |  |  |
| Rice, 2014 [284] | Short Almost Perfect Scale (SAPS) | 8 items; rated 1-7 | α = 0.84 - 0.90 | Online survey, in nine tertiary educational institutions, eight Universities in Australia and one in New Zealand. [282] |  | Psychological General Well-Being Index (PGWBI) [280] |
| Ware et al., 1996 [192]  Al Sayah et al., 2013 [193]  Pickard et al., 1999 [319] | Short Form 12 (SF-12; produced physical component score for physical well-being and mental component score for mental well-being) - Italian version or the Medical Outcomes Study Short Form (SF-12) | 12 items; some rated yes/no, others rated excellent to poor, not at all to extremely | SF-12 achieved a R^2^ of 0.911 in the prediction of PCS-36 and 0.918 in the prediction of MCS-36 | Nurses, physicians, residents, and medical and nursing students in the oncology-hematology units of 3 teaching hospitals in Rome. [320];  1871 Australian veterans who served in the Gulf region during the period August 2, 1990, to September 4, 1991. [321]  University professors of all majors in Jordan. [97]  254 individuals who met criteria for mild stroke at Washington University | Cross-sectional and self-report. [320]  Only included probable musculo skeletal conditions, these were not confirmed by a diagnostic process or a validity study. [321]; An online survey which could have limited generalizability. [97] | Depression Anxiety Stress Scale (DASS) [81, 86, 87]  Neck Disability Index (NDI) [194, 195];  The International Physical Activity Questionnaire (IPAQ) [106-108] |
| DiTommaso et al., 2004[205]; Domínguez et al., 2017[206]; Marilaf-Caro et al., 2017[207] | Social and Emotional Loneliness Scale for Adults | 15 items; rated 1-7 | α = 0.88, good concurrent and discriminant validity | Healthcare workers in a Spanish clinical context in the province of Lleida, Spain. [204] | Self-selection participation bias | Jefferson Scale of Empathy [201] |
| Hunt, McEwen & McKenna, 1985 | Social Isolation Subscale of the Nottingham Health Profile (NHP) | 5 items; rated 0-5 | α = 0.67 | 211 individuals living in Poland, ages 66-80, who are Jewish and non-Jewish survivors of World War II [18] |  | BDI [16] |
| Gresham & Elliott, 1990 [322] | Social Skills Rating System (SSRS) | Academic subscale (10 items);  Problem Behavior Scale (6-items); internalizing subscale (9-items); 5-point scale | α = 0.61-0.81 parent-reported subscales.  α = 0.78 - 0.92 teacher-reported subscales | New Hope Project eligibility sent to all low-income residents of two neighborhoods in Milwaukee’s poorest areas. [323] |  |  |
| Wang et al., 2017 [324] | Special Education Teacher's Occupational Well-being Questionnaire (SETOWQ) | 25 items; rated 1-5 | α = 0.93 | Chinese Special Education Teachers. [325] | Study’s participants were mainly sampled from the east coast of China; not generalizable to special education teachers across the whole country. [325] |  |
| Migliorini et al., 2008 [268] | Spinal Cord Lesion Emotional Well-being Questionnaire version 1 Australia (three domains: helplessness, intrusion and personal growth) | 12 items; rated 1 = strongly disagree, 2 = disagree, 3 = agree or 4 = strongly agree | α = 0.78 | Adults with chronic SCI aged 70 years or less. [95] | Small sample, participants were self-selected, lack of blinding in the study/ every participant was aware of their allocation. [95] | The Depression, Anxiety and Stress Scale short version [81]; Personal Well-being Index–Adult [105] |
| Paloutzian & Ellison 1991 [326] | Spiritual Well-Being Scale | 20-item Likert scale; two subscales: religious well-being (RWB) and existential well-being (EWB).  sum of two subscales is total spiritual well-being score, with higher total score indicating higher spiritual well-being. | α =.0.87 | Relations of spiritual well-being, global job satisfaction, and general self-efficacy to hope in 64 Continuing Care Assistants. [327] |  |  |
| Malinakova et al., 2017 [328] | Spiritual Well-Being Scale (SWBS)-Shortened version | 7 items, 6-point Likert Scale ranging from strongly agree to strongly disagree | α =0.814 | A nationally representative sample (4217) of Czech adolescents [328]; 4182 Czech adolescents [329] | self-report, problems with some of the items on the scale which created a separate factor |  |
| Spielberger,1999 [330] | State-Trait Anger Expression Inventory (STAXI2-State) | 15 items; rated 1-4 | mothers: α = 0.92, fathers: α = 0.87 | Parents of preterm infants [114] |  | EPDS [113]; STAI-State [20] |
| Spielberger et al. 1983 [20];  Kvaal et al., 2005 [331] | State–Trait Anxiety Inventory (STAI-State) | 20 items; rated 1-4 | mothers: α = 0.94, fathers: α = 0.93 | Parents of preterm infants [114]; Early elementary school teachers. [19]; Teachers’ perspectives from a German medical faculty during COVID-19. [332] | Reliance on self-report, small, racially homogenous sample. [19]; Teachers from only one medical facility, and the numbers of participants was limited to 24. [332] | EPDS [113]; STAXI2-State [330]; Beck Depression Inventory [17] |
| Elo et al., 2000 [238] | Stress questionnaire | 1 item; rated 1-5 |  | Employees of two information and communication technology (ICT) companies. [236] |  | Mindfulness questionnaire (FMI) Walach et al., 2006;  Satisfaction with Life Scale Pavot et al 2008; Utrecht Work Engagement Scale (UWES-9) [36, 112]  Work-Related Acceptance and Action Questionnaire (WAAQ) [239] |
| Kessler et al., 1999 [333]; Lindfors et al., 2009 [334] | Suicidal Ideation | 1 item; Yes/No: ever had a suicidal thought or attempt. | Found to predict suicide attempts | Nationally representative sample of Finnish physicians [212] | Not generalizable to younger physicians or medical specialists from other countries [212] | Jenkin's Sleep Problem Scale [211]; Work Ability Index, WAI [76] |
| Gosling et al., 2003 [4] | Ten Item Personality Inventory (TIPI): Subscales 1) Extraversion, 2) Agreeableness, 3) Conscientiousness, 4) Neuroticism, and 5) Openness | 10 items; rated 1-7 | α = 0.82 | Two hundred and sixty-one Italian workers of different public and private organizations in central Italy participated in the study.[2] | The research was conducted with a group of Italian workers who were not representative of the Italian population.[2] | Acceptance of Change Scale (ACS) [1]; Satisfaction with Life Scale [3]; Workplace Relational Civility Scale (WRCS) [5] |
| Cohen & Williamson (1988) | The Medical Outcomes Questionnaires Short Form-12 (SF-12) | 12 Items; rated 0-100 | Physical component summary (PCS): α = 0.89; mental component summary (MSC): α = 0.76 | 463 volunteers from Boston Medical Center, Boston University, and EMC and other employed adults. [335] | Recruitment problems, which led to the study’s being under powered to detect behavior change in a randomized intervention trial. [335] |  |
| Karlsson et al., 2000 [135] | Three-Eating Factor Questionnaire (TEFQ-R21) | 7 items; rated 1-4 | composite reliability (CR) = 0.842 | The study used secondary data retrieved from a one-time survey at multi-worksites that was conducted from November survey at multi-worksites that was conducted from November 2012 to May 2013. [134] |  | Exhaustion domain of Oldenburg Burnout Inventory (OLBI-exhaustion) [133] |
| Petride et al., 2003[27]  Stamatopoulou et al., 2016 [28] | Trait Emotional Intelligence Questionnaire-Short Form (TEIQue-SF): Subscales 1) Well Being, 2) Self-Control, 3) Emotionality, and 4) Sociability | 30 items; rated 1-7 | α = 0.52 to 0.85 | nurses working in both public (52.9%) and private (47.1%) health units in Greece. [26] |  | Boredom Proneness Scale (BPS) Farmer & Sundberg, 1986 [25] |
| Dekkers et al, 2010 [180] | Trauma Screening Questionnaire: subscales 1) reexperiening, and 2) arousal | 10 items; rated yes/no |  | members of the Pediatric Association of The Netherlands [179] | low response rate, participation bias [179] | Hospital Anxiety and Depression Scale [177] |
| Mangen, Bengteon, & Landry, 1988 [48] | University of Southern California Longitudinal Study of Three-Generation Families measures of positive affect | 4 items; rated 1-4 | α = 0.85 | Data from Phase II (begun in 1993) of the Massachusetts Elder Health Project, with older persons. Phase II was on the process of caregiving, from the perspectives of both caregiver and care recipient, with the objective of defining successful caregiving. [47] | The stressors were kept separate in the analyses. The study did not have sufficient sample sizes to look at the process separately for spouses versus children.[47] |  |
| Watanabe et al., 2020 [336] | University of Tokyo Occupational Mental Health (TOMH) Well-being Scale | 24 items; rated 1-4 | α = 0.671 to 0.845 | Japanese workers [336] | Selection bias, may have been errors in measuring assessment of the standards of convergent validity, not generalizable to workers from other cultural backgrounds [336] |  |
| Utian et al., 2018 [337] | Utian Quality of Life Scale | 23 items. Rated 1-5 (Level of agreement) | α =.83 (scale as a whole) | 270 women between the ages of 45-65 years (perimenopausal and postmenopausal women) who spoke English [337] | 1) Short interval of testing (e.g., the questionnaire was administered a second time within a period of 3 to 7 days to allow assessment of test-retest reliability),  2) Construct validity of occupational and sexual factors,  3) Cross sectional study, need for longitudinal studies of randomized populations |  |
| Schaufeli et al., 2006 [112]  Seppälä et al., 2009 [36]  Scheepers et al., 2015 [208]  Schaufeli et al., 2006 [246]  Seppälä et al., 2009 [36] | Utrecht Work Engagement Scale (UWES-9); Utrecht Work Engagement Scale (shortened Italian version); Utrecht Work Engagement Scale (shortened Polish version) | Nine items; rated 0-6  Vigor Subscale (3 items), Dedication (3 items), Absorption (3 items) | α = 0.85 to 0.94  Vigor Subscale (α = 0.90), Dedication (α = 0.90), Absorption (α = 0.76) | Employees of two information and communication technology (ICT) companies [236] Multicenter questionnaire study and invited 271 surgery and gynecology residents, in 21 residency training programs, from two academic and 14 non-academic medical centers in the Netherlands. [199]  779 municipal employees in Finland. [338]; 1092 Brazilian employees. [111]; Polish police officers. [245]; 770 employees at a hospital in the south of Sweden [155]  The study population consisted of Belgian emergency physicians (N=346), who were recruited at 2 national emergency medicine conferences [33] | The sample size was small and only applicable to Finnish people; Non-representative convenience sample. [111]; Cross-sectional, may be an unrepresentative sample, more males than females. [245]; Cross-sectional, data from only one hospital [155]; Cross-sectional; under estimation of adverse consequence, in view of the fact that the study recruited at 2 Belgian emergency medicine conferences, where the emergency physicians were really engaged in sharing their workplace experiences. [33] | Mindfulness questionnaire (FMI) [235];  Satisfaction with Life Scale [237]; Stress questionnaire [238]; Work-Related Acceptance and Action Questionnaire (WAAQ) [239]; Physician Worklife Study (Dutch version) [209, 210]; Jefferson Scale of Physician Empathy (Dutch version) [202]; Bergen Burnout Inventory. [339]; Dutch Work Addiction Scale [109, 110]; Oldenburg Burnout Inventory – Polish version [243, 244]; Work Experience Measurement Scale [156]; Salutogenic Health Indicator Scale [157]; General Self Efficacy Scale [63]; Dutch version of the Checklist Individual Strength [34]; Brief Symptom Inventory [32]; Leiden Quality of Work Questionnaire for physicians (LQWQ-MD) [35] |
| Herrell et al., 2014 [128] | Walter-Reed Army Institute of Research Soldier-Specific Functional Impairment Scale | 14 items; rated 1-5 | α = 0.92 | US Army special operations soldiers [126] | findings may not be generalizable to women and conventional soldiers [126] | Emotional exhaustion scale [125]; Role Overload Scale from the Michigan Organizational Assessment questionnaire [127] |
| Tennant et al., 2007 [340] | Warwick-Edinburgh Mental Well-being Scale (WEMWBS) | 14 items; rated 1-5 | α=0.89 (student sample);  α=0.91 (population sample) | Students, working adults, and patients (one psychiatric population composed of patients with remitted schizophrenia). [341];  Sport coaching networks across Australia. [342]; Office employees at each of four Spanish universities in Galicia, the Basque Country and Catalonia. [343]; A large stratified random sample of veterinary surgeons practicing in the UK. [344]; Sit less, move more intervention was assessed at 6 Spanish University campuses. The intervention had no effect on mental well-being. [343]; 174 Australian junior cricket players attending either the male U19 National Championships or the female U18 National Championship. [345]; 13 homeless in Canada. [346]; 424 mental health employees and manager. [9] | Ex-coaches who have burnout not represented (survival bias) and lack of personal and social variables measured known to effect mental well-being. [342]; Evidence to support a range of psychometric properties for the comparator scales (i.e., Short Warwick-Edinburgh Mental Well-being Scale) is restricted to samples of other populations. [344] |  |
| Taggart et al., 2013 [347]; Bartram et al 2011 [348];  Tennant et al., 2007 [340] | Warwick-Edinburgh Mental Well-Being Scale (WEMWBS) -English version | 14 items; rated 1-5 | α = 0.88 – 0.94 | Pakistani healthcare professionals. [349]; UK veterinarians. [350]; 126 patients in Hong Kong. [351]; 148 employees at 2 emergency departments in Queensland, Australia. [352]; 744 students studying veterinary medicine, medicine, dentistry, pharmacy and law in the UK [353] | Not translated into Urdu the official language of Pakistan, convenience sample, data only collected from Punjab, cannot be generalized to the whole Pakistani population, occupational stress was measured very subjectively by presence or absence, rather than with a cross-culturally validated scale, healthcare providers were not inquired about their psychiatric health using scales for common mental illnesses. [349]; Nursing staff in a hospital in the city Kaunas, Lithuania. [354]; 72 healthy elderly people in Canada. [355] | Hospital and Anxiety Depression Scale [177]; Health and Safety Executive Management Standards Indicator Tool. [356]; Questions on suicidal ideation. [357]; Alcohol Use Disorders Identification Test. [358] |
| Ng et al., 2014 [351] | Warwick-Edinburgh Mental Well-being Scale -Chinese Short | 7 items; 5-point Likert scale from 1 to 5 | α = 0.89 | 154 participants with a clinical diagnosis of psychosis. [359] | Unblinding of participants, response bias. [359] |  |
| Schlosser, 1990 [360] | Well Being Scale (WBS-36). | 36 items on a 5-point scale. | .94 when tested on 178 healthy individuals | 39 critically injured patients in Canada. |  |  |
| Myers et al., 2020 [78]; Myers et al., 2008 [361] | Well-Being Actions Self-Efficacy Scale (WBASE) (interpersonal, community, occupational, physical, psychological, and economic) | 18 items; rated 0- 4 | Intraclass correlation coefficients ranged from 0.75 (interpersonal) to 0.84 (occupational) | Adults with obesity or overweight [78] | Self-report [78] | Expanded version of the I COPPE action scale [78, 79] |
| Williams et al., 2017 [362] | Well-Being Process Questionnaire (work characteristics, individual differences, personality, outcomes) | 25 items; 1 to 10, strongly disagree to agree strongly (except for stress, rated 1 to 5) | Average estimated reliability for the single-item measures in this study was above the 0.50 level and a range of items from demands to self-esteem and positive mood were above 0.70 | 120 university staff members aged 20-64. [363]; 3164 Irish physicians. [364] | A more representative sample of the general population, can only note casual relations, the fact that DASS-21 measured emotional states rather than diagnostic categories may be observed as a limitation | Self-Rated health [365]; The General Health Questionnaire (GHQ-12) [149]; Depression, Anxiety, Stress Scale (DASS-21) [81], one item of self-stigma |
| Bech et al., 2003 [101]  Bonsignore et al., 2014 [366]  Topp et al., 2015 [102] | WHO-5 Well-Being Index WHO-5 Well-Being Score (Danish version) | 5 items; rated 0-100 | α = 0.80 – 0.91; good construct validity and acceptable sensitivity (M=0.86) and specificity (M=0.81) | 463 volunteers from Boston Medical Center, Boston University, and EMC and other employed adults.[335]; Health professionals (majority female nurses working at a university). [367]; Employees at a major tertiary healthcare institution (the Mayo Clinic) [89]; Occupational therapy practitioners and students [368]; Healthcare Workers in Saudi Arabia During the COVID-19 Pandemic [369]; German Emergency Medical Service Workers [370]; Residents in nursing homes [289]; 10 patients receiving spasticity treatment including botulinum toxin injection and physiotherapy and/or occupational therapy. [371]; Patients with epilepsy aged ≥15 years from three outpatient clinics in Central Denmark Region. [372]; Persons with chronic suicidality as the primary presenting complaint. [373]; RCT 67 individuals with type two diabetes. [374]; Australian workplace adults. [375]; Stress-afflicted long-term sickness benefit beneficiaries in Denmark. [376]; 60 GPs and registrars working in either a full-time or part-time capacity in Emerald, Queensland. [377]; 231 physiotherapy patients with musculoskeletal disease, response rate to well-being questionnaire was 66. [378]; 93 leaders of different professions from a tertiary hospital in Germany. [379]; Arabic version of WHO-5 was used with 200 patients from six rural PHC settings in the Ismailia, Egypt governorate.  [380]; 1,164 employees nested in 30 workplaces in Canada.[381]; 237 residents from 6 communities in Shanghai. [382]; 545 Danish child protection workers. [383]; 126 patients in Hong Kong. [351]; 169 volunteers in United Kingdom [384]; 502 university employees in United Kingdom. [385]; 147 employees at an insurance company in Germany. [386] | Recruitment problems, which led to the study’s being under powered to detect behavior change in a randomized intervention trial.[335]; Single healthcare setting and small sample size [89]  Convenience sampling [368]  Healthcare workers who do not have internet access or were not familiar with online platforms were not represented [369] | 6-item Gratitude Questionnaire [387]; Neff’s Self-Compassion scale (12 items) [100]; Confidence in providing Compassionate Care Scale (10 items) [388]; Depression Anxiety Stress Scale (DASS) [81]; Self-Compassion Scale (SCS) [100]; Quality of Life in Dementia Scale (QUALID) [288] |
| Tuomi et al., 1998 [389] | Work Ability Index, WAI: Subscale categories 1) work ability compared with the lifetime best, 2) subjective work ability with regard to physical and mental demands of work, 3) current number of diagnosed diseases by the physician, 4) subjective estimated work impairment due to diseases, 5) sickness absenteeism during the past year, 6) personal prognosis for work ability 2 years from now, and 7) mental resources | 7 items; rating vary by category |  | Nationally representative sample of Finnish physicians. Nurses working in ICU, CCU and Emergency Units of hospitals at Tehran University of Medical Sciences. [212] | Not be generalizable to younger physicians or other countries [212] | Jenkin's Sleep Problem Scale Jenkins et al, 1988 [211]  Suicidal Ideation [59][60]  Work-related Quality of Life Scale [390] |
| Nilsson et al., 2010 [156] | Work Experience Measurement Scale (WEMS) | 32 statements across 6 dimensions (supportive work conditions, internal work experience, autonomy, time experience, management, process of change); six-step Likert scale ranging from totally agree to totally disagree | α = 0.71 - 0.89 | 770 employees at a hospital in the south of Sweden. [155] | Cross-sectional, data from only one hospital. [155] | Utrecht Work Engagement Scale [112]; Salutogenic Health Indicator Scale [157];  General Self Efficacy Scale [63] |
| Parker & Hyett, 2011 [391] | Work Well-Being Questionnaire | 31 items; rated 0-5 |  | 1206 patients from an internet site called black dog institute. Majority female. [391] |  |  |
| Cortina et al., 2001 [142] | Workplace Inactivity | 7 items; rated 1-5 | α = 0.89 | Nurses and doctors in the healthcare sector at two different times. [141] |  | Forgiveness Climate Cox, 2011 [140]; Satisfaction with Life Scale [139] |
| Di Fabio & Palazzeschi 2012 [5] | Workplace Relational Civility Scale (WRCS): Subscales 1) Relational readiness, 2) Relational culture, and 3) Relational decency | 26 items; rated 1-5 | α = 0.75 to 0.92 | Two hundred and sixty-one Italian workers of different public and private organizations in central Italy participated in the study. [2] | The research was conducted with a group of Italian workers who were not representative of the Italian population. [2] | Acceptance of Change Scale (ACS) [1]; Satisfaction with Life Scale [3]; The 10 Item Personality Inventory (TIPI) [4] |
| Schat & Kelloway, 2003[51] | Workplace Violence Scale-Chinese version | 5 items (one item for verbal sexual harassment, emotional abuse, physical assault, threat, and sexual assault); rated 0-3 | α = 0.942 | Doctors or nurses at two general hospitals in s in Liaoning, China [50] | cross-sectional design, self-report questionnaire [50] | The center for Epidemiologic Studies Depression Scale [43];  Psychological Capital Questionnaire [52, 53] |
| Bond et al, 2013 [239] | Work-Related Acceptance and Action Questionnaire (WAAQ) | 7 items; rated 1 (never true)-7 (always true) | Α = 0.81 to 0.84 | Employees of two information and communication technology (ICT) companies [236] |  | Mindfulness questionnaire (FMI) [235]; Satisfaction with Life Scale [237]; Stress questionnaire (Stress) [238]; Utrecht Work Engagement Scale (UWES-9) [36, 112] |
| Garzaro et al., 2020 [392] | Work-Related Quality of Life (WRQoL scale) | 32 items, Likert scale ranging from 1 to 5 | α =0.70 | Healthcare professionals (N=430) in Italy [392] | Sample was made of medium to highly educated people, Discriminant and predictive validity not tested | DASH questionnaire [393] |
| Sulaiman et al., 2015 [394] | Work-Related Quality of Life Scale-2 (WRQLS-2) in Malay language | 14 items rated 1 - 7. | α =0.60 | 272 Malaysian workers (this is psychometric paper to validate in Malay language) | Only 2 groups of workers used for the validation process. Test-retest not conducted. |  |

References

1. Di Fabio A., Kenny M.E., From decent work to decent lives: Positive Self and Relational Management (PS&RM) in the twenty-first century. Frontiers in psychology, 2016. **7**: p. 361.

2. Di Fabio A., The challenge of fostering healthy organizations: An empirical study on the role of workplace relational civility in acceptance of change and well-being. Frontiers in psychology, 2016. **7**: p. 1748.

3. Di Fabio A., Gori A., Measuring adolescent life satisfaction: Psychometric properties of the satisfaction with life scale in a sample of Italian adolescents and young adults*. Journal of Psychoeducational Assessment*, 2016. **34**(5): p. 501-506.

4. Gosling S.D., Rentfrow P.J., Swann Jr W.B., A very brief measure of the Big-Five personality domains*.* *Journal of Research in personality,* 2003. **37**(6): p. 504-528.

5. Di Fabio A., Palazzeschi L., Organizational justice: Personality traits or emotional intelligence? An empirical study in an Italian hospital context*.* *Journal of Employment Counseling*, 2012. **49**(1): p. 31-42.

6. Robinson J.P., Shaver P.R., Wrightsman L.S., Criteria for scale selection and evaluation*.* Measures of personality and social psychological attitudes, 1991. **1**: p. 1-16.

7. Yazdani F., Jibril M., Kielhofner G., A study of the relationship between variables from the Model of Human Occupation and subjective well-being among university students in Jordan*.* Occupational Therapy in Health Care, 2008. **22**(2-3): p. 125-138.

8. Bradburn N., Affect scales: Positive affect, Negative affect, Affect balance. JP Robinson, PR Shaver & LS Wrightsman (Ed. 1991), Measures of personality and social psychological attitudes, 1969. **1**: p. 83-84.

9. Stansfeld S.A., Work characteristics and personal social support as determinants of subjective well-being. PloS one, 2013. **8**(11): p. e81115.

10. Beck A.T., Steer R.A., Beck J.S., Types of self-reported anxiety in outpatients with DSM-III-R anxiety disorders1. Anxiety, stress, and coping, 1993. **6**(1): p. 43-55.

11. January A.M., Patterns of coping among caregivers of children with spinal cord injury: Associations with parent and child well-being. Families, Systems, & Health, 2019. **37**(2): p. 150.

12. Beck A.T.,Steer R.A., Brown G., Beck depression inventory–II. Psychological assessment, 1996.

13. Carver C.S., You want to measure coping but your protocol’too long: Consider the brief cope*.* *International journal of behavioral medicine*, 1997. **4**(1): p. 92-100.

14. Kovacs M., Staff M., Children's depression inventory (CDI): Technical manual update. Multi-Health Systems. Inc.: North Tonawanda, NY, USA, 2003.

15. Varni J.W., Seid M., Kurtin P.S., PedsQL™ 4.0: Reliability and validity of the Pediatric Quality of Life Inventory™ Version 4.0 Generic Core Scales in healthy and patient populations. Medical care, 2001: p. 800-812.

16. Beck A.T., Cognitive therapy of depression. 1979: Guilford press.

17. Beck A.T., An inventory for measuring depression. Archives of general psychiatry, 1961. **4**(6): p. 561-571.

18. Lis‐Turlejska M., Jewish and Non‐Jewish World War II Child and Adolescent Survivors at 60 Years After War: Effects of Parental Loss and Age at Exposure on Weil‐Being*.* *American Journal of Orthopsychiatry*, 2008. **78**(3): p. 369-377.

19. Braun S.S., Roeser R.W., Mashburn A.J., Results from a pre-post, uncontrolled pilot study of a mindfulness-based program for early elementary school teachers. Pilot and feasibility studies, 2020. **6**(1): p. 1-12.

20. Spielberger C.D., State-trait anxiety inventory for adults*.* 1983.

21. Beck, A.T., Steer R., Beck anxiety inventory (BAI). Überblick über Reliabilitäts-und Validitätsbefunde von klinischen und außerklinischen Selbst-und Fremdbeurteilungsverfahren, 1988. **7**.

22. Reynolds C.R., Kamphaus R.W., Behavior assessment system for children, (BASC-2)*.* Circle Pines, MN: American Guidance Service, 2004.

23. Belfiore L.A., Evaluation of self-concept and emotional-behavioral functioning of children with brachial plexus birth injury. *Journal of Brachial Plexus and Peripheral Nerve Injury*, 2016. **11**(01): p. e42-e47.

24. Piers E.V., Manual for the Piers-Harris Children's Self Concept Scale:(the Way I Feel about Myself). 1969: Counselor Recordings and Tests.

25. Farmer R., Sundberg N.D., Boredom proneness--the development and correlates of a new scale. *Journal of personality assessment*, 1986. **50**(1): p. 4-17.

26. Papathanasiou I.V., Emotional intelligence and professional boredom among nursing personnel in greece. *Journal of Personalized Medicine*, 2021. **11**(8): p. 750.

27. Petrides K.V., Furnham A., Trait emotional intelligence: Behavioural validation in two studies of emotion recognition and reactivity to mood induction. *European journal of personality*, 2003. **17**(1): p. 39-57.

28. Stamatopoulou M., Galanis P., Prezerakos P., Psychometric properties of the Greek translation of the trait emotional intelligence questionnaire-short form (TEIQue-SF). Personality and Individual Differences, 2016. **95**: p. 80-84.

29. Smith B.W., The brief resilience scale: assessing the ability to bounce back. *International journal of behavioral medicine*, 2008. **15**: p. 194-200.

30. Shakespeare-Finch J., Daley E., Workplace belongingness, distress, and resilience in emergency service workers. Psychological Trauma: Theory, Research, Practice, and Policy, 2017. **9**(1): p. 32.

31. Kessler R., Mroczek D., Final versions of our non-specific psychological distress scale. Ann Arbor, MI: Survey Research Center of the Institute for Social Research, University of Michigan, 1994.

32. Derogatis L.R., Brief symptom inventory: BSI. 1993: Pearson.

33. Somville F., Determinants of emergency physician wellness in Belgium. *Journal of the American College of Emergency Physicians Open*, 2020. **1**(5): p. 1013-1022.

34. Vercoulen J., Alberts M., Bleijenberg G., De checklist individuele spankracht (CIS). Gedragstherapie, 1999. **32**(131): p. 6.

35. van der Doef M., Maes S., The Leiden Quality of Work Questionnaire: its construction, factor structure, and psychometric qualities. Psychological reports, 1999. **85**(3): p. 954-962.

36. Seppälä P., The construct validity of the Utrecht Work Engagement Scale: Multisample and longitudinal evidence. *Journal of Happiness studies,* 2009. **10**: p. 459-481.

37. Glatzer W., Gulyas J., Cantril self-anchoring striving scale. Encyclopedia of quality of life and well-being research, 2014: p. 509-511.

38. Levin K.A., Currie C., Reliability and validity of an adapted version of the Cantril Ladder for use with adolescent samples*.* Social Indicators Research, 2014. **119**: p. 1047-1063.

39. CANTRIL H., The Pattern of Human Concerns. NJ: Rutgers University Press, New Brunswic1965.

40. Andrews F.M., Robinson J.P., Measures of subjective well-being. Measures of personality and social psychological attitudes: Measures of social psychological attitudes, 1991. **1**: p. 61-114.

41. Choi G., The relationship between precarious employment and subjective well-being in Korean wage workers through the Cantril ladder Scale. Annals of Occupational and Environmental Medicine, 2020. **32**.

42. Kleszczewska D., Physical activity, sedentary behaviours and duration of sleep as factors affecting the well-being of young people against the background of environmental moderators. *International journal of environmental research and public health*, 2019. **16**(6): p. 915.

43. Radloff LS. The CES-D scale: A self-report depression scale for research in the general population. *Appl Psychol Meas*. 1977. **1**(3): p. 385-401.

44. Cohen S, Kamarck T, Mermelstein R. A global measure of perceived stress*.* *J Health Soc Behav.* December, 1983: p. 385-396.

45. Smith HM, Betz NE. Development and validation of a scale of perceived social self-efficacy. *J Career Assess.* 2000. **8**(3): p. 283-301.

46. Shrout PE, Yager TJ. Reliability and validity of screening scales: Effect of reducing scale length. *J Clin Epidemiol.* 1989. **42**(1): p. 69-78.

47. Lawrence RH, Tennstedt SL, Assmann SF. Quality of the caregiver–care recipient relationship: Does it offset negative consequences of caregiving for family caregivers? *Psychol Aging*. 1998. **13**(1): p. 150.

48. Mangen DJ, Bengtson VL, Landry PH Jr. Measurement of intergenerational relations. *Res Aging*. 1988: Sage Publications, Inc.

49. Yang L, Jia C-X, Qin P. Reliability and validity of the center for epidemiologic studies depression scale (CES-D) among suicide attempters and comparison residents in rural China. *BMC Psychiatry*. 2015. **15**(1): p. 1-8.

50. Li X, Wu H. Does psychological capital mediate between workplace violence and depressive symptoms among doctors and nurses in Chinese general hospitals? *Psychol Res Behav Manag*. 2021. p. 199-206.

51. Schat ACH, Kelloway EK. Reducing the adverse consequences of workplace aggression and violence: the buffering effects of organizational support. *J Occup Health Psychol.* April, 2003. **8**(2): p. 110.

52. Li X, Kan D, Liu L, et al. The mediating role of psychological capital on the association between occupational stress and job burnout among bank employees in China*.* *Int J Environ Res Public Health.* March 10, 2015. **12**(3): p. 2984-3001.

53. Peng J, Jiang X, Zhang J, et al. The impact of psychological capital on job burnout of Chinese nurses: the mediator role of organizational commitment. *PloS One*. December 27, 2013. **8**(12): p. e84193.

54. Nyenhuis DL, Luchetta T, Yamamoto C, et al. The development, standardization, and initial validation of the Chicago multiscale depression inventory. *J Pers Assess.* April, 1998. **70**(2): p. 386-401.

55. Stern BZ, Strober L, DeLuca J, et al. Subjective well-being differs with age in multiple sclerosis: a brief report*.* *Rehabil Psychol.* August, 2018. **63**(3): p. 474.

56. Vickrey BG, Hays RD, Harooni R, et al. A health-related quality of life measure for multiple sclerosis*.* *Qual Life Res*. June, 1995. **4**(3): p. 187-206.

57. Cooper C, Katona C, Livingston G. Validity and reliability of the brief COPE in carers of people with dementia: the LASER-AD study. *J Nerv Mental Dis*. November, 2008. **196**(11): p. 838-843.

58. Ravens-Sieberer, U. and B. Monika, KINDLR Questionnaire for Measuring Health-Related Quality of Life in Children and Adolescents-Manual. 2000.

59. Kennedy-Behr A, Rodger S, Mickan S. Play or hard work: unpacking well-being at preschool*.* *Res Dev Disabil*. March, 2015. **38**: p. 30-38.

60. Evans C, Connell J, Barkham M, et al. Towards a standardised brief outcome measure: Psychometric properties and utility of the CORE–OM. *Br J Psychiatry*. January, 2002. **180**(1): p. 51-60.

61. Kellett, S., et al., The clinical and occupational effectiveness of condition management for Incapacity Benefit recipients. *British Journal of Clinical Psychology*, 2011. **50**(2): p. 164-177.

62. Mundt, J.C., et al., The Work and Social Adjustment Scale: a simple measure of impairment in functioning. *The British Journal of Psychiatry*, 2002. **180**(5): p. 461-464.

63. Schwarzer, R. and M. Jerusalem, Generalized self-efficacy scale*.* J. Weinman, S. Wright, & M. Johnston, Measures in health psychology: A user’s portfolio. Causal and control beliefs, 1995. **35**: p. 37.

64. Hackman, J.R. and E.E. Lawler, Employee reactions to job characteristics*.* *Journal of applied psychology*, 1971. **55**(3): p. 259.

65. Feldman, G., et al., Mindfulness and emotion regulation: The development and initial validation of the Cognitive and Affective Mindfulness Scale-Revised (CAMS-R). *Journal of psychopathology and Behavioral Assessment*, 2007. **29**: p. 177-190.

66. Lebares, C.C., et al., Enhanced stress resilience training in surgeons: iterative adaptation and biopsychosocial effects in 2 small randomized trials*.* Annals of surgery, 2021. **273**(3): p. 424.

67. West, C.P., et al., Concurrent validity of single-item measures of emotional exhaustion and depersonalization in burnout assessment*.* *Journal of general internal medicine*, 2012. **27**: p. 1445-1452.

68. Campbell‐Sills, L. and M.B. Stein, Psychometric analysis and refinement of the connor–davidson resilience scale (CD‐RISC): Validation of a 10‐item measure of resilience*.* *Journal of Traumatic Stress: Official Publication of The International Society for Traumatic Stress Studies*, 2007. **20**(6): p. 1019-1028.

69. Connor, K.M. and J.R. Davidson, Development of a new resilience scale: The Connor‐Davidson resilience scale (CD‐RISC). Depression and anxiety, 2003. **18**(2): p. 76-82.

70. Waddimba, A.C., et al., Resilience, well-being, and empathy among private practice physicians and advanced practice providers in Texas: A structural equation model study. Mayo Clinic Proceedings: Innovations, Quality & Outcomes, 2021. **5**(5): p. 928-945.

71. Brock, C. and J. Salinsky, Empathy: an essential skill for understanding the physician-patient relationship in clinical practice. Family medicine, 1993. **25**(4): p. 245-248.

72. Dyrbye, L.N., et al., Utility of a brief screening tool to identify physicians in distress. *Journal of general internal medicine*, 2013. **28**: p. 421-427.

73. Spreitzer, G.M., Psychological empowerment in the workplace: Dimensions, measurement, and validation. *Academy of management Journal*, 1995. **38**(5): p. 1442-1465.

74. Spreitzer, G.M., Taking stock: A review of more than twenty years of research on empowerment at work. Handbook of organizational behavior, 2008. **1**: p. 54-72.

75. Hébert, M., et al., Validation of the French Canadian version of the brief Connor–Davidson Resilience Scale (CD-RISC 10)*.* *Canadian Journal of Behavioural Science/Revue canadienne des sciences du comportement*, 2018. **50**(1): p. 9.

76. Arsalani, N., et al., Adaptation of questionnaire measuring working conditions and health problems among Iranian nursing personnel. Asian nursing research, 2011. **5**(3): p. 177-182.

77. Aminian, O., A. Moradi, and S. Eftekhari, Assessment of psychosocial factors in office and operational groups of employees of a Regional Electricity Distribution Company in Iran–A case study*.* Heliyon, 2018. **4**(8).

78. Myers, N.D., et al., Effectiveness of the fun for wellness web-based behavioral intervention to promote physical activity in adults with obesity (or overweight): Randomized controlled trial. JMIR formative research, 2020. **4**(2): p. e15919.

79. Myers, N.D., et al., Efficacy of the fun for wellness online intervention to promote multidimensional well-being: A randomized controlled trial. Prevention Science, 2017. **18**: p. 984-994.

80. Prilleltensky, I., et al., Assessing multidimensional well‐being: Development and validation of the I COPPE scale*.* *Journal of Community Psychology*, 2015. **43**(2): p. 199-226.

81. Lovibond, P.F. and S.H. Lovibond, The structure of negative emotional states: Comparison of the Depression Anxiety Stress Scales (DASS) with the Beck Depression and Anxiety Inventories. Behaviour research and therapy, 1995. **33**(3): p. 335-343.

82. Henry, J.D. and J.R. Crawford, The short‐form version of the Depression Anxiety Stress Scales (DASS‐21): Construct validity and normative data in a large non‐clinical sample*.* *British journal of clinical psychology*, 2005. **44**(2): p. 227-239.

83. Dreyer, Z., C. Henn, and C. Hill, Validation of the Depression Anxiety Stress Scale-21 (DASS-21) in a non-clinical sample of South African working adults. *Journal of psychology in Africa*, 2019. **29**(4): p. 346-353.

84. Tran, T.D., T. Tran, and J. Fisher, Validation of the depression anxiety stress scales (DASS) 21 as a screening instrument for depression and anxiety in a rural community-based cohort of northern Vietnamese women*.* BMC psychiatry, 2013. **13**(1): p. 1-7.

85. Chan, R.C., et al., Extending the utility of the Depression Anxiety Stress scale by examining its psychometric properties in Chinese settings. Psychiatry research, 2012. **200**(2-3): p. 879-883.

86. Patrick, J., M. Dyck, and P. Bramston, Depression Anxiety Stress Scale: is it valid for children and adolescents? *Journal of clinical psychology*, 2010. **66**(9): p. 996-1007.

87. Almhdawi, K.A., et al., Study-related mental health symptoms and their correlates among allied health professions students. Work, 2018. **61**(3): p. 391-401.

88. Vendrig, A. and F. Schaafsma, Reliability and validity of the work and well-being inventory (WBI) for employees. *Journal of Occupational Rehabilitation*, 2018. **28**: p. 377-390.

89. Mistretta, E.G., et al., *Resilience training for work-related stress among health care workers: results of a randomized clinical trial comparing in-person and smartphone-delivered interventions.* Journal of occupational and environmental medicine, 2018. **60**(6): p. 559-568.

90. Mahat, N., et al., *Paediatric surgical response to an ‘adult’COVID-19 pandemic.* Med J Malaysia, 2021. **76**(3): p. 285.

91. Harris, L.M., S.R. Cumming, and A.J. Campbell, *Stress and psychological well-being among allied health professionals.* Journal of allied health, 2006. **35**(4): p. 198-207.

92. Wynter, K., et al., *Hospital clinicians’ psychosocial well-being during the COVID-19 pandemic: longitudinal study.* Occupational Medicine, 2022. **72**(3): p. 215-224.

93. Vujcic, M., et al., *Nature based solution for improving mental health and well-being in urban areas.* Environmental research, 2017. **158**: p. 385-392.

94. Jackson, M.L., et al., *Adding access to a video magnifier to standard vision rehabilitation: initial results on reading performance and well-being from a prospective, randomized study.* Digital Journal of Ophthalmology: DJO, 2017. **23**(1): p. 1.

95. Migliorini, C., et al., *A randomised control trial of an Internet-based cognitive behaviour treatment for mood disorder in adults with chronic spinal cord injury.* Spinal Cord, 2016. **54**(9): p. 695-701.

96. Chen, I.-H., et al., *Evaluating the immediate and delayed effects of psychological need thwarting of online teaching on Chinese primary and middle school teachers’ psychological well-being.* Frontiers in Psychology, 2022. **13**: p. 943449.

97. Almhdawi, K.A., et al., *University professors’ mental and physical well-being during the COVID-19 pandemic and distance teaching.* Work, 2021. **69**(4): p. 1153-1161.

98. Zimet, G.D., et al., *The multidimensional scale of perceived social support.* Journal of personality assessment, 1988. **52**(1): p. 30-41.

99. Zimet, G.D., et al., *Psychometric characteristics of the multidimensional scale of perceived social support.* Journal of personality assessment, 1990. **55**(3-4): p. 610-617.

100. Neff, K.D., *The development and validation of a scale to measure self-compassion.* Self and identity, 2003. **2**(3): p. 223-250.

101. Bech, P., et al., *Measuring well‐being rather than the absence of distress symptoms: a comparison of the SF‐36 Mental Health subscale and the WHO‐Five well‐being scale.* International journal of methods in psychiatric research, 2003. **12**(2): p. 85-91.

102. Topp, C.W., et al., *The WHO-5 Well-Being Index: a systematic review of the literature.* Psychotherapy and psychosomatics, 2015. **84**(3): p. 167-176.

103. Diener, E., et al., *The life satisfaction scale.* Journal of personality Assessment, 1985. **49**(1): p. 71-75.

104. Lamoureux, E.L., et al., *The Impact of Vision Impairment Questionnaire: an evaluation of its measurement properties using Rasch analysis.* Investigative ophthalmology & visual science, 2006. **47**(11): p. 4732-4741.

105. Cummins, R., *International Well Being Group: Personal Wellbeing Index.* Melbourne: Australian Centre on Quality of Life, Deakin University, 2004.

106. Citko, A., et al., *Sedentary lifestyle and nonspecific low back pain in medical personnel in North-East Poland.* BioMed research international, 2018. **2018**.

107. Powell, K.E., A.E. Paluch, and S.N. Blair, *Physical activity for health: What kind? How much? How intense? On top of what?* Annual review of public health, 2011. **32**: p. 349-365.

108. Helou, K., et al., *Validity and reliability of an adapted arabic version of the long international physical activity questionnaire.* BMC public health, 2018. **18**(1): p. 1-8.

109. Schaufeli, W.B., A. Shimazu, and T.W. Taris, *Being driven to work excessively hard: The evaluation of a two-factor measure of workaholism in the Netherlands and Japan.* Cross-cultural research, 2009. **43**(4): p. 320-348.

110. Schaufeli, W.B., T.W. Taris, and A.B. Bakker, *It takes two to tango: Workaholism is working excessively and working compulsively.* The long work hours culture: Causes, consequences and choices, 2008: p. 203-226.

111. Devotto, R.P.d., C.P.P.d. Freitas, and S.M. Wechsler, *Perceived Opportunities to Craft Scale: adaptation and evidence of the construct validity of the Brazilian version.* Psicologia: Reflexão e Crítica, 2020. **33**.

112. Schaufeli, W.B., A. Baker, and M. Salanova, *Educational and Psychological Measurement.* The measurement of work engagement with a short questionnaire. A Cross-National Study, 2006. **66**(4).

113. Cox, J.L., J.M. Holden, and R. Sagovsky, *Detection of postnatal depression: development of the 10-item Edinburgh Postnatal Depression Scale.* The British journal of psychiatry, 1987. **150**(6): p. 782-786.

114. Hoffenkamp, H.N., et al., *Effectiveness of hospital-based video interaction guidance on parental interactive behavior, bonding, and stress after preterm birth: A randomized controlled trial.* Journal of Consulting and Clinical Psychology, 2015. **83**(2): p. 416.

115. Cooklin, A.R., H.J. Rowe, and J.R. Fisher, *Employee entitlements during pregnancy and maternal psychological well‐being.* Australian and New Zealand Journal of Obstetrics and Gynaecology, 2007. **47**(6): p. 483-490.

116. McNair, D.M., M. Lorr, and L.F. Droppleman, *Manual profile of mood states.* 1971.

117. Li, J., et al., *Effort–reward imbalance at work and job dissatisfaction in Chinese healthcare workers: a validation study.* International archives of occupational and environmental health, 2005. **78**: p. 198-204.

118. Wang, Z., et al., *Associations between occupational stress, burnout and well-being among manufacturing workers: mediating roles of psychological capital and self-esteem.* BMC psychiatry, 2017. **17**: p. 1-10.

119. Diener, E., et al., *New well-being measures: Short scales to assess flourishing and positive and negative feelings.* Social indicators research, 2010. **97**: p. 143-156.

120. Maslach, C. and S.E. Jackson, *The measurement of experienced burnout.* Journal of organizational behavior, 1981. **2**(2): p. 99-113.

121. Schaufeli, W.B., *Maslach burnout inventory-general survey (MBI-GS).* Maslach burnout inventory manual, 1996.

122. LI, C., K. Shi, and Z. Luo, *Work-family conflict and job burnout of doctors and nurses.* Chinese Mental Health Journal, 2002.

123. Luthans, F., et al., *Positive psychological capital: Measurement and relationship with performance and satisfaction.* Personnel psychology, 2007. **60**(3): p. 541-572.

124. Cheng, S.-T. and P.N. Hamid, *An error in the use of translated scales: The Rosenberg Self-Esteem Scale for Chinese.* Perceptual and Motor Skills, 1995. **81**(2): p. 431-434.

125. Maslach, C., et al., *Maslach Burnout Inventory (Vol. 21): Consulting psychologists*. 1986, CA: press Palo Alto.

126. Mantua, J., et al., *Sleep and occupational well-being in active duty special operations soldiers: a replication and expansion.* Sleep Health, 2021. **7**(4): p. 500-503.

127. Cammann, C., et al., *The Michigan organizational assessment questionnaire.* Unpublished manuscript, University of Michigan, Ann Arbor, 1979. **71**: p. 138.

128. Herrell, R.K., et al., *Assessing functional impairment in a working military population: the Walter Reed functional impairment scale.* Psychological Services, 2014. **11**(3): p. 254.

129. Cohen, S., *Perceived Stress Scale. USA: Mind Garden*. 1994, Inc.

130. Rabin, R. and F.d. Charro, *EQ-SD: a measure of health status from the EuroQol Group.* Annals of medicine, 2001. **33**(5): p. 337-343.

131. Group, T.E., *EuroQol-a new facility for the measurement of health-related quality of life.* Health policy, 1990. **16**(3): p. 199-208.

132. Sauni, R., et al., *Quality of life (EQ-5D) and hand-arm vibration syndrome.* International archives of occupational and environmental health, 2010. **83**: p. 209-216.

133. Schaufeli, W.B. and A.B. Bakker, *Job demands, job resources, and their relationship with burnout and engagement: A multi‐sample study.* Journal of Organizational Behavior: The International Journal of Industrial, Occupational and Organizational Psychology and Behavior, 2004. **25**(3): p. 293-315.

134. Abidin, N.D.I.Z., R. Ismail, and N.H. Ismail, *Psychosocial stressors at work and well-being of male employees.* Med J Malaysia, 2019. **74**(2): p. 161.

135. Karlsson, J., et al., *Psychometric properties and factor structure of the Three-Factor Eating Questionnaire (TFEQ) in obese men and women. Results from the Swedish Obese Subjects (SOS) study.* International journal of obesity, 2000. **24**(12): p. 1715-1725.

136. Sumi, K., *Reliability and validity of Japanese versions of the Flourishing Scale and the Scale of Positive and Negative Experience.* Social Indicators Research, 2014. **118**: p. 601-615.

137. Izumi, K., et al., *Unobtrusive sensing technology for quantifying stress and well-being using pulse, speech, body motion, and electrodermal data in a workplace setting: Study concept and design.* Frontiers in Psychiatry, 2021. **12**: p. 611243.

138. Sato, T., *Development of the Japanese version of positive and negative affect achedule (PANAS) scales.* Jpn J Pers, 2001. **9**: p. 138.

139. Diener, E., et al., *The satisfaction with life scale.* Journal of personality assessment, 1985. **49**(1): p. 71-75.

140. Cox, S.S. *An investigation of forgiveness climate and workplace outcomes*. in *Academy of Management Proceedings*. 2011. Academy of Management Briarcliff Manor, NY 10510.

141. Khan, M.S., N.S. Elahi, and G. Abid, *Workplace incivility and job satisfaction: Mediation of subjective well-being and moderation of forgiveness climate in health care sector.* European Journal of Investigation in Health, Psychology and Education, 2021. **11**(4): p. 1107-1119.

142. Cortina, L.M., et al., *Incivility in the workplace: incidence and impact.* Journal of occupational health psychology, 2001. **6**(1): p. 64.

143. Cella, D.F., et al., *The Functional Assessment of Cancer Therapy scale: development and validation of the general measure.* J Clin Oncol, 1993. **11**(3): p. 570-579.

144. Van Heest, K.N., A.R. Mogush, and V.G. Mathiowetz, *Effects of a one-to-one fatigue management course for people with chronic conditions and fatigue.* The American Journal of Occupational Therapy, 2017. **71**(4): p. 7104100020p1-7104100020p9.

145. Peterman, A.H., et al., *Measuring spiritual well-being in people with cancer: the functional assessment of chronic illness therapy—Spiritual Well-being Scale (FACIT-Sp).* Annals of behavioral medicine, 2002. **24**(1): p. 49-58.

146. Lazenby, M., et al., *Psychometric properties of the Functional Assessment of Chronic Illness Therapy—Spiritual Well‐being (FACIT‐Sp) in an Arabic‐speaking, predominantly Muslim population.* Psycho‐oncology, 2013. **22**(1): p. 220-227.

147. Chaar, E.A., et al., *Evaluating the impact of spirituality on the quality of life, anxiety, and depression among patients with cancer: an observational transversal study.* Supportive Care in Cancer, 2018. **26**: p. 2581-2590.

148. Chirico, F., et al., *Spirituality and prayer on teacher stress and burnout in an Italian cohort: A pilot, before-after controlled study.* Frontiers in Psychology, 2020. **10**: p. 2933.

149. Goldberg, D., et al., *Detecting anxiety and depression in general medical settings.* British Medical Journal, 1988. **297**(6653): p. 897-899.

150. Kawada, T., *Glucose intolerance and General Health Questionnaire 12-item version scores of male two-shift workers stratified by precariousness of work.* Diabetes & Metabolic Syndrome: Clinical Research & Reviews, 2016. **10**(2): p. 75-77.

151. Hedlund, Å., et al., *Predictors of return to work and psychological well‐being among women during/after long‐term sick leave due to common mental disorders‐a prospective cohort study based on the theory of planned behaviour.* Health & Social Care in the Community, 2022. **30**(6): p. e5245-e5258.

152. Bridger, R.S. and K. Brasher, *Cognitive task demands, self-control demands and the mental well-being of office workers.* Ergonomics, 2011. **54**(9): p. 830-839.

153. Sluiter, J., et al., *Need for recovery from work related fatigue and its role in the development and prediction of subjective health complaints.* Occupational and environmental medicine, 2003. **60**(suppl 1): p. i62-i70.

154. Eltayeb, S., et al., *Prevalence of complaints of arm, neck and shoulder among computer office workers and psychometric evaluation of a risk factor questionnaire.* BMC musculoskeletal disorders, 2007. **8**: p. 1-11.

155. Nilsson, P., H.I. Andersson, and G. Ejlertsson, *The Work Experience Measurement Scale (WEMS): A useful tool in workplace health promotion.* Work, 2013. **45**(3): p. 379-387.

156. Nilsson, P., *Development and quality analysis of the Work Experience Measurement Scale (WEMS).* Work, 2010. **35**(2): p. 153-161.

157. Bringsén, Å., H.I. Andersson, and G. Ejlertsson, *Development and quality analysis of the Salutogenic Health Indicator Scale (SHIS).* Scandinavian Journal of Public Health, 2009. **37**(1): p. 13-19.

158. Yesavage, J.A., et al., *Development and validation of a geriatric depression screening scale: a preliminary report.* Journal of psychiatric research, 1982. **17**(1): p. 37-49.

159. Yuen, H.K., et al., *Impact of participating in volunteer activities for residents living in long-term-care facilities.* The American Journal of Occupational Therapy, 2008. **62**(1): p. 71-76.

160. Kobylańska, M., et al., *The role of biopsychosocial factors in the rehabilitation process of individuals with a stroke.* Work, 2018. **61**(4): p. 523-535.

161. Neugarten, B., *RJ Havighurst, and SS Tobin1961" The measurement of life satisfaction." J. of Gerontology 16: 134-143.* 1961.

162. Tehrani, N., *Psychological well-being and workability in child abuse investigators.* Occupational medicine, 2018. **68**(3): p. 165-170.

163. Magnavita, N., G. Tripepi, and C. Chiorri, *Telecommuting, off-time work, and intrusive leadership in workers’ well-being.* International Journal of Environmental Research and Public Health, 2021. **18**(7): p. 3330.

164. Tehrani, N., S.J. Cox, and T. Cox, *Assessing the impact of stressful incidents in organizations: The development of an extended impact of events scale.* Counselling Psychology Quarterly, 2002. **15**(2): p. 191-200.

165. Stamm, B., *The concise manual for the professional quality of life scale.* 2010.

166. Ghislieri, C., et al., *New technologies smart, or harm work-family boundaries management? Gender differences in conflict and enrichment using the JD-R theory.* Frontiers in psychology, 2017. **8**: p. 1070.

167. Schmidt, A.A., *Development and validation of the toxic leadership scale*. 2008: University of Maryland, College Park.

168. Andreassen, C.S., et al., *Development of a work addiction scale.* Scandinavian journal of psychology, 2012. **53**(3): p. 265-272.

169. Siegrist, J., *Adverse health effects of high-effort/low-reward conditions.* Journal of occupational health psychology, 1996. **1**(1): p. 27.

170. Tibblin, G., et al., *" The Göteborg quality of life instrument"--an assessment of well-being and symptoms among men born 1913 and 1923. Methods and validity.* Scandinavian journal of primary health care. Supplement, 1990. **1**: p. 33-38.

171. Håkansson, C., et al., *Well-being and occupational roles among middle-aged women.* Work, 2005. **24**(4): p. 341-351.

172. Cederlund, R., S. Iwarsson, and G. Lundborg, *Quality of life in Swedish workers exposed to hand–arm vibration.* Occupational therapy international, 2007. **14**(3): p. 156-169.

173. Schalock, R.L., G.S. Bonham, and M.A. Verdugo, *The conceptualization and measurement of quality of life: Implications for program planning and evaluation in the field of intellectual disabilities.* Evaluation and program planning, 2008. **31**(2): p. 181-190.

174. Pett, M.A., et al., *Psychometric properties of a brief self‐reported health‐related quality of life measure (HRQoL‐IDD) for persons with intellectual and developmental disabilities.* Journal of applied research in intellectual disabilities, 2021. **34**(3): p. 877-890.

175. Hesbacher, P.T., et al., *Psychiatric illness in family practice.* The Journal of clinical psychiatry, 1980. **41**(1): p. 6-10.

176. Winokur, A., et al., *Symptoms of emotional distress in a family planning service: stability over a four-week period.* The British Journal of Psychiatry, 1984. **144**(4): p. 395-399.

177. Zigmond, A.S. and R.P. Snaith, *The hospital anxiety and depression scale.* Acta psychiatrica scandinavica, 1983. **67**(6): p. 361-370.

178. Bocéréan, C. and E. Dupret, *A validation study of the Hospital Anxiety and Depression Scale (HADS) in a large sample of French employees.* BMC psychiatry, 2014. **14**(1): p. 1-11.

179. van Steijn, M.E., et al., *Occupational well-being in pediatricians—a survey about work-related posttraumatic stress, depression, and anxiety.* European journal of pediatrics, 2019. **178**: p. 681-693.

180. Dekkers, A., M. Olff, and G. Näring, *Identifying persons at risk for PTSD after trauma with TSQ in the Netherlands.* Community mental health journal, 2010. **46**: p. 20-25.

181. Pennebaker, J.W., *Pennebaker Inventory of Limbic Languidness.* 1982.

182. Kristensen, T.S., et al., *The Copenhagen Burnout Inventory: A new tool for the assessment of burnout.* Work & stress, 2005. **19**(3): p. 192-207.

183. Morin, C.M., et al., *The Insomnia Severity Index: psychometric indicators to detect insomnia cases and evaluate treatment response.* Sleep, 2011. **34**(5): p. 601-608.

184. Sagherian, K., et al., *Insomnia, fatigue and psychosocial well‐being during COVID‐19 pandemic: A cross‐sectional survey of hospital nursing staff in the United States.* Journal of clinical nursing, 2023. **32**(15-16): p. 5382-5395.

185. Sagherian, K., H. Cho, and L.M. Steege, *The insomnia, fatigue, and psychological well‐being of hospital nurses 18 months after the COVID‐19 pandemic began: A cross‐sectional study.* Journal of clinical nursing, 2022.

186. Winwood, P., et al., *Development and validation of a scale to measure work-related fatigue and recovery: the Occupational Fatigue Exhaustion/Recovery Scale (OFER).* Journal of occupational and environmental medicine, 2005: p. 594-606.

187. Winwood, P.C., K. Lushington, and A.H. Winefield, *Further development and validation of the Occupational Fatigue Exhaustion Recovery (OFER) scale.* Journal of Occupational and Environmental Medicine, 2006: p. 381-389.

188. Connor, K.M. and J.R. Davidson, *SPRINT: A brief global assessment of post-traumatic stress disorder.* International clinical psychopharmacology, 2001. **16**(5): p. 279-284.

189. Davidson, J. and J. Colket, *The eight-item treatment-outcome post-traumatic stress disorder scale: a brief measure to assess treatment outcome in post-traumatic stress disorder.* International Clinical Psychopharmacology, 1997. **12**(1): p. 41-46.

190. Kroenke, K., et al., *An ultra-brief screening scale for anxiety and depression: the PHQ–4.* Psychosomatics, 2009. **50**(6): p. 613-621.

191. Löwe, B., et al., *A 4-item measure of depression and anxiety: validation and standardization of the Patient Health Questionnaire-4 (PHQ-4) in the general population.* Journal of affective disorders, 2010. **122**(1-2): p. 86-95.

192. Ware Jr, J.E., M. Kosinski, and S.D. Keller, *A 12-Item Short-Form Health Survey: construction of scales and preliminary tests of reliability and validity.* Medical care, 1996: p. 220-233.

193. Al Sayah, F., et al., *Health related quality of life measures in Arabic speaking populations: a systematic review on cross-cultural adaptation and measurement properties.* Quality of Life Research, 2013. **22**: p. 213-229.

194. Vernon, H. and S. Mior, *The Neck Disability Index: a study of reliability and validity.* Journal of manipulative and physiological therapeutics, 1991. **14**(7): p. 409-415.

195. Shaheen, A.A.M., M.T.A. Omar, and H. Vernon, *Cross-cultural adaptation, reliability, and validity of the Arabic version of neck disability index in patients with neck pain.* Spine, 2013. **38**(10): p. E609-E615.

196. Mohr, G., et al., *The assessment of psychological strain in work contexts.* European Journal of Psychological Assessment, 2006. **22**(3): p. 198-206.

197. Weber, J., et al., *Country‐specific differences of age stereotypes towards older hospital staff and their association with self‐efficacy, work ability and mental well‐being.* Journal of Advanced Nursing, 2020. **76**(7): p. 1614-1626.

198. Hojat, M., et al., *Psychometric properties of an attitude scale measuring physician-nurse collaboration.* Evaluation & the Health Professions, 1999. **22**(2): p. 208-220.

199. Lases, L.S., et al., *Learning climate positively influences residents’ work-related well-being.* Advances in Health Sciences Education, 2019. **24**: p. 317-330.

200. Muliira, J.K., et al., *Nurses’ orientation toward lifelong learning: a case study of Uganda’s National Hospital.* The journal of continuing education in nursing, 2012. **43**(2): p. 90-96.

201. Alcorta-Garza, A., et al., *Cross-validation of the Spanish HP-version of the jefferson scale of empathy confirmed with some cross-cultural differences.* Frontiers in psychology, 2016. **7**: p. 1002.

202. Hojat, M., et al., *The Jefferson Scale of Physician Empathy: further psychometric data and differences by gender and specialty at item level.* Academic medicine, 2002. **77**(10): p. S58-S60.

203. Glaser, K.M., et al., *Relationships between scores on the Jefferson Scale of physician empathy, patient perceptions of physician empathy, and humanistic approaches to patient care: a validity study.* 2007.

204. Soler-Gonzalez, J., et al., *Human connections and their roles in the occupational well-being of healthcare professionals: a study on loneliness and empathy.* Frontiers in Psychology, 2017. **8**: p. 1475.

205. DiTommaso, E., C. Brannen, and L.A. Best, *Measurement and validity characteristics of the short version of the social and emotional loneliness scale for adults.* Educational and Psychological Measurement, 2004. **64**(1): p. 99-119.

206. Domínguez, V., M. San-Martín, and L. Vivanco, *Relaciones familiares, soledad y empatía en el cuidado del paciente en estudiantes de enfermería.* Atención Primaria, 2017. **49**(1): p. 56.

207. Caro, M.M., et al., *Empathy, loneliness, burnout, and life satisfaction in Chilean nurses of palliative care and homecare services.* Enfermería Clínica (English Edition), 2017. **27**(6): p. 379-386.

208. Scheepers, R.A., et al., *In the eyes of residents good supervisors need to be more than engaged physicians: the relevance of teacher work engagement in residency training.* Advances in Health Sciences Education, 2015. **20**: p. 441-455.

209. Williams, E.S., et al., *Refining the measurement of physician job satisfaction: results from the Physician Worklife Survey.* Medical care, 1999: p. 1140-1154.

210. Konrad, T.R., et al., *Measuring physician job satisfaction in a changing workplace and a challenging environment.* Medical care, 1999: p. 1174-1182.

211. CD, J., *Stanton B. Niemcryk S. Rose RA scale for the estimation of sleep problems in clinical research.* J Clin Epidemiol, 1988. **41**: p. 313-321.

212. Mullola, S., et al., *Medical specialty choice and well-being at work: Physician's personality as a moderator.* Archives of environmental & occupational health, 2019. **74**(3): p. 115-129.

213. Ravens-Sieberer, U., et al., *The KIDSCREEN-52 quality of life measure for children and adolescents: psychometric results from a cross-cultural survey in 13 European countries.* Value in health, 2008. **11**(4): p. 645-658.

214. Madsen, M., et al., *The “11 for Health in Denmark” intervention in 10‐to 12‐year‐old Danish girls and boys and its effects on well‐being—a large‐scale cluster RCT.* Scandinavian journal of medicine & science in sports, 2020. **30**(9): p. 1787-1795.

215. Fugl-Meyer, A.R., R. Melin, and K.S. Fugl-Meyer, *Life satisfaction in 18-to 64-year-old Swedes: in relation to gender, age, partner and immigrant status.* Journal of rehabilitation medicine, 2002. **34**(5): p. 239-246.

216. Maujean, A. and P. Davis, *The relationship between self-efficacy and well-being in stroke survivors.* International Journal of Physical Medicine & Rehabilitation, 2013. **1**(7): p. 159.

217. Rashid, M., et al., *Factors related to work ability and well-being among women on sick leave due to long-term pain in the neck/shoulders and/or back: a cross-sectional study.* BMC Public Health, 2018. **18**: p. 1-8.

218. Bobbio, A., L. Canova, and A.M. Manganelli, *Organizational work-home culture and its relations with the work–family interface and employees’ subjective well-being.* Applied Research in Quality of Life, 2022. **17**(5): p. 2933-2966.

219. Hurtado, D.A., P. Hessel, and M. Avendano, *The hidden costs of informal work: Lack of social protection and subjective well-being in Colombia.* International journal of public health, 2017. **62**: p. 187-196.

220. Park, H., et al., *Character profiles and life satisfaction.* Comprehensive psychiatry, 2015. **58**: p. 172-177.

221. Maslach, C., S.E. Jackson, and M.P. Leiter, *Maslach burnout inventory*. 1997: Scarecrow Education.

222. Pisanti, R., et al., *Psychometric properties of the Maslach Burnout Inventory for Human Services among Italian nurses: a test of alternative models.* Journal of Advanced Nursing, 2013. **69**(3): p. 697-707.

223. Carstensen, B. and U. Klusmann, *Assertiveness and adaptation: Prospective teachers’ social competence development and its significance for occupational well‐being.* British Journal of Educational Psychology, 2021. **91**(1): p. 500-526.

224. McQuivey, K.S., et al., *Surgical ergonomics and musculoskeletal pain in arthroplasty surgeons.* The Journal of Arthroplasty, 2021. **36**(11): p. 3781-3787. e7.

225. Hayes, B., et al., *Doctors don’t Do-little: a national cross-sectional study of workplace well-being of hospital doctors in Ireland.* BMJ open, 2019. **9**(3): p. e025433.

226. Pisanti, R., et al., *Psychosocial job characteristics and psychological distress/well‐being: the mediating role of personal goal facilitation.* Journal of occupational health, 2016. **58**(1): p. 36-46.

227. Pierce, R.G., et al., *Results from the National Taskforce for Humanity in Healthcare's Integrated, Organizational Pilot Program to Improve Well-Being.* The Joint Commission Journal on Quality and Patient Safety, 2021. **47**(9): p. 581-590.

228. McManus, I., B. Winder, and D. Gordon, *The causal links between stress and burnout in a longitudinal study of UK doctors.* The Lancet, 2002. **359**(9323): p. 2089-2090.

229. Dyrbye, L.N., et al., *Development and preliminary psychometric properties of a well-being index for medical students.* BMC medical education, 2010. **10**: p. 1-9.

230. Forycka, J., et al., *Polish medical students facing the pandemic—Assessment of resilience, well-being and burnout in the COVID-19 era.* PLoS One, 2022. **17**(1): p. e0261652.

231. Keyes, C.L., *Mental illness and/or mental health? Investigating axioms of the complete state model of health.* Journal of consulting and clinical psychology, 2005. **73**(3): p. 539.

232. Hone, L.C., et al., *Measuring flourishing: The impact of operational definitions on the prevalence of high levels of wellbeing.* 2014.

233. Fan, J.K., C. Mustard, and P.M. Smith, *Psychosocial work conditions and mental health: examining differences across mental illness and well-being outcomes.* Annals of Work Exposures and Health, 2019. **63**(5): p. 546-559.

234. Ngooi, B.X., et al., *Exploring the use of activity-based group therapy in increasing self-efficacy and subjective well-being in acute mental health.* Hong Kong Journal of Occupational Therapy, 2022. **35**(1): p. 52-61.

235. Walach, H., et al., *Measuring mindfulness—the Freiburg mindfulness inventory (FMI).* Personality and individual differences, 2006. **40**(8): p. 1543-1555.

236. Muuraiskangas, S., et al., *Process and effects evaluation of a digital mental health intervention targeted at improving occupational well-being: lessons from an intervention study with failed adoption.* JMIR mental health, 2016. **3**(2): p. e4465.

237. Pavot, W. and E. Diener, *The satisfaction with life scale and the emerging construct of life satisfaction.* The journal of positive psychology, 2008. **3**(2): p. 137-152.

238. Elo, A. and A. Leppanen. *Can a single-item measure of stress be valid?* in *Work and Stress*. 2000. TAYLOR & FRANCIS LTD 4 PARK SQUARE, MILTON PARK, ABINGDON OX14 4RN, OXON ….

239. Bond, F.W., J. Lloyd, and N. Guenole, *The work‐related acceptance and action questionnaire: Initial psychometric findings and their implications for measuring psychological flexibility in specific contexts.* Journal of occupational and organizational psychology, 2013. **86**(3): p. 331-347.

240. Baele, C.A. and J.R. Fontaine, *The Moral Distress‐Appraisal Scale: Scale development and validation study.* Journal of Advanced Nursing, 2021. **77**(10): p. 4120-4130.

241. Van Veldhoven, M. and S. Broersen, *Measurement quality and validity of the “need for recovery scale”.* Occupational and environmental medicine, 2003. **60**(suppl 1): p. i3-i9.

242. Cottey, L., et al., *Need for recovery and physician well-being in emergency departments: national survey findings.* European Journal of Emergency Medicine, 2021. **28**(5): p. 386-393.

243. Demerouti, E., et al., *The convergent validity of two burnout instruments: A multitrait-multimethod analysis.* European Journal of Psychological Assessment, 2003. **19**(1): p. 12.

244. Demerouti, E., K. Mostert, and A.B. Bakker, *Burnout and work engagement: a thorough investigation of the independency of both constructs.* Journal of occupational health psychology, 2010. **15**(3): p. 209.

245. Basinska, B.A. and A.M. Dåderman, *Work values of police officers and their relationship with job burnout and work engagement.* Frontiers in psychology, 2019. **10**: p. 442.

246. Schaufeli, W.B., A.B. Bakker, and M. Salanova, *The measurement of work engagement with a short questionnaire: A cross-national study.* Educational and psychological measurement, 2006. **66**(4): p. 701-716.

247. Peto, V., et al., *The development and validation of a short measure of functioning and well being for individuals with Parkinson's disease.* Quality of life research, 1995. **4**: p. 241-248.

248. Harris, R.C., et al., *Effects of parkinsonism on health status in welding exposed workers.* Parkinsonism & related disorders, 2011. **17**(9): p. 672-676.

249. Kansiewicz, K.M., et al., *Well-being and help-seeking among assemblies of god ministers in the USA.* Journal of religion and health, 2022. **61**(2): p. 1242-1260.

250. Hybels, C.F., et al., *Age differences in trajectories of depressive, anxiety, and burnout symptoms in a population with a high likelihood of persistent occupational distress.* International Psychogeriatrics, 2022. **34**(1): p. 21-32.

251. Lane, A., et al., *Worried, weary and worn out: mixed-method study of stress and well-being in final-year medical students.* BMJ open, 2020. **10**(12): p. e040245.

252. Tamim, H., et al., *Tai Chi workplace program for improving musculoskeletal fitness among female computer users.* Work, 2009. **34**(3): p. 331-338.

253. Luthar, S.S. and L. Ciciolla, *Who mothers mommy? Factors that contribute to mothers’ well-being.* Developmental Psychology, 2015. **51**(12): p. 1812.

254. Butler, J. and M.L. Kern, *The PERMA-Profiler: A brief multidimensional measure of flourishing.* International Journal of Wellbeing, 2016. **6**(3).

255. Yang, C.-C., K. Watanabe, and N. Kawakami, *The associations between job strain, workplace PERMA profiler, and work engagement.* Journal of Occupational and Environmental Medicine, 2022. **64**(5): p. 409-415.

256. Shimomitsu, T., *The final development of the Brief Job Stress Questionnaire mainly used for assessment of the individuals.* Ministry of Labour sponsored grant for the prevention of work-related illness: The 1999 report, 2000: p. 126-164.

257. Shimazu, A., et al., *Work engagement in Japan: validation of the Japanese version of the Utrecht Work Engagement Scale.* Applied Psychology, 2008. **57**(3): p. 510-523.

258. Trockel, M., et al., *A brief instrument to assess both burnout and professional fulfillment in physicians: reliability and validity, including correlation with self-reported medical errors, in a sample of resident and practicing physicians.* Academic Psychiatry, 2018. **42**: p. 11-24.

259. Hamidi, M.S., et al., *Estimating institutional physician turnover attributable to self-reported burnout and associated financial burden: a case study.* BMC health services research, 2018. **18**: p. 1-8.

260. Brady, K.J., et al., *Establishing crosswalks between common measures of burnout in US physicians.* Journal of General Internal Medicine, 2021: p. 1-8.

261. Rowe, S.G., et al., *Mistreatment experiences, protective workplace systems, and occupational distress in physicians.* JAMA Network Open, 2022. **5**(5): p. e2210768-e2210768.

262. Jarosova, D., et al., *Job satisfaction and subjective well‐being among midwives: analysis of a multinational cross‐sectional survey.* Journal of midwifery & women's health, 2017. **62**(2): p. 180-189.

263. Lau, A.L., R.A. Cummins, and W. Mcpherson, *An investigation into the cross-cultural equivalence of the Personal Wellbeing Index.* Social Indicators Research, 2005. **72**: p. 403-430.

264. Lau, A.L., et al., *The SARS (Severe Acute Respiratory Syndrome) pandemic in Hong Kong: Effects on the subjective wellbeing of elderly and younger people.* Aging and mental health, 2008. **12**(6): p. 746-760.

265. Cummins, R.A., et al., *Developing a national index of subjective wellbeing: The Australian Unity Wellbeing Index.* Social indicators research, 2003. **64**: p. 159-190.

266. Lam, L.C., et al., *A randomized controlled trial to examine the effectiveness of case management model for community dwelling older persons with mild dementia in Hong Kong.* International Journal of Geriatric Psychiatry: A journal of the psychiatry of late life and allied sciences, 2010. **25**(4): p. 395-402.

267. Low, R.S., et al., *Emotion regulation and psychological and physical health during a nationwide COVID-19 lockdown.* Emotion, 2021. **21**(8): p. 1671.

268. Migliorini, C.E., M. Elfström, and B.J. Tonge, *Translation and Australian validation of the spinal cord lesion-related coping strategies and emotional wellbeing questionnaires.* Spinal Cord, 2008. **46**(10): p. 690-695.

269. Lawton, M.P., *The Philadelphia geriatric center morale scale: A revision.* Journal of gerontology, 1975. **30**(1): p. 85-89.

270. Togashi, Y., et al., *Effect of life-space mobility on subjective well-being in aged home-based rehabilitation users with different levels of independence in activities of daily living.* Journal of Physical Therapy Science, 2022. **34**(1): p. 18-21.

271. Komase, Y., et al., *Reliability and validity of the Japanese version of the Gratitude at Work Scale (GAWS).* journal of Occupational Health, 2020. **62**(1): p. e12185.

272. Stewart-Sicking, J.A., *Subjective well-being among Episcopal priests: Predictors and comparisons to non-clinical norms.* Journal of Prevention & Intervention in the Community, 2012. **40**(3): p. 180-193.

273. Eid, M. and R.J. Larsen, *The science of subjective well-being*. 2008: Guilford Press.

274. Hsieh, Y.L. and J.L. Lo, *Occupational Experiences and Subjective Well‐being of Mothers of Children with ASD in Taiwan.* Occupational therapy international, 2013. **20**(1): p. 45-53.

275. Cameron, J.I., et al., *What makes family caregivers happy during the first 2 years post stroke?* Stroke, 2014. **45**(4): p. 1084-1089.

276. Giarelli, E., *Perceived quality of work life and risk for compassion fatigue among oncology nurses: a mixed-methods study.* Number 3/May 2016, 2016. **43**(3): p. E121-E131.

277. Grove, J.R. and H. Prapavessis, *Preliminary evidence for the reliability and validity of an abbreviated profile of mood states.* International Journal of Sport Psychology, 1992.

278. Berger, B.G. and R.W. Motl, *Exercise and mood: A selective review and synthesis of research employing the profile of mood states.* Journal of applied sport psychology, 2000. **12**(1): p. 69-92.

279. Hergenroeder, A., et al., *Effect of a 6-month sedentary behavior reduction intervention on well-being and workplace health in desk workers with low back pain.* Work, 2022. **71**(4): p. 1145-1155.

280. Chassany, O., et al., *The psychological general well-being index (pgwbi) user manual.* Lyon, France: MAPI Research Institute, 2004.

281. Dagenais-Desmarais, V. and A. Savoie, *What is psychological well-being, really? A grassroots approach from the organizational sciences.* Journal of Happiness Studies, 2012. **13**: p. 659-684.

282. Teixeira, H., et al., *An exploratory study of perfectionism, professional factors and psychological well‐being of dentistry academics.* Australian Dental Journal, 2021. **66**(2): p. 175-181.

283. Medzo-M’Engone, J. and M. Ntsame Sima, *Psychometric properties of the psychological well-being at work scale in Gabonese public administration.* Journal of Evidence-Based Social Work, 2021. **18**(1): p. 101-115.

284. Rice, K.G., C.M. Richardson, and S. Tueller, *The short form of the revised almost perfect scale.* Journal of personality assessment, 2014. **96**(3): p. 368-379.

285. Yi, J., et al. *The effect of primary and middle school teachers’ problematic internet use and fear of Covid-19 on psychological need thwarting of online teaching and psychological distress*. in *Healthcare*. 2021. MDPI.

286. Widerszal-Bazyl, M. and M. Cieślak, *Monitoring psychosocial stress at work: development of the Psychosocial Working Conditions Questionnaire.* International Journal of Occupational Safety and Ergonomics, 2000. **6**(sup1): p. 59-70.

287. Misiak, B., et al., *Psychosocial work-related hazards and their relationship to the quality of life of nurses—A cross-sectional study.* International Journal of Environmental Research and Public Health, 2020. **17**(3): p. 755.

288. Weiner, M.F., et al., *The quality of life in late-stage dementia (QUALID) scale.* Journal of the American Medical Directors Association, 2000. **1**(3): p. 114-116.

289. Sjölund, B.M., A.G. Mamhidir, and M. Engström, *Pain prevalence among residents living in nursing homes and its association with quality of life and well‐being.* Scandinavian journal of caring sciences, 2021. **35**(4): p. 1332-1341.

290. Burckhardt, C., B. Archenholtz, and A. Bjelle, *Measuring the quality of life of women with rheumatoid arthritis or systemic lupus erythematosus: a Swedish version of the Quality of Life Scale (QOLS).* Scandinavian journal of rheumatology, 1992. **21**(4): p. 190-195.

291. Liedberg, G.M., C.S. Burckhardt, and C.M. Henriksson, *Validity and reliability testing of the Quality of Life Scale, Swedish version in women with fibromyalgia–statistical analyses.* Scandinavian Journal of Caring Sciences, 2005. **19**(1): p. 64-70.

292. Kaplan, R.M., W.J. Sieber, and T.G. Ganiats, *The quality of well-being scale: comparison of the interviewer-administered version with a self-administered questionnaire.* Psychology and Health, 1997. **12**(6): p. 783-791.

293. Kaplan, R.M. and J.P. Anderson, *A general health policy model: update and applications.* Health services research, 1988. **23**(2): p. 203.

294. Eskridge, S.L., et al., *Prosthesis satisfaction and quality of life in US service members with combat-related major lower-limb amputation.* Prosthetics and Orthotics International, 2022. **46**(1): p. 68-74.

295. Wingler, D. and R. Hector, *Demonstrating the effect of the built environment on staff health-related quality of life in ambulatory care environments.* HERD: Health Environments Research & Design Journal, 2015. **8**(4): p. 25-40.

296. Hays, R.D., C.D. Sherbourne, and R.M. Mazel, *The rand 36‐item health survey 1.0.* Health economics, 1993. **2**(3): p. 217-227.

297. Mountain, G., et al., *A preventative lifestyle intervention for older adults (lifestyle matters): a randomised controlled trial.* Age and ageing, 2017. **46**(4): p. 627-634.

298. Spitzer, R.L., et al., *Health-related quality of life in primary care patients with mental disorders: results from the PRIME-MD 1000 Study.* Jama, 1995. **274**(19): p. 1511-1517.

299. Brazier, J., et al., *Measuring and valuing health benefits for economic evaluation*. 2017: OXFORD university press.

300. De Jong-Gierveld, J. and F. Kamphuls, *The development of a Rasch-type loneliness scale.* Applied psychological measurement, 1985. **9**(3): p. 289-299.

301. Hays, R.D. and L.S. Morales, *The RAND-36 measure of health-related quality of life.* Annals of medicine, 2001. **33**(5): p. 350-357.

302. Salonsalmi, A., et al., *Joint association of socioeconomic circumstances and minor mental health problems with antidepressant medication.* European Journal of Public Health, 2022. **32**(4): p. 535-541.

303. Naber, A., L. Willhite, and W. Lucas Molitor, *Exploration of individualized goals and ergonomic modifications to address sedentary behaviors and perceived health and well-being among office workers.* Work, 2021. **68**(4): p. 1133-1142.

304. Rosenberg, D.E., et al., *Reliability and validity of the Sedentary Behavior Questionnaire (SBQ) for adults.* Journal of physical activity and health, 2010. **7**(6): p. 697-705.

305. Ryff, C.D., *Happiness is everything, or is it? Explorations on the meaning of psychological well-being.* Journal of personality and social psychology, 1989. **57**(6): p. 1069.

306. Palma-Candia, O., et al., *Understanding the occupational adaptation process and well-being of older adults in Magallanes (Chile): A qualitative study.* International Journal of Environmental Research and Public Health, 2019. **16**(19): p. 3640.

307. Toledano-González, A., T. Labajos-Manzanares, and D. Romero-Ayuso, *Well-being, self-efficacy and independence in older adults: a randomized trial of occupational therapy.* Archives of gerontology and geriatrics, 2019. **83**: p. 277-284.

308. Jang, M.H., S.Y. Gu, and Y.M. Jeong, *Role of coping styles in the relationship between nurses’ work stress and well‐being across career.* Journal of nursing scholarship, 2019. **51**(6): p. 699-707.

309. Sirigatti, S., et al., *Assessment of factor structure of Ryff’s Psychological Well-Being Scales in Italian adolescents.* Bollettino di Psicologia Applicata, 2009. **259**(56): p. 30-50.

310. Schwartz, C.E., et al., *Cognitive reserve and patient-reported outcomes in multiple sclerosis.* Multiple Sclerosis Journal, 2013. **19**(1): p. 87-105.

311. Piotrkowska, R., et al., *Satisfaction with life of oncology nurses in Poland.* International nursing review, 2019. **66**(3): p. 374-380.

312. Cho, E. and S. Jeon, *The role of empathy and psychological need satisfaction in pharmacy students’ burnout and well-being.* BMC medical education, 2019. **19**: p. 1-12.

313. Szabó, M., et al., *Psychological Well-Being of Australian Actors and Performing Artists: Life Satisfaction and Negative Affect.* Medical Problems of Performing Artists, 2022. **37**(2): p. 106-117.

314. Anaby, D., et al., *Participation and well-being among older adults living with chronic conditions.* Social indicators research, 2011. **100**: p. 171-183.

315. Barclay, L., et al., *Satisfaction with life, health and well-being: comparison between non-traumatic spinal cord dysfunction, traumatic spinal cord injury and Australian norms.* Spinal cord series and cases, 2019. **5**(1): p. 50.

316. Rosenberg, M., *Society and the adolescent self-image*. 2015: Princeton university press.

317. Linn, M.W. and B.S. Linn, *Self-evaluation of life function (self) scale: a short, comprehensive self-report of health for elderly adults.* Journal of Gerontology, 1984. **39**(5): p. 603-612.

318. Maynard, M., F. Neal-Smith, and L.D. Martin, *Health and well-being ratings of African American adults with sickle cell disease: Counseling service implications.* Occupational Therapy in Health Care, 1996. **10**(2): p. 73-87.

319. Pickard, A.S., et al., *Replicability of SF-36 summary scores by the SF-12 in stroke patients.* Stroke, 1999. **30**(6): p. 1213-1217.

320. Magnavita, N., et al., *Mental and physical well-being in oncology-hematology–unit personnel.* Archives of environmental & occupational health, 2018. **73**(6): p. 375-380.

321. Kelsall, H.L., et al., *Pain-related musculoskeletal disorders, psychological comorbidity, and the relationship with physical and mental well-being in Gulf War veterans.* PAIN®, 2014. **155**(4): p. 685-692.

322. Gresham, F.M. and S.N. Elliot, *Social skills rating system.* PsycTESTS Dataset, 1990.

323. Hsueh, J. and H. Yoshikawa, *Working nonstandard schedules and variable shifts in low-income families: associations with parental psychological well-being, family functioning, and child well-being.* Developmental psychology, 2007. **43**(3): p. 620.

324. Gang, W., H. Xu, and Z. Dajun, *Effects of occupational stress and psychological capital on occupational well-being among kindergarten teachers: effects of coping way and culture.* Studies of Psychology and Behavior, 2017. **15**(1): p. 83.

325. Guo, Q., et al., *Psychological capital and occupational well-being: mediating effects of work engagement among Chinese special education teachers.* Frontiers in Psychology, 2022. **13**: p. 847882.

326. Paloutzian, R.F. and C.W. Ellison, *Manual for the spiritual well-being scale.* Nyack, NY: Life Advance, 1991.

327. Duggleby, W., D. Cooper, and K. Penz, *Hope, self‐efficacy, spiritual well‐being and job satisfaction.* Journal of advanced nursing, 2009. **65**(11): p. 2376-2385.

328. Malinakova, K., et al., *The Spiritual Well-Being Scale: Psychometric evaluation of the shortened version in Czech adolescents.* Journal of religion and health, 2017. **56**: p. 697-705.

329. Zidkova, R., et al., *Spirituality, religious attendance and health complaints in czech adolescents.* International journal of environmental research and public health, 2020. **17**(7): p. 2339.

330. Spielberger, C., *STAXI-2: State-Trait Anger Expression Inventory-2. Professional manual. Odessa, FL: Psychological Assessment Resources.* Inc.[Google Scholar], 1999.

331. Kvaal, K., et al., *The Spielberger state‐trait anxiety inventory (STAI): the state scale in detecting mental disorders in geriatric patients.* International Journal of Geriatric Psychiatry: A journal of the psychiatry of late life and allied sciences, 2005. **20**(7): p. 629-634.

332. Herrmann-Werner, A., et al., *Medical education in times of COVID-19: survey on teachers' perspectives from a German medical faculty.* GMS Journal for Medical Education, 2021. **38**(5).

333. Kessler, R.C., G. Borges, and E.E. Walters, *Prevalence of and risk factors for lifetime suicide attempts in the National Comorbidity Survey.* Archives of general psychiatry, 1999. **56**(7): p. 617-626.

334. Lindfors, P.M., et al., *Suicidality among Finnish anaesthesiologists.* Acta Anaesthesiologica Scandinavica, 2009. **53**(8): p. 1027-1035.

335. Farzanfar, R., et al., *Workplace telecommunications technology to identify mental health disorders and facilitate self-help or professional referrals.* American Journal of Health Promotion, 2011. **25**(3): p. 207-216.

336. Watanabe, K., et al., *Measuring eudemonic well-being at work: a validation study for the 24-item the University of Tokyo Occupational Mental Health (TOMH) well-being scale among Japanese workers.* Industrial health, 2020. **58**(2): p. 107-131.

337. Utian, W.H., et al., *The Utian Quality of Life (UQOL) Scale: development and validation of an instrument to quantify quality of life through and beyond menopause.* Menopause, 2018. **25**(11): p. 1224-1231.

338. Ojala, B., et al., *A cognitive behavioural intervention programme to improve psychological well-being.* International journal of environmental research and public health, 2019. **16**(1): p. 80.

339. Näätänen, P., et al., *Bergen Burnout Indicator-15.* 2003.

340. Tennant, R., et al., *Health and Quality of Life Outcomes.* The Warwick-Edinburgh Mental Well-being Scale (WEMWBS): Development and UK Validation, 2007.

341. Trousselard, M., et al., *Validation of the Warwick-Edinburgh mental well-being scale (WEMWBS) in French psychiatric and general populations.* Psychiatry research, 2016. **245**: p. 282-290.

342. Carson, F., et al., *Examining the mental well-being of Australian sport coaches.* International journal of environmental research and public health, 2019. **16**(23): p. 4601.

343. Puig-Ribera, A., et al., *Impact of a workplace ‘sit less, move more’program on efficiency-related outcomes of office employees.* BMC Public Health, 2017. **17**(1): p. 1-11.

344. Bartram, D.J., J.M. Sinclair, and D.S. Baldwin, *Further validation of the Warwick-Edinburgh Mental Well-being Scale (WEMWBS) in the UK veterinary profession: Rasch analysis.* Quality of Life Research, 2013. **22**: p. 379-391.

345. Rice, S.M., et al., *Athlete experiences of shame and guilt: initial psychometric properties of the athletic perceptions of performance scale within junior elite cricketers.* Frontiers in Psychology, 2021. **12**: p. 581914.

346. Marshall, C.A., et al., *‘The Big Island Model’: Resident experiences of a novel permanent supportive housing model for responding to rural homelessness.* Health & Social Care in the Community, 2022. **30**(6): p. e5047-e5061.

347. Taggart, F., et al., *Cross cultural evaluation of the Warwick-Edinburgh mental well-being scale (WEMWBS)-a mixed methods study.* Health and Quality of Life Outcomes, 2013. **11**(1): p. 1-12.

348. Bartram, D.J., et al., *Validation of the Warwick–Edinburgh Mental Well-being Scale (WEMWBS) as an overall indicator of population mental health and well-being in the UK veterinary profession.* The Veterinary Journal, 2011. **187**(3): p. 397-398.

349. Ahmad, W., et al., *Exploring diet, exercise, chronic illnesses, occupational stressors and mental well-being of healthcare professionals in Punjab, Pakistan.* BMC research notes, 2017. **10**: p. 1-3.

350. Summers, E.M., et al., *A survey of psychological practitioner workplace well‐being.* Clinical Psychology & Psychotherapy, 2021. **28**(2): p. 438-451.

351. Ng, S.S., et al., *Translation and validation of the Chinese version of the short Warwick-Edinburgh mental well-being scale for patients with mental illness in Hong Kong.* East Asian Archives of Psychiatry, 2014. **24**(1): p. 3-9.

352. Xu, H., et al., *Effects of mobile mindfulness on emergency department work stress: A randomised controlled trial.* Emergency Medicine Australasia, 2022. **34**(2): p. 176-185.

353. Lewis, E.G. and J.M. Cardwell, *The big five personality traits, perfectionism and their association with mental health among UK students on professional degree programmes.* BMC psychology, 2020. **8**: p. 1-10.

354. Karpavičiūtė, S. and J. Macijauskienė, *The impact of arts activity on nursing staff well-being: An intervention in the workplace.* International Journal of Environmental Research and Public Health, 2016. **13**(4): p. 435.

355. Colucci, E., et al., *COVID-19 lockdowns’ effects on the quality of life, perceived health and well-being of healthy elderly individuals: A longitudinal comparison of pre-lockdown and lockdown states of well-being.* Archives of gerontology and geriatrics, 2022. **99**: p. 104606.

356. Cousins*, R., et al., *‘Management standards’ work-related stress in the UK: Practical development.* Work & Stress, 2004. **18**(2): p. 113-136.

357. Paykel, E.S., et al., *Suicidal feelings in the general population: a prevalence study.* The British Journal of Psychiatry, 1974. **124**(582): p. 460-469.

358. Bush, K., et al., *The AUDIT alcohol consumption questions (AUDIT-C): an effective brief screening test for problem drinking.* Archives of internal medicine, 1998. **158**(16): p. 1789-1795.

359. Chu, M.H.-w., et al., *Positive psychotherapy for psychosis in Hong Kong: A randomized controlled trial.* Schizophrenia research, 2022. **240**: p. 175-183.

360. Schlosser, B., *The assessment of subjective well-being and its relationship to the stress process.* Journal of Personality Assessment, 1990. **54**(1-2): p. 128-140.

361. Myers, N.D., D.L. Feltz, and E.W. Wolfe, *A confirmatory study of rating scale category effectiveness for the coaching efficacy scale.* Research quarterly for exercise and sport, 2008. **79**(3): p. 300-311.

362. Williams, G., K. Thomas, and A. Smith, *Stress and well-being of University Staff: an investigation using the Demands-Resources-Individual Effects (DRIVE) model and Well-being Process Questionnaire (WPQ).* Psychology, 2017. **8**(12): p. 1919-1940.

363. Williams, G. and A.P. Smith, *Diagnostic validity of the anxiety and depression questions from the Well-Being Process Questionnaire.* Journal of Clinical and Translational Research, 2019. **4**(2): p. 101.

364. Hayes, B., et al., *What’s up doc? A national cross-sectional study of psychological wellbeing of hospital doctors in Ireland.* BMJ open, 2017. **7**(10): p. e018023.

365. Ward, M., et al., *One Island–One Lifestyle? Health and lifestyles in the Republic of Ireland and Northern Ireland.* 2009.

366. Krieger, T., et al., *Measuring depression with a well-being index: further evidence for the validity of the WHO Well-Being Index (WHO-5) as a measure of the severity of depression.* Journal of affective disorders, 2014. **156**: p. 240-244.

367. Rao, N. and K.J. Kemper, *Online training in specific meditation practices improves gratitude, well-being, self-compassion, and confidence in providing compassionate care among health professionals.* Journal of Evidence-Based Complementary & Alternative Medicine, 2017. **22**(2): p. 237-241.

368. Popova, E.S., et al., *Exploring well-being: Resilience, stress, and self-care in occupational therapy practitioners and students.* OTJR: Occupational Therapy Journal of Research, 2023. **43**(2): p. 159-169.

369. Abo-Ali, E.A., et al., *Mental well-being and self-efficacy of healthcare workers in Saudi Arabia during the COVID-19 pandemic.* Risk Management and Healthcare Policy, 2021: p. 3167-3177.

370. Eiche, C., et al., *Well-being and PTSD in German emergency medical services–A nationwide cross-sectional survey.* PLoS One, 2019. **14**(7): p. e0220154.

371. Biering-Sørensen, B., et al., *Treatment diary for botulinum toxin spasticity treatment: a pilot study.* International journal of rehabilitation research. Internationale Zeitschrift fur Rehabilitationsforschung. Revue internationale de recherches de readaptation, 2017. **40**(2): p. 175.

372. Schougaard, L.M.V., et al., *Test-retest reliability and measurement error of the Danish WHO-5 Well-being Index in outpatients with epilepsy.* Health and quality of life outcomes, 2018. **16**: p. 1-6.

373. Beaudequin, D., et al., *Relationships between reduction in symptoms and restoration of function and wellbeing: Outcomes of the Oral Ketamine Trial on Suicidality (OKTOS).* Psychiatry Research, 2021. **305**: p. 114212.

374. Bahadır Ağce, Z. and G. Ekici, *Person-centred, occupation-based intervention program supported with problem-solving therapy for type 2 diabetes: a randomized controlled trial.* Health and Quality of Life Outcomes, 2020. **18**(1): p. 1-14.

375. Freak-Poli, R.L., et al., *Change in well-being amongst participants in a four-month pedometer-based workplace health program.* BMC Public Health, 2014. **14**(1): p. 1-10.

376. Kolind, M.I., et al., *Daily life coping—Helping stress-afflicted people manage everyday activities.* Scandinavian Journal of Occupational Therapy, 2023. **30**(2): p. 170-181.

377. Rees, C., et al., *Mindful Self-Care and Resiliency (MSCR): protocol for a pilot trial of a brief mindfulness intervention to promote occupational resilience in rural general practitioners.* BMJ open, 2018. **8**(6): p. e021027.

378. Addley, K., C. Burke, and P. McQuillan, *Impact of a direct access occupational physiotherapy treatment service.* Occupational medicine, 2010. **60**(8): p. 651-653.

379. Stuber, F., et al., *Feasibility, psychological outcomes and practical use of a stress-preventive leadership intervention in the workplace hospital: the results of a mixed-method phase-II study.* BMJ open, 2022. **12**(2): p. e049951.

380. Sayed Ahmed, H.A., et al., *Psychometric properties of the Arabic version of the problem areas in diabetes scale in primary care.* Frontiers in Public Health, 2022. **10**: p. 843164.

381. Marchand, A., V.Y. Haines, and J. Dextras-Gauthier, *Quantitative analysis of organizational culture in occupational health research: a theory-based validation in 30 workplaces of the organizational culture profile instrument.* BMC Public Health, 2013. **13**(1): p. 1-11.

382. Wang, C., et al., *Effects of a mutual recovery intervention on mental health in depressed elderly community-dwelling adults: a pilot study.* BMC public health, 2017. **17**: p. 1-10.

383. Vang, M.L., J. Pihl‐Thingvad, and M. Shevlin, *Identifying child protection workers at risk for secondary traumatization: A latent class analysis of the Professional Quality of Life Scale–5.* Journal of Traumatic Stress, 2022. **35**(6): p. 1608-1619.

384. De Kock, J.H., et al., *Brief digital interventions to support the psychological well-being of NHS staff during the COVID-19 pandemic: 3-Arm pilot randomized controlled trial.* JMIR mental health, 2022. **9**(4): p. e34002.

385. Cooper, K. and G.C. Barton, *An exploration of physical activity and wellbeing in university employees.* Perspectives in public health, 2016. **136**(3): p. 152-160.

386. Feicht, T., et al., *Evaluation of a seven-week web-based happiness training to improve psychological well-being, reduce stress, and enhance mindfulness and flourishing: a randomized controlled occupational health study.* Evidence-Based Complementary and Alternative Medicine, 2013. **2013**.

387. McCullough, M.E., R.A. Emmons, and J.-A. Tsang, *The grateful disposition: a conceptual and empirical topography.* Journal of personality and social psychology, 2002. **82**(1): p. 112.

388. Kemper, K.J., G. Gascon, and J.D. Mahan, *Two new scales for integrative medical education and research: confidence in providing calm, compassionate care scale (CCCS) and self-efficacy in providing non-drug therapies (SEND) to relieve common symptoms.* European Journal of Integrative Medicine, 2015. **7**(4): p. 389-395.

389. Tuomi, K., et al., *Work ability index*. Vol. 19. 1998: Finnish Institute of Occupational Health Helsinki.

390. Van Laar, D., J.A. Edwards, and S. Easton, *The Work‐Related Quality of Life scale for healthcare workers.* Journal of advanced nursing, 2007. **60**(3): p. 325-333.

391. Parker, G.B. and M.P. Hyett, *Measurement of well-being in the workplace: The development of the Work Well-Being Questionnaire.* The Journal of nervous and mental disease, 2011. **199**(6): p. 394-397.

392. Garzaro, G., et al., *A contribution to the validation of the Italian version of the work-related quality of life scale.* La Medicina del lavoro, 2020. **111**(1): p. 32.

393. Padua, R., et al., *Italian version of the Disability of the Arm, Shoulder and Hand (DASH) questionnaire. Cross-cultural adaptation and validation.* Journal of Hand Surgery, 2003. **28**(2): p. 179-186.

394. Sulaiman, N.S., et al., *Assessing quality of working life among Malaysian workers.* Asia Pacific Journal of Public Health, 2015. **27**(8_suppl): p. 94S-100S.
